# The role of framing, agency and uncertainty in a focus-divide dilemma

**DOI:** 10.3758/s13421-023-01484-6

**Published:** 2023-11-03

**Authors:** Justin Claydon, Warren R. G. James, Alasdair D. F. Clarke, Amelia R. Hunt

**Affiliations:** 1 School of Psychology, University of Aberdeen, Aberdeen, AB24 3UB UK; 2 School of Medical Sciences, University of Aberdeen, Aberdeen, UK; 3 School of Psychology, University of Essex, Essex, UK

**Keywords:** Decision making, Agency, Framing, Uncertainty, Judgement

## Abstract

How to prioritise multiple objectives is a common dilemma of daily life. A simple and effective decision rule is to focus resources when the tasks are difficult, and divide when tasks are easy. Nonetheless, in experimental paradigms of this dilemma, participants make highly variable and suboptimal strategic decisions when asked to allocate resources to two competing goals that vary in difficulty. We developed a new version in which participants had to choose where to park a fire truck between houses of varying distances apart. Unlike in the previous versions of the dilemma, participants approached the optimal strategy in this task. Three key differences between the fire truck version and previous versions of the task were investigated: (1) *Framing* (whether the objectives are familiar or abstract), by comparing a group who placed cartoon trucks between houses to a group performing the same task with abstract shapes; (2) *Agency* (how much of the task is under the participants’ direct control), by comparing groups who controlled the movement of the truck to those who did not; (3) *Uncertainty,* by adding variability to the driving speed of the truck to make success or failure on a given trial more difficult to predict. Framing and agency did not influence strategic decisions. When adding variability to outcomes, however, decisions shifted away from optimal. The results suggest choices become more variable when the outcome is less certain, consistent with exploration of response alternatives triggered by an inability to predict success.

**Supplementary Information:**

The online version contains supplementary material available at 10.3758/s13421-023-01484-6.

## Introduction

A student can divide their study time between all possible exam topics, or focus their efforts, learning a subset in depth. A basketball player can use zone or man-to-man defence. A government agency could fund one large public project or two smaller ones. We frequently encounter focus-divide dilemmas in everyday life. The best course of action in these scenarios depends on the ability of the decision maker and the difficulty of competing tasks. When it is possible to complete multiple tasks successfully, this is the best option, but with increasing difficulty there comes a point where focusing on a single task yields better results. This reflects a simple decision rule for optimal performance: focus when difficult, divide when easy. Surprisingly, previous research has demonstrated that deviation from this simple decision rule is typical, to the extent that participants exhibit a near-total failure to adjust their strategy with changes in task difficulty (Clarke & Hunt, 2016; Hunt et al., 2019; James et al., 2017; James et al., 2019; James et al., 2023; Morvan & Maloney, 2012). In this series of experiments, we present a new version of the focus-divide dilemma in which participants approach the optimal strategy, and test whether agency over elements of the task or uncertainty about choice outcomes underlie the difference from previous results.

A large and diverse literature addresses the question of how people make decisions about resource allocation, particularly under conditions of scarcity and uncertainty. The broad implications for policy and interventions around global issues such as poverty and disaster relief mean that much of this research has been conducted in the context of social circumstances and economic resources. Scarcity Theory (Shah et al., 2012), for example, addresses how manipulations of resources influence the attentional focus and priorities reflected in people’s decisions. By randomly assigning individuals to “poor” or “rich” conditions and measuring the group differences, this research area has revealed some of the causal mechanisms that could underlie economic decision-making with social causes and consequences. We have approached the question of decisions under resource constraints from a very different direction, which is how we allocate *visual* resources (that is, the small area in our central vision in which our visual acuity is highest) under conditions where targets are more or less easy to see in the periphery. We adapt a paradigm originally devised by Morvan and Maloney (2012), who presented participants with two possible locations in which an upcoming target would appear and asked them to choose a place to fixate. When the two locations are close together, participants should fixate equidistant between them and use their peripheral vision to spot the target, but when the locations are too far apart for both locations to be clearly visible from here, they should switch to selecting one location or the other. Surprisingly, participants did not even approach this strategy and instead made fixation choices that were unrelated to the distance between the possible locations of the target. Although this experiment about eye movement control may sound far removed from questions about resource allocation, Clarke and Hunt (2016) generalised the paradigm and showed a similar pattern across diverse tasks described below. Optimal outcomes in these experiments require adjusting decisions appropriately in response to changes in resource requirements, and participants are surprisingly poor at making these adjustments across a wide range of experimental contexts. Our initial goal in the current research was to broaden the context to include larger and more diverse samples as a further step in that direction.

Clarke and Hunt’s (2016) original focus-divide dilemma paper examined whether suboptimal and idiosyncratic fixation selection (Morvan & Maloney, 2012) would extend beyond eye-movements to more deliberative decisions. They examined strategy in tasks with the same underlying choice problem that differed in modality: *detection* (replicating the original fixation selection task described above), *throwing*, *memory*, and *reaching*. The *throwing* experiment presented participants with two target hoops which varied in separation. Before they were told which was the target, they had to choose a place to stand. After they chose, they were told which hoop to aim for, and they threw a beanbag to try and get it in the specified hoop. When the hoops are close together, standing in the middle yields high throwing accuracy for both targets. As hoop separation increases, accuracy falls. Once accuracy dips below 50%, standing adjacent to one of the target hoops is better than the centre (given the 50% chance of correctly guessing which hoop will be designated as the target). Despite the simplicity of this strategy to understand and implement once known (Hunt et al., 2019), most participants fail to modify their strategy in response to changes in distance. A similar pattern was observed in the *memory* version of the dilemma. Participants were asked to report one of two digit strings presented on either side of a monitor. The number of digits in the two strings varied from trial to trial. The best strategy was to memorise both when the strings were short and switch to focusing on a single string when they were too long for it to be possible to memorise both. Here again, participants failed to adjust their strategy with difficulty. The results of the *throwing*, *memory* and *detection* tasks demonstrate a consistent failure to make optimal-focus divide decisions that generalises across different modalities.

Why are people unable to apply what seems to be a relatively simple decision strategy in these experiments? Cognitive bias, a simple lack of information, and a lack of motivation to improve at the task have all been considered as possible explanations. First, a single cognitive bias such as risk aversion (Pratt, 1978) or loss aversion (Kahneman & Tversky, 1984) is unlikely because this would predict a consistent but suboptimal behavioural pattern; for example, if participants avoided the risk of selecting a single target, it would drive them to always divide. A consistent pattern is not observed: some participants consistently divide resources between both goals, some focus on one rather than the other, and most exhibit variation in strategy between these two extremes, but are not influenced systematically by changes in the difficulty of the task (see James et al., 2023 for a more detailed argument on this). Second, a possible lack of information, in line with Bounded Rationality (Simon, 1990), was assessed by James et al. (2017). They asked participants to explicitly report their probability of success and found participants’ predicted and actual performance were closely matched. Despite clearly having the necessary information to make optimal decisions (and even calling attention to this information, by asking participants to explicitly report it), participants' strategic decisions were highly variable and suboptimal. Third, motivation (or the lack of it) was evaluated by James et al. (2019) using gamification, with the logic that enjoyable, game-like experiments encourage effortful attention to accuracy rather than a focus on completing the experiment as fast as possible (Miranda & Palmer, 2014). Participants in the gamified version of the task had higher success rates than those who completed a non-gamified version. Importantly, however, these improvements were *not* driven by adopting a better strategy. Motivated participants instead exerted more effort into non-strategic elements of the task, such as minimising response errors. Strategies were unchanged, despite the fact that large performance gains could have been made if strategies had improved. That participants fail to adopt a better strategy, despite demonstrating sufficient motivation to work harder, suggests that poor strategy in the focus-divide problem is a persistent default.

Understanding the failure to apply an appropriate strategy in the focus-divide dilemma may therefore require accounting for the widely variable choices both between and within participants. To that end, our initial aim was to develop a version of the focus-divide dilemma suitable for recruiting a large online sample to examine individual differences that might explain variation in strategy. In the task we developed, participants must decide where to place a virtual fire truck between two houses that vary in separation. After placing the truck, one of the houses catches fire. The goal is to position the truck to reach the burning house as quickly as possible, without knowing which one of the two will catch fire. This dilemma has the same optimal strategy as previous focus-divide paradigms (*throwing*, *detection*, *memory* and *reaching*; Clarke & Hunt, 2016). When the houses are close, it is best to place the truck midway between them, so that either one can be reached if it catches fire. As the separation increases there is a point at which the truck can no longer reach either house from the centre in time. Past that point, placing the truck adjacent to one of the houses yields a higher success rate than placing it in the middle.

To foreshadow the results, in Experiment 1 of the current report, we implemented the fire trucks task and found that unlike previous versions of the focus-divide dilemma (e.g., Clarke & Hunt, 2016; James et al., 2017; James et al., 2019; James et al., 2023; Morvan & Maloney, 2012), participants made choices that were closer to optimal. For the investigation of individual differences that was our original motivation, this is a limitation because participants are more uniform by virtue of being closer to optimal. This makes the differences between individuals less meaningful (Hedge et al., 2018). On the other hand, because choices improve in the fire trucks task relative to other versions of the dilemma, the task provides an even more useful opportunity: by exploring the factors that distinguish it from previous versions, we can shed light on which of these is responsible for the improved performance and thereby identify the basis for previous errors in focus-divide decisions.

Three main differences between the fire trucks and the other iterations of the *focus-divide* paradigm are explored in this series of experiments: framing, agency and uncertainty. *Framing* is the “story”, that is, participants may understandably feel more urgency around preventing houses from burning down than they do about getting a beanbag into a hoop, or detecting a target, or remembering a string of digits. There is some precedent that the format of information can influence problem solving. For example, natural frequencies are more easily and accurately used relative to the same information presented as probabilities (e.g., Gigerenzer & Hoffrage, 1995), and participants are more likely to solve a deductive reasoning problem when the “thematic materials” are familiar than when they are abstract (Wason & Shapiro, 1971). Previous focus-divide experiments are not abstract in the sense of presenting hypothetical or symbolic material, or in requiring people to interpret probability information, but the fire truck version of the problem is possibly framing the information in a way that taps into problem-solving the participant has done successfully before. Cox and Griggs (1982) demonstrated the thematic material effect described above is observed even when shifting the problem from deciding if a rule about age limits on drinking alcohol has been violated (a familiar problem) relative to deciding if a rule about age limits on wearing a particular colour has been violated (the same problem, but less familiar). A similarly subtle shift from a concrete but unfamiliar problem (like deciding where to stand to throw a beanbag in a hoop) to a hypothetical but familiar problem (like deciding where to park a cartoon fire truck) could influence problem-solving in the focus-divide dilemma.

*Agency* relates to the aspects of the task under the control of the participant. A common feature of past research is that participants had agency over both the strategy and performance elements of the task (Clarke & Hunt, 2016; Hunt et al., 2019; James et al., 2017). For example, in the throwing task, participants not only choose where to stand, but physically perform the throw on each trial. In the fire trucks task, agency is restricted to just the strategic decision. Participants only control where to place the avatar, after which it moves to the target. This feature alone could allow them to focus their attention more completely on the strategic decision, but over and above this, there is also evidence that people weigh information (Kray & Gonzales, 1999) and risk (Polman & Wu, 2020) differently when making decisions on behalf of others compared to when they make decisions for themselves.

*Uncertainty* refers to the probability of success given the focus-divide choice. In most other versions of the focus-divide paradigm, probability of success gradually declines as the difficulty of tasks increases, but the outcome on any given trial depends on complex, noisy variation in the environment and the person’s task execution that means sometimes expectations are violated. There is evidence from other decision-making contexts that the presence and nature of uncertainty influences the variability of decision-making, such that more uncertainty adds random variation to response selection (Wilson et al., 2014; Gershman, 2019). In previous versions of the focus-divide dilemma, it was not possible to manipulate and measure the effect of uncertainty because the uncertainty was caused by uncontrollable factors. The fire trucks task, in contrast, allows us to directly test the effects of uncertainty by manipulating the variability in truck speed from trial to trial.

The three experiments presented here measure the effects of task framing, agency, and uncertainty on the optimality of focus-divide decisions. The key measure is *Adjustment*, which is the difference in where participants place the truck when the houses are close together relative to when they are far apart, with 0 reflecting random and 1 reflecting perfectly optimal adaptation of choice to the distance conditions. Experiment 1 compares two groups of participants, one performing the task with fire trucks, and the other performing a task with the same structure and parameters but with simple shapes in place of trucks and houses. Experiment 2 increases the participants’ agency over the truck’s movement and compares their adjustment with distance back to that of the fire truck group in Experiment 1. Experiment 3 increases the complexity of the driving task as a stronger manipulation of agency. Across all three experiments, we also measure the effects of uncertainty by comparing a condition where the speed of the truck was constant, and so the outcome of each trial is predictable, to a condition where the speed of the truck varies from trial to trial. The full set of experiments and their outcomes are summarised in Table 1.

**Table 1:** List of experiments and result

Exp.	Between-group factor(s)	Within-group factor	N	Key result
1	Framing (firetruck scenario vs abstractshapes)	Speed type (constant vs variables)	60	Variability in truck speed reduces choice optimality; no effect of framing
2	Agency over truck motion: simple button press compared to automatic movement group in E1	Speed type (constant vs varibales)	30	Variability in truck speed reduces choice optimality no effect of agency
3	Agency over truck motion: complex control scheme vs authomatic movement	None: minimised trials for internet-based testing	126	Variability in truck speed reduces choice optimality: no effect of agency

*Note*: The within-group factor of speed type was blocked and counterbalnaced. An analysis of the order effect accross both E1 and E2 is presented at the end of the E2 results section.

## Experiment 1: Task framing and uncertainty

To make the task and the appropriate decision easy to understand, we framed the problem using a practical choice of where to park a fire truck. To check whether this framing mattered, we also included a version of the task that was more abstract, but otherwise identical. Framing was manipulated between groups of participants. We also manipulated uncertainty, to test a hypothesis suggested by an observation in Clarke and Hunt (2016): they included a *reaching* task, which poses the same focus-divide dilemma, but in a scenario where outcomes are certain. Participants choose where to sit at a long table before reaching to one (of two) beanbags that varied in separation. Participants uniformly followed the optimal strategy when choosing a seat, unlike in all other versions of the task, suggesting a potential role for certainty in suboptimal choice: it is more certain whether you can reach a given distance from a particular chair than whether you can throw accurately from a particular distance. To test this more directly, we manipulated the motion of the virtual avatar in the fire trucks task, such that the chance of success was either a probability that decreased gradually with difficulty, similar to the *throwing* task, or followed a step function, similar to *reaching* (Clarke & Hunt, 2016). We also asked participants to report on their certainty about the truck’s chance of successfully reaching the house from different distances away to see if greater certainty was related to more optimal choices.

For the effects of both framing and uncertainty, the basis for comparison is the previous results from the focus-divide dilemma, in which only a small minority of participants are making choices that follow the optimal strategy of focusing when the tasks are difficult and dividing when they are easy. If either framing or uncertainty is responsible for poor choices observed previously on these tasks, we should see a decline in success rates and an increase in the variability of choice between participants when the task becomes more abstract and/or there is an increase in outcome uncertainty.

### Method

#### Participants

The sample of 60 participants (19 male) had a mean age of 22.1 years (between 17 and 36 years). Participants were recruited from a first-year introductory psychology course at the University of Aberdeen (*N* = 38) and from a cohort of Master’s students (*N* = 22). All experiment protocols in this paper were reviewed and approved by the Aberdeen Psychology Ethics Committee.

#### Justification of sample size

The sample size for this experiment was based on a power analysis as described by James and colleagues (2023). They used bootstrapping methods from previously collected focus-divide task datasets to simulate a small shift in position choices (0.05 of the normalised range) at different sample sizes. These simulations show uncertainty around estimates of this small shift plateau at approximately *N* = 15. We recruited 30 to each group to ensure we were far above this plateau.

#### Procedure

**Fig. 1:**
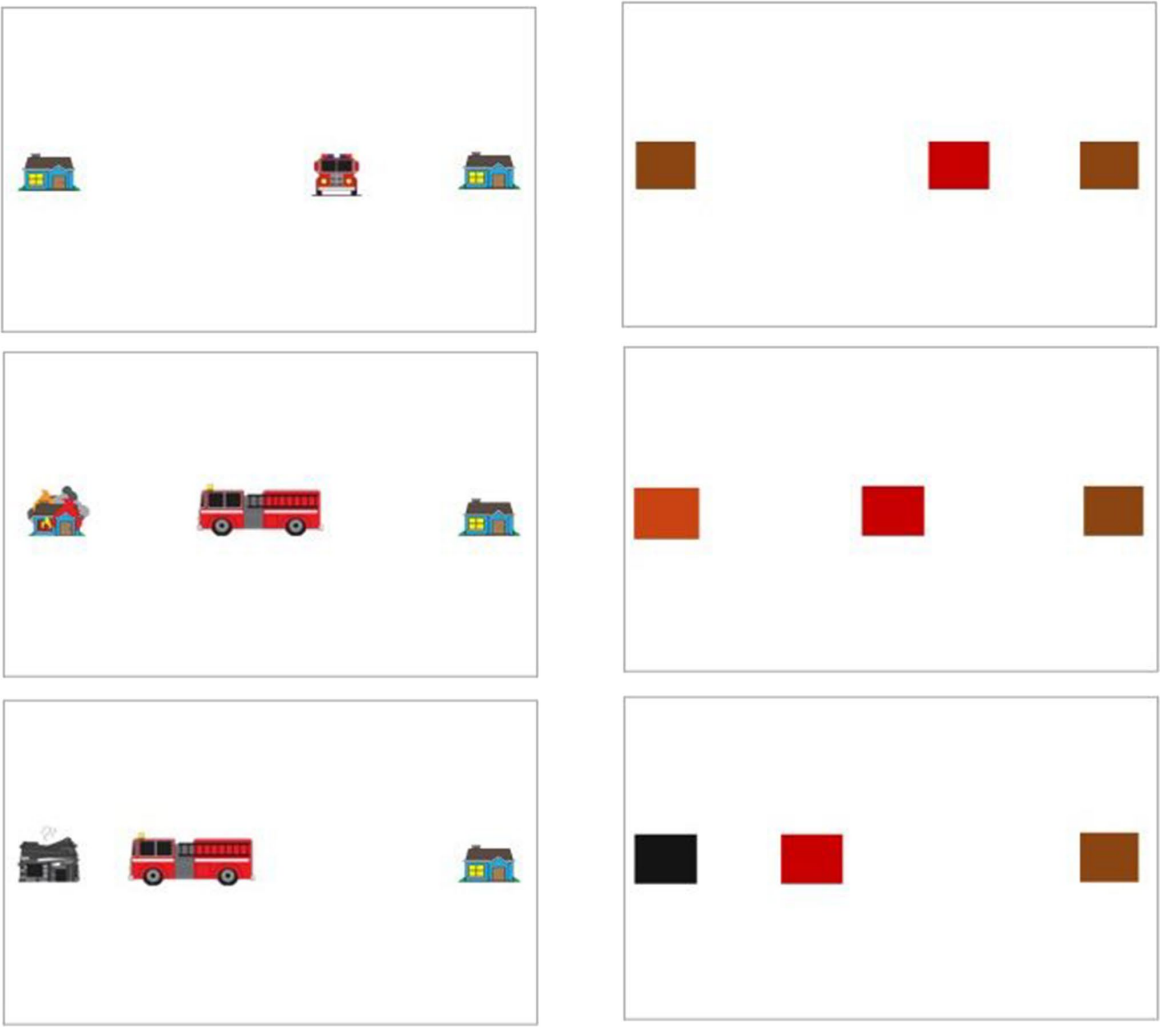
Example decision trial in both the Truck (left column) and Abstract (right column) conditions. In the Truck condition (left column), the avatar was a fire truck and the targets were houses. In the Abstract condition (right), the avatar and targets were coloured squares. In these examples, the avatar failed to reach the target before the time limit

Convenience sampling resulted in two different settings in which participants completed the experiment. The first 38 participants were recruited in a group setting from an undergraduate practical session in the School of Psychology at the University of Aberdeen (referred to as “grouped participants” below). All these participants sat at individual desks in a computer lab. The remaining 22 participants were recruited via word of mouth from a cohort of Masters students, and completed the task in a room on their own (“solo participants”). Other than this difference, the 60 participants were given one of two versions of the same task, as described below. The experiment was programmed in Matlab version 9.0.0 (R2016a) using Psychtoolbox functions (Brainard, 1997; Pelli, 1997). For the grouped participants, the stimuli were displayed on a 48 × 26.7 cm HP elite display e202 monitor with a screen resolution of 1,600 × 900 and a refresh rate of 60 Hz. For the solo participants, the stimuli were displayed on an 59 cm × 33 cm AOC AG271QX monitor with a screen resolution of 1,920 × 1,080 and a refresh rate of 60 Hz. Framing was manipulated between subjects, and one half of the sample were assigned to either the Truck or Abstract condition (see Fig. 1). In the Abstract condition, two brown squares appeared on screen, and participants were told one of them would turn orange. Their task was to choose where to place the red square, with the goal of reaching the orange target before it turned black. In the more concrete version of the task, the brown squares were houses, the red square was a fire truck, and the participants were told that one of the houses would catch fire. Their task was to choose where to park the fire truck so it could reach the house that caught fire before it burnt down. This is referred to as the Truck condition in the results and figures, and the version with the squares as the Abstract condition.

There were three phases which were completed in a set order: The Learning phase, the Decision phase, then the Estimation phase. These three phases were repeated twice: once with the truck/red square avatar travelling at constant speeds and once with variable speeds (order counterbalanced). In the Constant condition, the speed of the avatar was set to a fixed value of 3.5 pixels per frame. This meant that the avatar would always travel a specific distance, and any targets within this range would be reached in time, whereas those beyond this range would be missed. In the Variable condition, avatar speed was selected at random between a maximum and minimum speed limit of 2 and 5 pixels per frame. All speeds within this range in steps of 0.5 had an equal chance of occurring on any trial.

##### Learning phase

A single target and the Avatar were presented on screen at one of four distances from screen centre. The minimum distance was 200 pixels. The maximum value was determined as half the width of the monitor (in pixels) minus 100. For example, for monitor resolution 1,920 × 1,080, the maximum value would be: 1,920/2-100 = 860. Participants were instructed to press the spacebar whenever they were ready to start the trial. A target and the avatar appeared on screen. After a random time between 1 and 2.5 s, the target would “ignite” or change from brown to orange and the avatar would automatically move towards the target. There was a set time limit of ~1.6 s for the avatar to reach the target. If the avatar did not reach the target in time, the target would “burn down” (Truck) or change colour to a black box (Abstract) to indicate a failure to reach the target in time (see Fig. 1). Each of the four distances was repeated ten times in sequence in ascending order until all trials had finished, giving 40 trials in the learning phase for each of the two Speed type conditions.

##### Decision phase

Participants were instructed using a short animation. Each trial began with two objects (squares or houses, the framing manipulation) that were both placed at one of four equal distances to the left and right of the centre of the screen (the distance manipulation, as described in the Learning phase). The avatar was positioned in the top half of the screen and somewhere in the middle third of the distance between the two objects. For example, if the objects were both 150 pixels from the centre, the truck could be initially positioned at most 50 pixels from the horizontal centre of the screen either to the left or right. Participants were told that each of the two objects had an equal chance to become the target after they had decided where to position the avatar, and that their job was to position the avatar to reach that target on each trial. Participants used mouse clicks to select any position on the same horizontal plane as the potential targets, including outside of the range between them, after which they were told to press the spacebar to start the trial. A random time between 1 and 2.5 s would pass before the target was specified (caught fire, turned orange) and then after 317 ms the avatar would move automatically towards the target. The speed of the avatar was either constant or variable depending on the speed condition, as described in the general procedure above. Success and failure on each trial was indicated in the same way as the learning phase, with the same deadline of 1.6 s. Fifteen trials at each distance were displayed in a randomly shuffled order totalling 60 trials for this section. After each set of 30 trials, participants were given a break and summary feedback (what percentage of targets they had reached in time).

##### Estimation phase

This phase was designed to test the accuracy of participants’ understanding of the avatar’s performance. We explored two different methods, to which participants were randomly assigned. In both versions, participants were shown the avatar and one target some distance apart. In one version they were asked “How many times out of 10 do you think the truck/square would reach the target in time?” and simply had to adjust the starting value (5) up or down by using the arrow keys. In the other version, participants were asked “Will the (Avatar) reach its target?” and were to respond by pressing the y or n keys to answer “Yes” or “No” respectively, after which they were asked to use a slider ranging from “Not sure at all” to “Definitely sure” to indicate how confident they were in their answer. There were 20 estimation trials (five for each distance). The motivation for this phase of the experiment was to relate the variation we expected to observe in participants’ choices with how well they understood the performance range of the truck/square. We present these correlations (below) and for completeness, summary plots are presented in section A of the Online Supplementary Material.

At the end of the experiment, participants were shown a screen that indicated how successful they were overall. They were then asked to fill out a short questionnaire to indicate three outside hobbies and answer one of three randomly selected questions from the Cognitive Reflection Task (CRT, Frederick, 2005). These were included to explore individual differences in choice, but because variability was more restricted than expected, this was not included in the analysis.

### Analysis and results

Binomial regression models were fitted to the learning phase success rate data to check that the optimal strategy in the decision phase remained the same across the conditions and different screen resolutions for all the experiments in this series. This check indicated that the conditions and screen resolution groups sometimes led to different success rates for the two close-middle distances. This means that whether participants should have been focusing or dividing is inconsistent at the two middle distances. To simplify the analysis across this and the rest of the experiments, we therefore excluded the inner two distances and compared choices only between the closest and farthest distances (for these two distances, to maximise success participants should have placed the avatar in the middle when the targets were at their closest, and adjacent to one of them at their furthest). Restricting the analysis to these two conditions ensured the choice designated as optimal consistently leads to a higher probability of success than any other target placement, irrespective of any other manipulations of variability or agency in the experiment. The full learning phase results can be found in Section A of the OSM. The success rates align closely with the decisions, so to limit repetition these are also reported in the OSM. In this and all the analyses reported here, we used a Bayesian approach, because our main interest is in estimating the distribution of placement decisions participants tend to make and how that changes under different conditions. We therefore express our results in terms of the 95% Highest Density Interval (HDI), which is the range of values that contain 95% of the samples from the posterior. Often this takes the form of a distribution of differences between the predicted means of two conditions, and when this distribution of differences does not include 0, this roughly aligns with the standard of evidence usually applied in null-hypothesis significance testing to reject the hypothesis that the distributions were drawn from the same population (with a type 1 error of 0.05), but the focus in Bayesian analysis is on the distribution and not the inference (McElreath, 2020). The data are available on the open science framework to anyone who wishes to apply a different analytical approach.

#### Decision phase

**Fig. 2:**
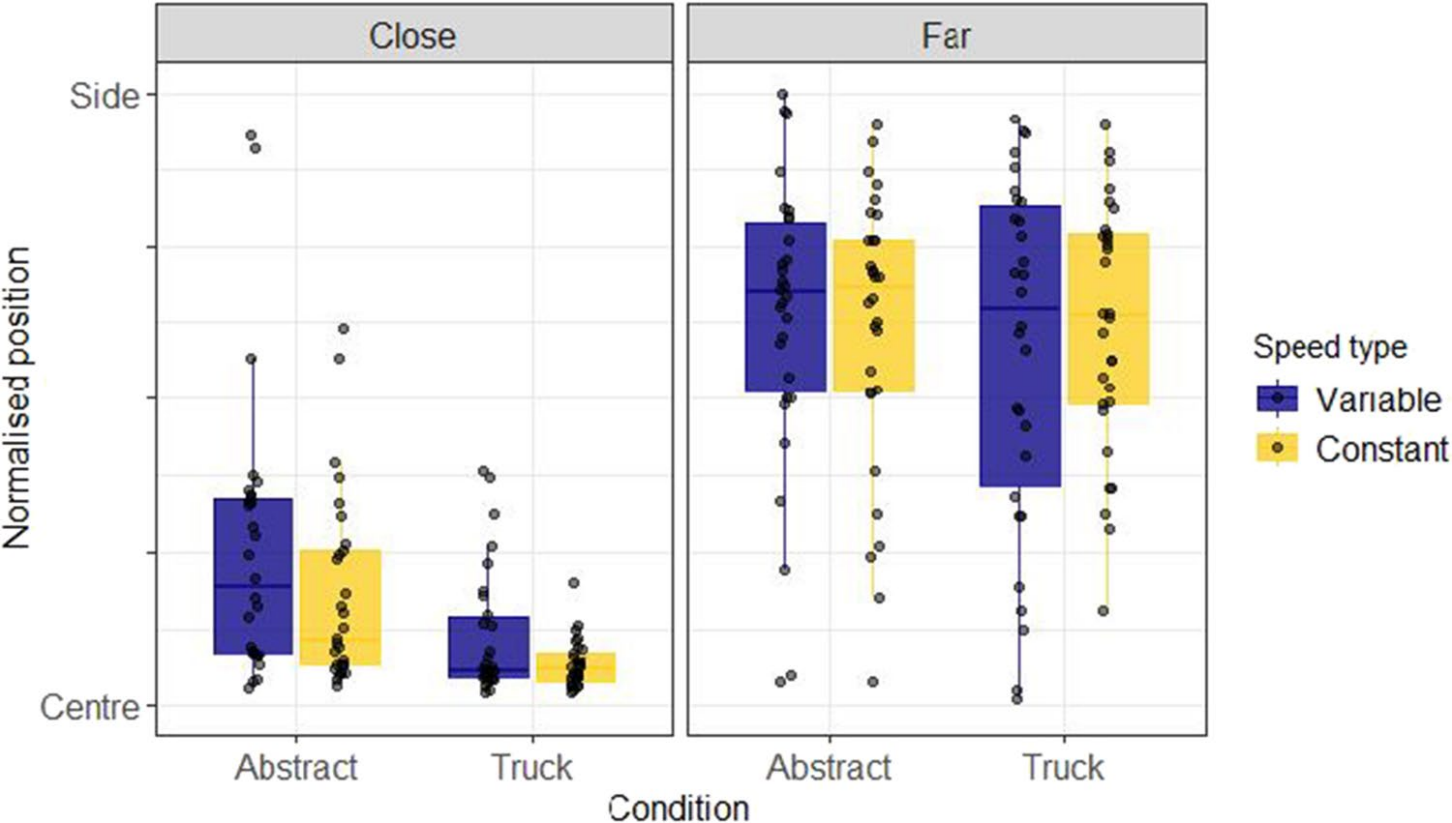
Normalised truck positions chosen by participants in Experiment 1. These boxplots summarise the normalised truck positions chosen by each participant in the Truck and Abstract framing conditions, coloured separately by Speed type (Constant or Variable). The y axis indicates the absolute normalised position of the avatar on the screen ranging between the centre of the screen and the position of either of the two possible targets. The box-plots are split to show the distribution of placement positions for the close and far distances, with dots for individual participant means. Facets by participant can be seen in section A of the Online Supplementary Material

Figure 2 shows the absolute normalised position of the avatar for close and far conditions. This was calculated by dividing the absolute position chosen by participants by the distance of the target from the centre of the screen on that trial. From this it is clear that participants were better able to "solve" this focus-divide dilemma than has been observed in previous versions (e.g., Clarke & Hunt, 2016). Most participants appeared to take into consideration the distance between the targets and made choices that were in line with the optimal strategy, as seen from the large difference in selected positions between close and far distance conditions.

**Fig. 3:**
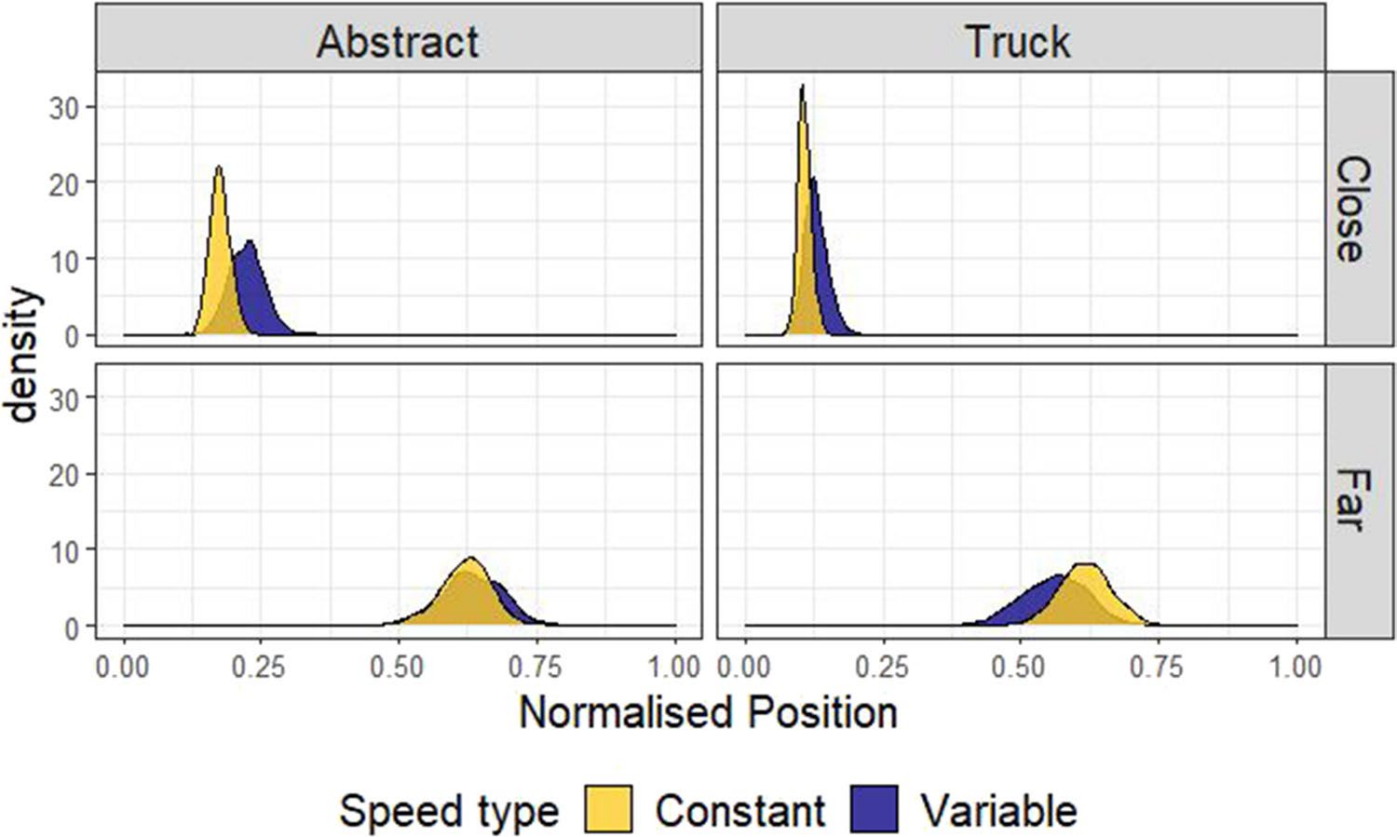
Model predictions for Experiment 1. These plots show the predictions of the Bayesian beta regression model fit using the decision data of Experiment 1. The facets show model predicted means conditioned on the data. The prior predictions and random effects structure is presented in section A of the Online Supplementary Material. The same prior was used for all subsequent analyses

A Bayesian beta regression using the brms package (Bürkner, 2017, 2018) in R (R Core Team, 2021) was used to model placement of the Avatar across conditions. The value being estimated was the normalised position (f) of the Avatar, with 0 corresponding to the avatar placed in the centre, and 1 corresponding to the Avatar placed next to one of the targets. Placement position (f) was scaled so that 0 < f < 1 and compressed by a small amount (1e-4) to fit a beta distribution. Any points that were above 1.1 (i.e., the avatar had been placed further from the centre of the screen than either of the two buildings) were excluded for this analysis resulting in a loss of 0.22% of the total data. Three categorical predictors were entered into the model: (1) Distance (Close or Far), (2) Speed (Constant or Variable) and (3) Framing (Abstract or Truck). All these variables were allowed to interact in the model, with Distance and Speed interacting in the random effects structure by participant. The model was specified as follows: Normalised position ~ 0 + Distance * Speedtype * Framing + (0 + Distance * Speedtype | Participant). The priors for this model were defined by a normal distribution with parameters set to be weakly informative (see Fig. 3). For this and the later experiments, comparisons between conditions were made in terms of posterior predicted means from the fixed effects of the model, and 95% highest density intervals (HDIs) were used as summaries. The choice of 95% for the interval is somewhat arbitrary (McElreath, 2020), as the Bayesian analysis we present here is intended to estimate effect size, not test significance. Figure 3 shows the results of the model, which confirmed the effect of distance. The mean difference in terms of normalised avatar placement (Far-Close) is 0.36 (95% HDI |0.32, 0.44|).

#### Adjustment measure

**Fig. 4:**
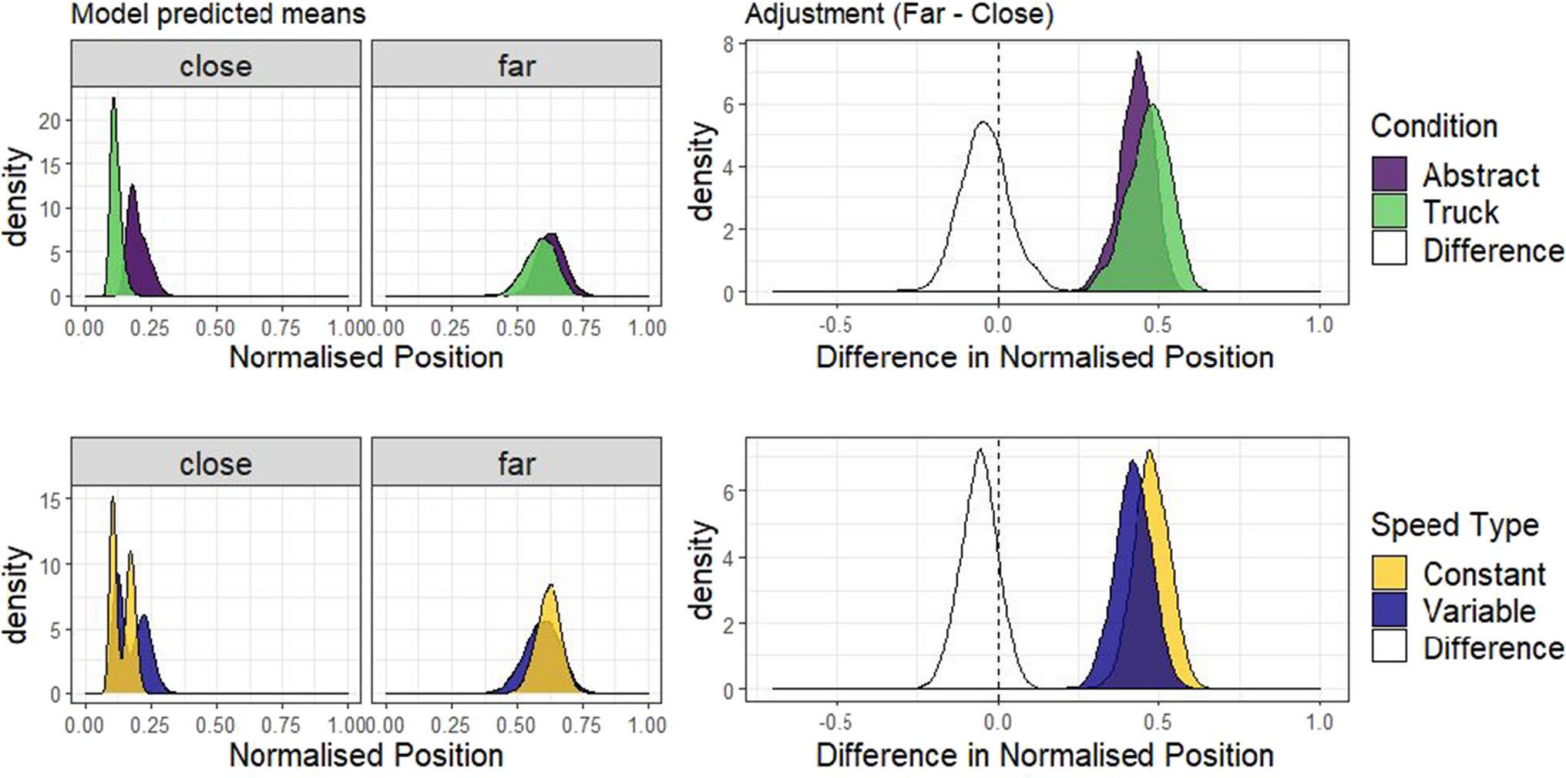
Differences in adjustment of position by Speed type and Framing condition. Left side: the posterior predicted means of the model by distance, separately for each framing (Truck or Abstract) and Speed type (Constant or Variable). Right side: the distributions of the adjustment effect (the difference between the Far and Close conditions, calculated by resampling from the distributions in the left column). The white distributions show the difference in adjustment of position with distance between Abstract and Truck (top) and Constant and Variable (bottom) conditions

To express the adherence to an optimal strategy in a single distribution, we sampled from the far and close posterior distributions of truck placements and produced a distribution of differences shown in Fig. 4. These differences, which we call *adjustments*, will approach 1 the closer choices get to an optimal strategy. Adjustments were compared for each level of framing (Abstract-Truck, shown in the teal and purple distributions in Fig. 4, respectively). As can be seen from this plot, the distributions for framing overlap almost perfectly. The white distribution to the left shows an estimate of the size of the difference between these two distributions, by creating a new distribution of differences by sampling from the Truck and Abstract adjustment differences. This white distribution represents the difference in adherence to the optimal strategy for trucks versus abstract squares and it clearly centres on 0, suggesting participants’ adjustments of the avatar’s position with distance were similar for Abstract versus Truck conditions (mean difference = -0.05, 95% HDI | -0.20, 0.09|). The results suggest that if a difference does exist between the Truck and the Abstract condition, it is too small to explain why participants perform better at the fire trucks task than previous versions of the focus-divide dilemma.

The blue (darker) and yellow distributions in Fig. 4 compare how much participants adjusted placement of the avatar with distance, but compare speed conditions (Variable-Constant), which was manipulated within-subjects and between blocks. Here, the Constant condition led to adjustments in the position of the avatar that were more optimal (closer to 1) than the Variable condition, although the distributions also overlap, suggesting no difference is also plausible (mean difference = -0.06, 95% HDI | -0.16, 0.06|, see the white difference distribution). As with framing, the difference here is not large enough to suggest that uncertainty can explain why people made suboptimal choices in previous versions of the focus-divide dilemma. However, some tentative support for the hypothesis that certainty facilitates optimal choices has been provided by these data, and we will return to this at the end of Experiment 2, where we address order effects across both Experiment 1 and Experiment 2 together.

### Discussion

We developed a computerised version of the focus-divide dilemma with the goal of testing larger groups of participants and exploring individual differences in choice behaviour. While there were still participants that opted for central positions irrespective of the task difficulty, it was more common to observe participants that altered their strategy with distance. That is, the majority of participants adopted a strategy that approached the optimal solution, unlike all other versions of this task except *reaching* (Clarke & Hunt, 2016). Although this means that the fire trucks task is not suitable for investigation of individual differences underlying the failure of intuition observed in previous work (because it did not produce the same failure), these findings instead present an opportunity to explore the factors that elicit better decisions. In Experiment 1 we ruled out task framing as being one obvious reason for the difference; considering participants made similarly optimal choices when placing coloured squares as for fire trucks, an explanation based solely around participants wanting to save houses from burning down is not tenable. We cannot rule out that participants in the coloured square condition achieved similar performance to the fire trucks by applying their own framing or story to the events of the trial (in the sense demonstrated in the classic studies by Heider & Simmel, 1944). If they did do so in this condition, it would be all the more surprising that they seemingly fail to come up with framing that helps them perform better in the previous versions of the task. There were small differences related to the speed condition, with participants being more optimal when the avatar moved at a constant speed compared to variable speed (these are returned to at the end of Experiment 2). This difference is not sufficiently large to explain why participants are altogether better in their choices in this computerised version of the task as they were in previous versions of the dilemma, so other reasons need to be considered.

One important difference in the current task relative to previous ones is the elements of the task under participant control. In previous versions, participants controlled both task execution and strategy, for example, they threw beanbags at targets, and also decided where to stand. In the fire trucks task, participants only controlled strategy. They decided where to position the truck, but had no control over the execution of the subsequent task; the truck automatically drove towards the burning house. The results of a study examining the effect of motivation (James et al., 2019) indirectly suggest task execution may be important: Participants with extra motivation to improve task performance did so by improving execution, while their strategy remained the same. They concluded that participants were ‘working harder, not smarter’, improving their performance by minimising response errors and focusing their attention more effortfully. By extension, the fire trucks task removes the task execution component altogether; participants had nothing else to focus their efforts on aside from the placement of the truck, and the only way they could improve their chances of success was through making better placement decisions.

## Experiment 2: Testing agency with simple driving

The hypothesis we tested in this experiment was that having more control over task execution distracts participants from the strategic aspects of the task. We manipulated participants’ control over the execution element of the fire trucks task by requiring them to “drive” the truck towards the fire, predicting that this added control would make decisions around strategy less optimal relative to Experiment 1. In addition, we try to replicate the small effect of truck speed observed in Experiment 1 to disambiguate whether certainty does or does not promote optimal decisions in this task.

### Methods

#### Participants

Thirty participants (18 female) between the ages of 18 and 26 years (M = 21.5, SD = 1.7 years) took part in this experiment. Participants were recruited via the SONA system at the University of Aberdeen in exchange for course credit. The truck condition from Experiment 1 (N = 30) was used as a comparison for this experiment as the experiments were otherwise matched in terms of their design. These data are labelled as the *Automatic* group below. The group that had to perform the task were labelled as the *Manual* group as they were now in direct control of the avatar’s performance.

#### Justification of sample size

Based on the data from Experiment 1, a power analysis similar to that of James et al. (2022) was applied to justify the sample sizes used in Experiments 2 and 3. We used bootstrapping methods to simulate the hypothesis that agency over performance would shift decisions away from optimal. Data from the *throwing* task (Clarke & Hunt, 2016) was used to simulate the effect of adding control over the truck’s motion, as in the throwing task participants controlled both the strategic decision about where to stand and the subsequent performance element of the task. Data from the truck condition of Experiment 1 was used as a comparison. Uncertainty around the difference in the adjustment effect plateaued around N = 18. Based on this analysis, we can say that our conclusions in Experiments 2 and 3 are unlikely to change with a larger sample size, as our sample sizes per condition were greater than N = 18 in all cases. The power analysis is reported in OSM section B.

#### Procedure

The procedure was the same as Experiment 1 aside from the details described below. All participants completed the experiment on the same computer as the solo participants in Experiment 1 in a room on their own.

##### Learning phase

This phase differed in that participants were now in control of how quickly the fire truck would respond. Participants were shown one house and the fire truck. Their instructions were to drive the fire truck towards the house once it had caught fire by pressing either the left or right arrow keys to move in the corresponding direction. If the participants responded too early (before the fire started) or too slowly (after the house had burned down), the trial was repeated with a message alerting them they had either been too quick or slow. There was a set time limit for participants to be able to reach the target, as was the case in Experiment 1, however, the time was augmented slightly (by 22 frames/0.36 s) in order to adjust for reaction time, and give participants in this Manual version of the task a similar chance of success to the participants in the Automatic version. This meant that participants had ~2 s to reach the target on each trial in the Manual version while those in the Automatic version had ~1.6 s. Similar to Experiment 1, each of the four distances were repeated ten times in a row to provide a total of 40 trials for each block. If participants responded too quickly or not at all, participants were told that they had responded too quickly/slowly and that this trial was to be repeated.

##### Decision phase

As in Experiment 1, participants placed the truck between the two houses by pointing and clicking using the mouse and then pressing the spacebar to confirm their choice. Participants were told that on every trial, each house had an equal chance of catching fire, and that their job was to put out as many fires as possible by placing the truck somewhere between the two houses and then driving it towards the burning house. As in the Learning phase, participants had to press the left or right arrow key to drive the truck towards the burning house, and if participants responded too quickly or not at all, the trial was repeated by adding it to the end of the block.

##### Estimation phase

As in Experiment 1, participants would see the fire truck and one house separated by one of the four distances used in the Learning and Decision phases. They responded to the question “Will the Fire truck reach its target?” by pressing the “Y” or “N” key. They were then asked to indicate how sure they were of their previous answer by marking a location on a Visual Analogue Scale that ran from “Not sure at all” to “Definitely sure”.

### Analysis and results

**Fig. 5:**
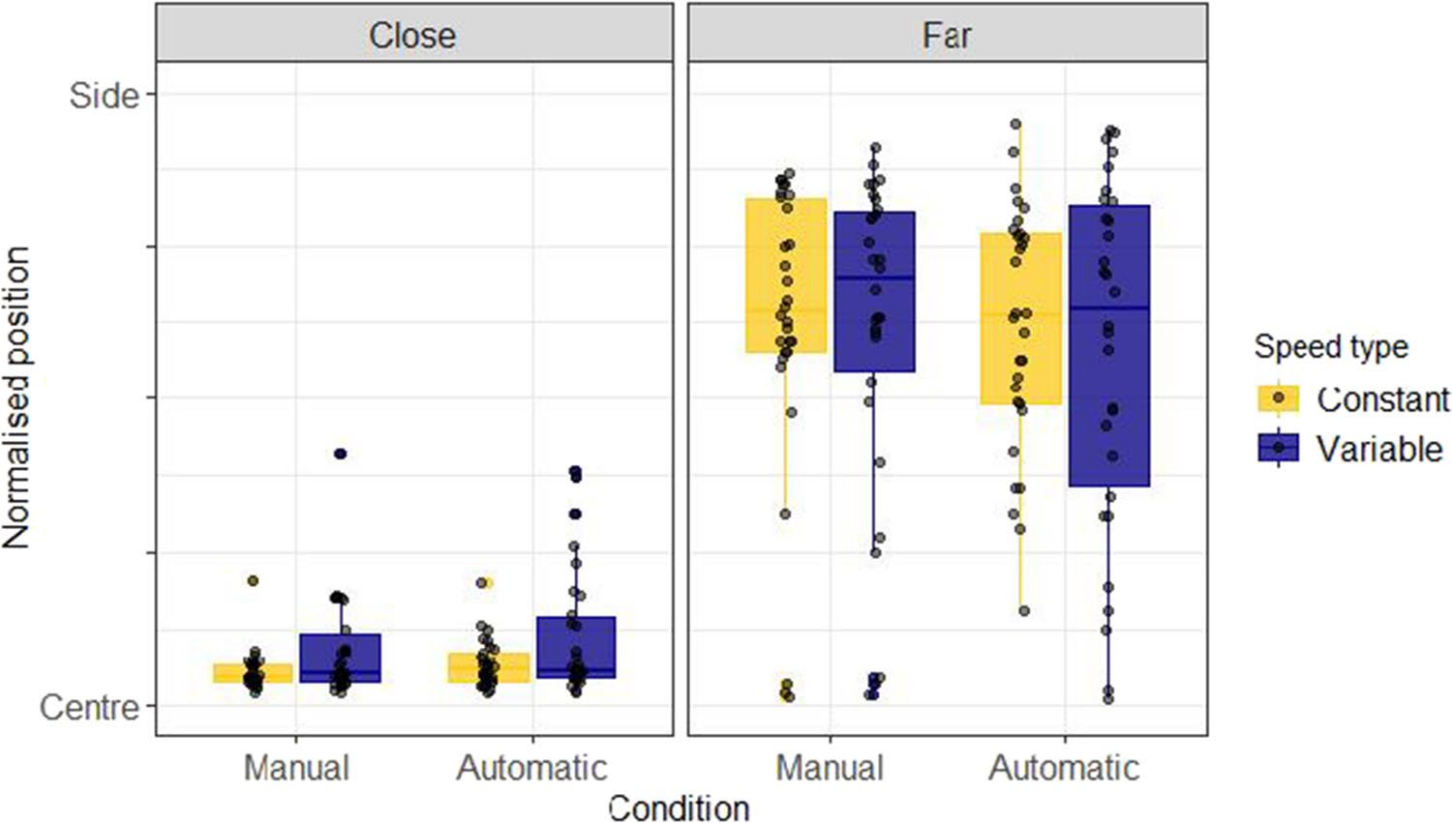
Normalised truck positions chosen by participants in Experiment 2. These boxplots summarise the absolute normalised truck positions chosen by each participant in the Automatic (Experiment 1) and Manual (Experiment 2) conditions, coloured separately by Speed type (Constant or Variable), ranging between the centre of the screen and the position of either of the two possible targets. The box-plots are split to show the distribution of placement positions for the close and far distances, with dots for individual participant means

**Fig. 6:**
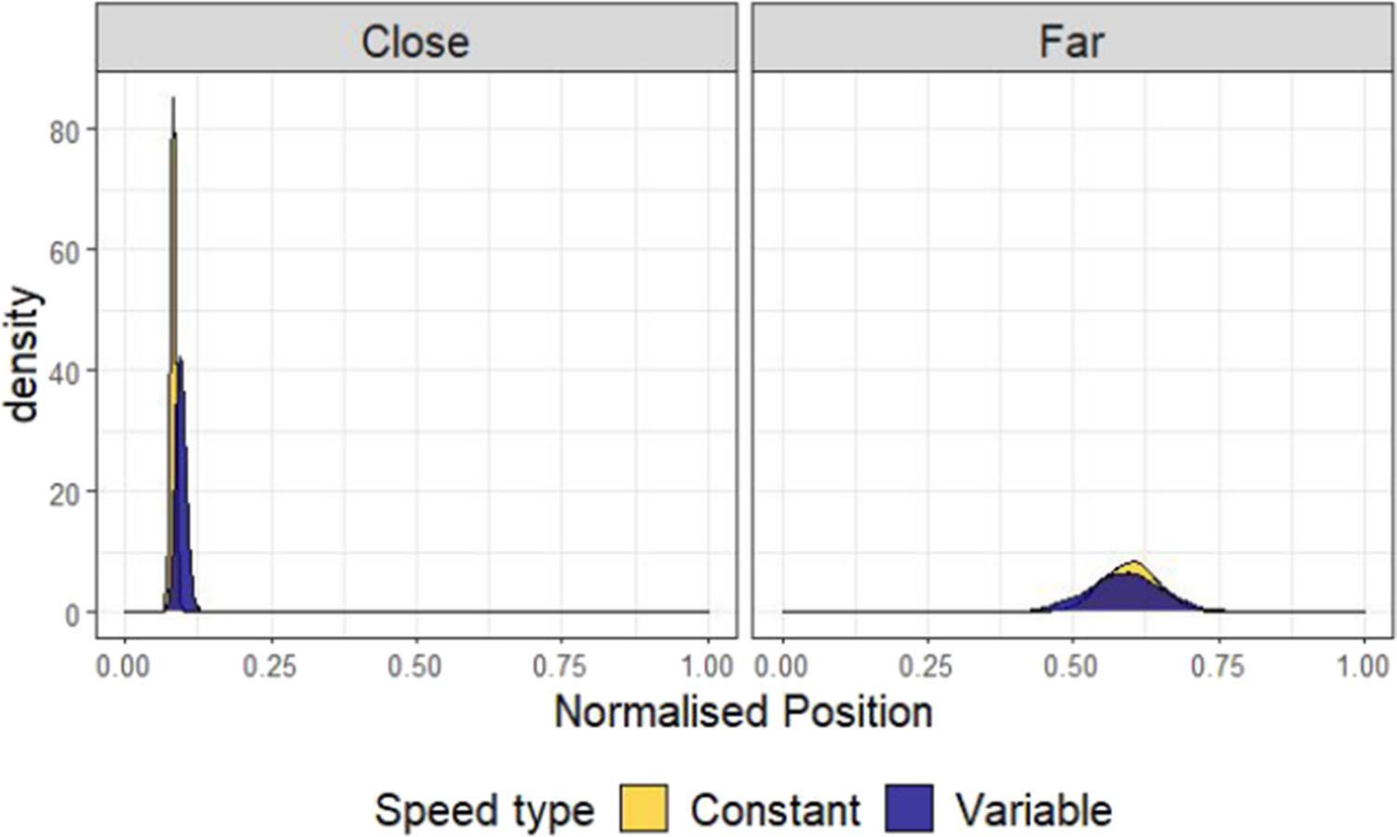
Model predictions for Experiment 2. These plots show the predicted means for the Manual condition of the Bayesian beta regression model fit using the decision data of Experiment 2 and the truck condition of Experiment 1. The priors for this model were the same as in Experiment 1. The priors and the random effects structure are presented in the OSM (sections A and B, respectively)

Removing trials with placements outside the range resulted in a loss of 0.43% of the data. Matching Experiment 1, only the closest and furthest distances were compared from the decision phase (full results are presented in OSM Section B). The model was specified as before, but agency condition (Manual or Automatic) replaced framing as a between-subjects predictor. The model was specified as follows: Normalised position ~ 0 + Distance * Speedtype * Agency + (0 + Distance * Speedtype | Participant). Distance and Speed type were allowed to interact in the random effects structure by participants. The priors for this model were defined in the same way as Experiment 1 (see OSM section A for a plot of the prior distribution). The empirical data is shown in Fig. 5 and Fig. 6 shows the results of the modelling procedure. Both demonstrate a clear effect of distance: Participants placed the truck further from the centre at the furthest distance as compared to the closest distance (mean difference = 0.39, 95% HDI |0.25 , 0.47|), replicating the finding from Experiment 1.

#### Adjustment measure

**Fig. 7:**
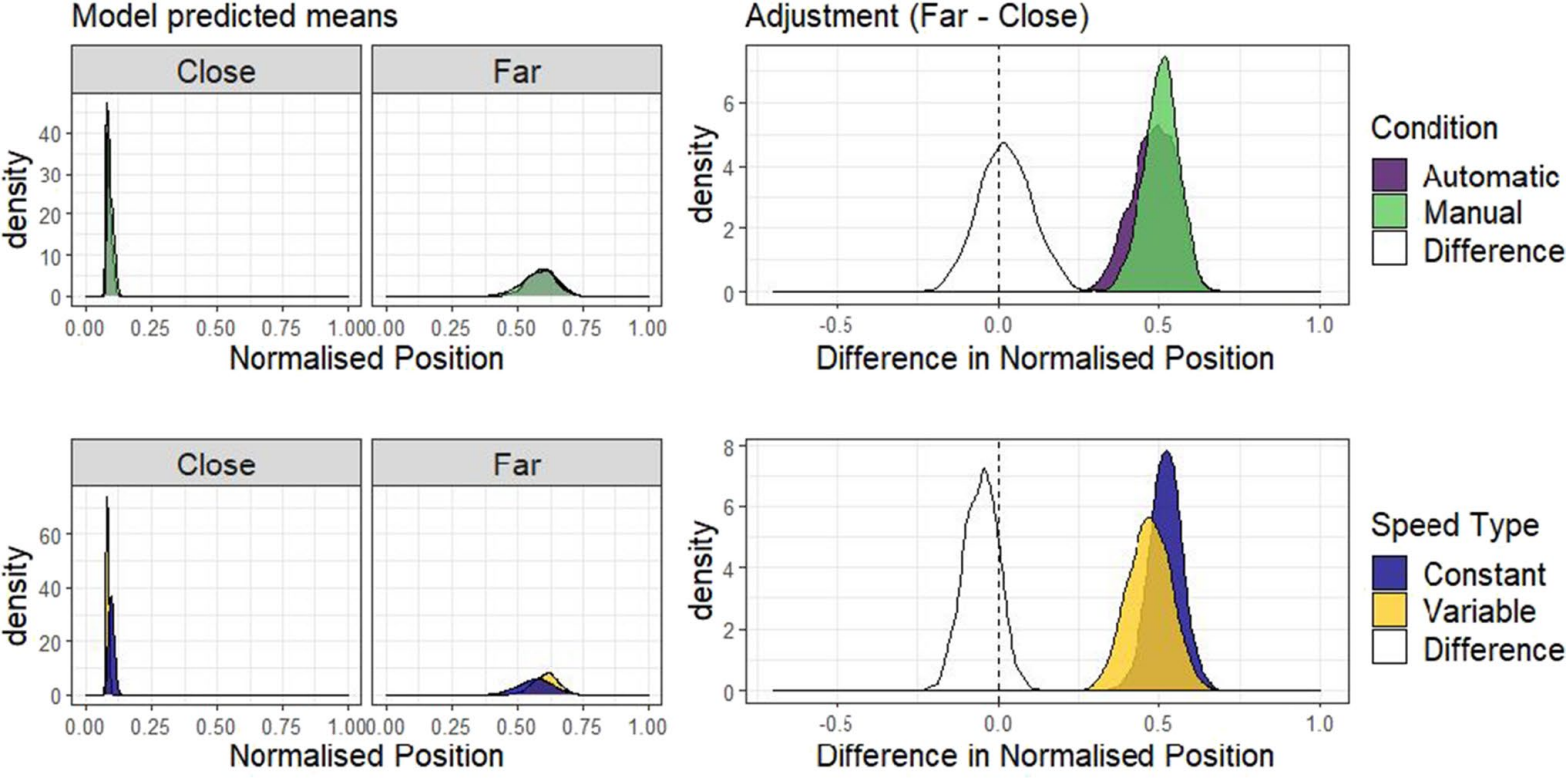
Differences in adjustment of position by Speed type and Agency condition. The plots in the top row show the posterior predictions of the model by distance, separately for each level of agency condition (Manual or Automatic). The right plot shows the distribution of differences between close and far, based on sampling from the posterior distributions shown on the left. The white distribution on this plot is the distribution of differences between the Manual and Automatic adjustment size, and the fact that it is centred on 0 reflects the overlap in these distributions. Note that the Automatic condition in this plot is based on the data from the truck condition in Experiment 1. The bottom row shows the same analysis but with uncertainty (Variable or Constant) as a predictor, and includes data from both Experiment 1 (Truck condition) and Experiment 2

As for Experiment 1, the adjustment with distance (Far-Close) was compared for each level of Agency (Manual-Automatic, between-subjects) or Speed type (Variable-Constant, blocked within-subjects) to assess whether a difference in adjustment was present for either variable. Agency did not appear to move decisions away from optimal, as the distributions for Manual and Automatic closely overlap (top row, Fig. 7). A distribution of the difference between Manual and Automatic adjustments is shown in white in that plot, and has a mean of -0.02, 95% HDI |-0.16, 0.10|). Similar to Experiment 1, a larger difference in adjustment was observed as a function of the Speed type condition, with the Variable condition leading to adjustment in position that varied less with distance than the Constant condition (mean difference = -0.05, 95% HDI |-0.16, 0.02|).

#### Order effects

We conducted an analysis of Experiments 1 and 2 together to see if the within-subjects effect (i.e., Constant vs. Variable truck speed) depended on the order in which participants viewed each condition, and whether the main findings still hold between-subjects when considering only the first block. Models were re-fit as described in the analysis sections of each experiment, with the addition of Block (1 or 2) interacting with the other predictors as a fixed effect and in the random effects structure. Model predictions indicated the effect of Distance is robust to order effects in each experiment, as well as the adjustment effects by the between-subject effects of Framing and Agency (see OSM section B).

To investigate order effects in Speed type, a single model was fit across the data from Experiments 1 and 2. We deemed this appropriate because Experiment 2 was designed as an extension to directly compare with the Truck condition of Experiment 1, and because this affords greater power with which to estimate the size of any order effects. The model was specified as before and fit to the combined data set (n = 90), including Distance, Speed type, and Block, which all interacted by participants in the random effects structure. The model was specified as follows: Normalised position ~ 0 + Distance * Speedtype * Block + (0 + Distance * Speedtype * Block | Participant). Before fitting the model, 0.13% of the trials were excluded for falling outside of the normalised range between 0 and 1. Model predictions for the adjustment effect are shown below.

**Fig. 8:**
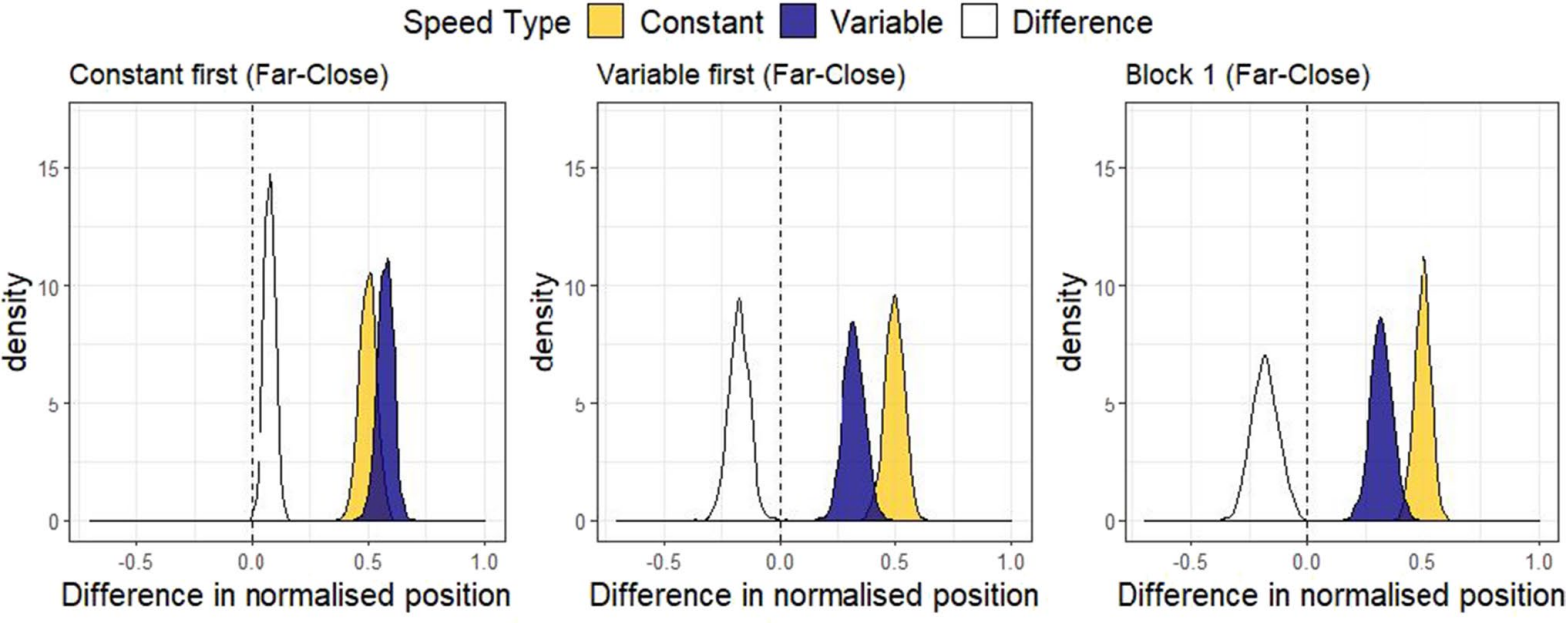
Order effects model predictions. The posterior predicted adjustment effect (the difference between the Far and Close conditions), separately by Constant or Variable first (left and middle), and only considering Block 1 (right), to demonstrate the influence of Block order on within- and between-subjects effects

The left and middle plots of Fig. 8 demonstrate that order influenced the within-subjects effect of Speed type. When the Variable condition came first, participants adjusted position more in the subsequent Constant block (mean difference = -0.18, 95% HDI |-0.27, -0.09|). When the Constant condition came first, participants adjusted position more in the subsequent Variable block (mean difference = 0.07, 95% HDI |0.02, 0.12|). To check whether our conclusions hold considering only the first condition encountered by participants, we examined model predictions for just Block 1, shown on the right in Fig. 8. Here it is clear that participants adjust position more for Constant than Variable speed (mean difference = -0.18, 95% HDI |-0.30, -0.07|).

### Discussion

Participants were close to the optimal solution even when they controlled the truck moving to the house. Although this does not support our hypothesis that agency influences strategic decisions, the “control” of the truck was relatively minimal, amounting only to pressing the appropriate key. The ways in which participants could improve their task execution (and therefore their chances of successfully reaching the house in time) were limited in comparison to previous tasks, such as *throwing* (Clarke & Hunt, 2016). Another limitation to Experiment 2 was the agency conditions are compared across different experimental settings, meaning we cannot rule out the possibility that an agency effect was present but masked by differences between the groups of participants tested. This weakness is addressed in Experiment 3, in which the agency manipulation was not associated with any systematic difference in testing conditions.

The order effect analysis can be interpreted as follows: participant strategies are improved when truck speed is constant compared to when it is variable, and the difference in adjustment depending on condition order indicates experience with the Constant condition improves subsequent performance in the Variable condition. It should be noted that a limitation of our design is that we cannot determine how much of the apparent carry-over is actually a learning effect. This could be resolved by controlling for practice (Constant-Variable, Variable-Constant, Variable-Variable, Constant-Constant). A carry-over effect is reminiscent of order effects observed by Cox and Griggs (1982), who asked participants to solve deductive reasoning problems under more familiar (drinking age problem) and less familiar (shirt colour problem) but otherwise similar conditions; they observed that participants who solved the drinking age problem first were more likely to correctly solve the less familiar version of the same problem than those who completed the problems in the opposite order. We did not observe familiarity/framing effects at all in our experiments, but we do see a benefit associated with certainty around truck speed, and a carry-over of this benefit when the truck speed then becomes variable. An important point here is that by collapsing across counterbalanced Speed type conditions in the analyses above, we underestimated the Speed type effect in Experiments 1 and 2 that is evident when order effects are accounted for.

## Experiment 3: Testing agency with more complex driving

Here we increased the difficulty of task execution by requiring participants to perform a harder version of the driving task. It was hypothesised that increasing the difficulty of the task execution would cause participants to focus more on their performance in terms of task execution, rather than the decision aspect of the task, leading to choices that more closely resemble those of participants in the *throwing* or *memory* tasks (Clarke & Hunt, 2016). To increase the difficulty, participants in this version drove the truck in an ‘L’ shaped path, first driving downwards and then switching direction towards the target, whereas in the first two experiments they had only to drive left or right with no switch in direction. We also manipulated speed conditions (Variable and Constant) as a between-participants factor, to confirm the growing evidence from Experiments 1 and 2 that predictable outcomes promote optimal performance.

### Method

#### Participants

A total of 126 participants took part in Experiment 3. Participants (n = 71; 37 male; mean age = 24.4 years, SD = 6.9) in the Constant truck speed group were recruited via Prolific Academic (https://www.prolific.co/, accessed August 2020), and of those in the Variable speed group (n = 55; 31 female; mean age = 24.1 years, SD = 6.4), 16 were recruited through SONA, and the remaining 39 out of 55 were recruited through Prolific Academic. The Variable and Constant groups were run in separate waves of testing. All participants were reimbursed £2 or with course credit (where applicable) for their time. The larger sample reflects the fact that both the agency and speed conditions were manipulated between groups in this experiment, to keep the duration of the experiment under 15 minutes for internet-based participation. Participants were required to complete the experiment using a desktop or laptop as the task required keyboard input, and their display needed a resolution of at least 1,024 × 768. Device type was recorded to ensure that they had seen the correct stimulus presentation and excluded those that did not meet the criteria. This resulted in eight exclusions. The final breakdown of the sample over groups was: 41 Manual/Constant, 30 Automatic/Constant, 32 Manual/Variable and 23 Automatic/Variable.

#### Procedure

This version of the task was deployed online. The procedure was the same as the previous two experiments apart from the following. The task was programmed in Psychopy v3.1.2 (Peirce et al., 2019) and hosted on www.pavlovia.org. Because the experiment was conducted online, additional instructions and guidance were added to all aspects of the task to ensure it could be completed independently. In order to be classified as a successful trial, the centre of the truck had to be within a radius of 45 pixels from the centre of the target house before it had burnt down. Agency and Speed type were manipulated between subjects, such that within the Constant and Variable motion groups, half of the participants had control over the motion of the truck, and the other half observed the truck moving automatically to the target.

##### Learning phase

There were four distances at which participants were tested: 200, 300, 400, and 500 pixels. The values corresponded to the sum of the vertical and horizontal distance from the centre of the truck to the centre of the target house. On each trial, the truck was displaced by 100 pixels vertically and set in the middle of the screen horizontally. The house would be positioned either to the left or the right of the centre at random by the distance set for that trial. Each of these distances was tested 12 times giving a total of 48 trials for this phase. Participants used the mouse to press a small button in the bottom half of the screen to initiate each trial. This button also served the purpose of positioning the participant’s mouse cursor close to the controls for the truck. This ensured that participants were all starting from a similar location relative to the controls. In the Manual condition, if participants had clicked the button prior to the house starting to burn, they were informed they had responded too quickly and the timer was reset. In both conditions the house burned for a total of 2 s.

**Fig. 9:**
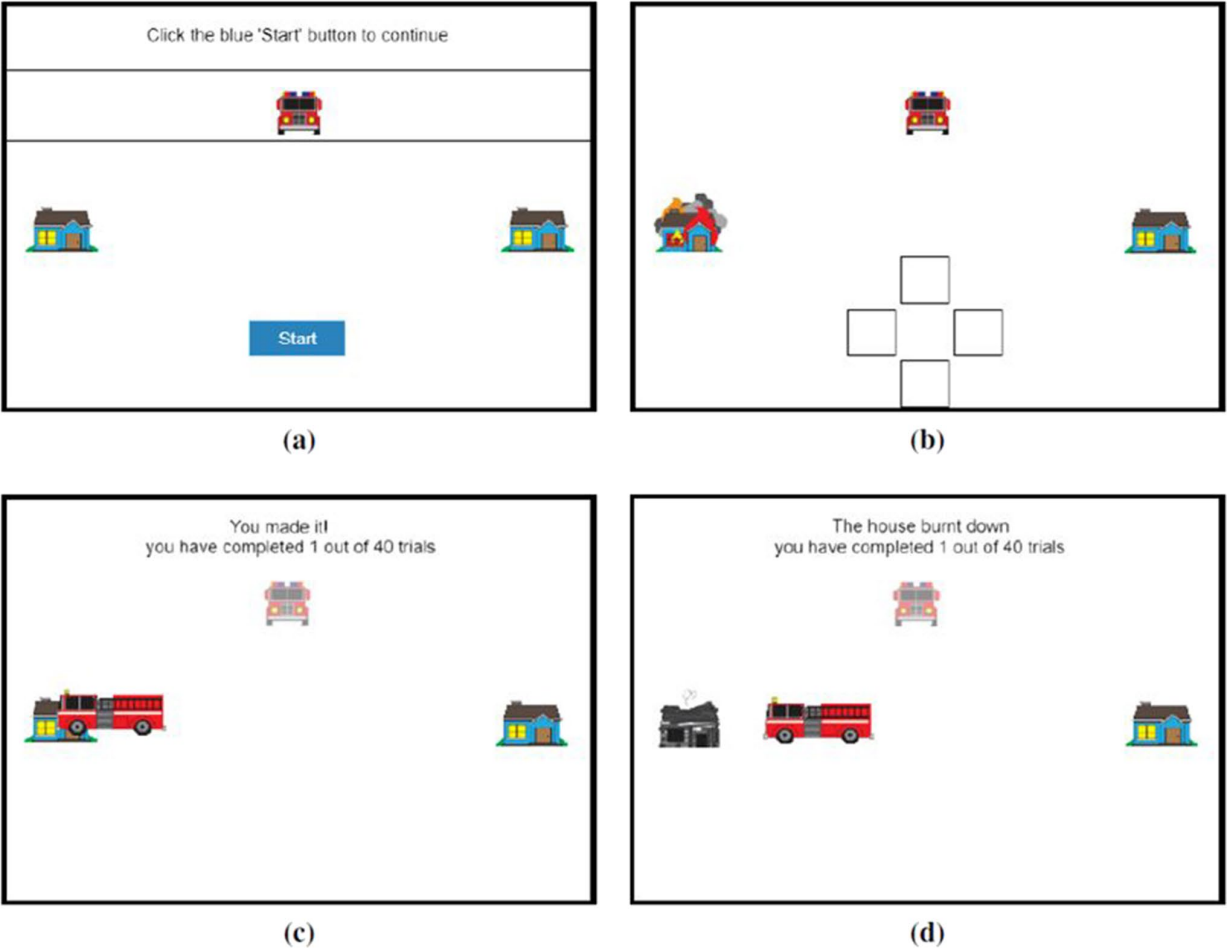
Example displays in the Decision phase of Experiment 3. This figure shows each part of a trial in the Decision phase of the experiment. (**a**) shows the initial screen, (**b**) shows the screen once the target house had begun to burn, (**c**) and (**d**) show the outcome if participants had succeeded or failed in reaching the house in the time allowed

In the Manual condition participants controlled the truck’s motion by clicking on one of four boxes arranged at the bottom of the screen in a cross shape (as in Fig. 9, or see OSM section C), corresponding to up, down, left and right. Participants in the Manual condition started the learning phase with instructions for how to use these controls, as well as the truck on screen which they could interact with for as long as they wanted. Participants were told to press the spacebar when ready to move on to the experiment. In the Automatic condition, the truck’s motion was defined on each trial using three parameters: response time (i.e., how long it took the truck to start driving), the precision of its movement (i.e., how far the truck would travel in a downwards direction before turning towards the target), and how long it took to switch from travelling in one direction to another (e.g., the time from releasing the downwards button to clicking on the rightwards button). Each of these parameters was sampled at random from distributions fit to pilot data (from one of the authors and a Masters student performance) in order to recreate human performance in this task. The intention was to match the performance of participants in the Manual condition with the behaviour of the truck in the Automatic condition. The truck could only move in cardinal directions, not diagonally.

##### Decision phase

Participants placed the truck somewhere between two black lines that were positioned 100 pixels above the centre of the screen (see Fig. 9) by pointing and clicking using the mouse. At the start of each trial the truck’s starting position was 200 pixels above the centre and within the middle third of the distance between the two houses. One house appeared to the left of the centre at the distance for that trial, and the other to the right (distances were 200, 300, 400, or 500 pixels). Once the house caught fire, the deadline for the truck to reach it was 2 s, as in the learning phase.

##### Estimation phase

On each trial, participants would see the truck 200 pixels above screen centre, and one house to the left or right on the horizontal meridian. Participants responded to the prompt “Could the fire truck reach this building?” by clicking along a (continuous) black line to indicate their belief that the truck would “Definitely” or “Definitely Not” reach the house in time. The four distances (200, 300, 400, 500) were repeated twice for a total of eight trials. The OSM section C shows the display of this phase of the experiment.

### Analysis and results

#### Decision phase

**Fig. 10:**
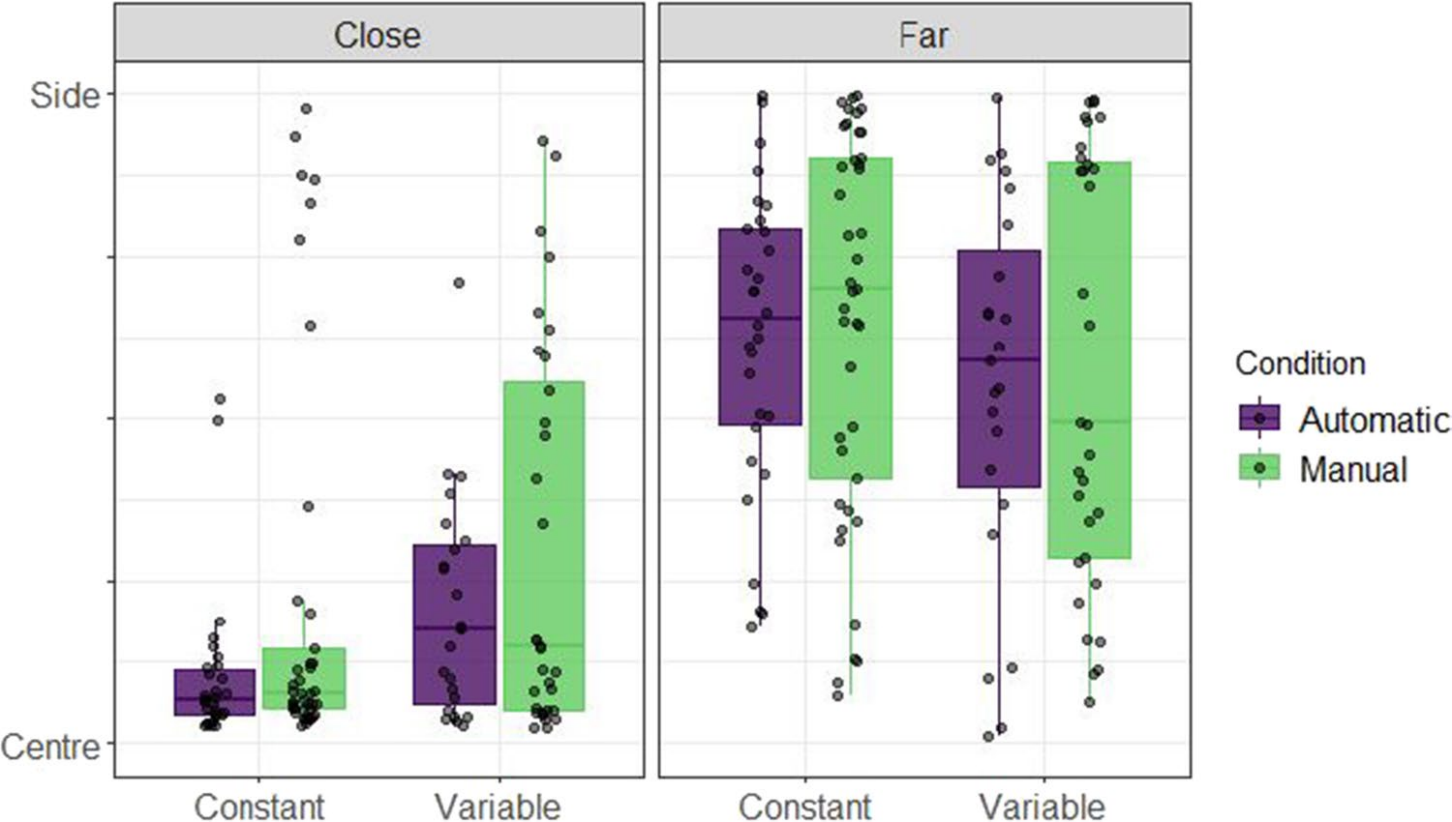
Normalised truck positions chosen by participants in Experiment 3. These boxplots summarise the absolute normalised truck positions chosen by each participant in the Constant and Variable conditions ranging between the centre of the screen and the position of either of the two possible targets, coloured separately by agency condition (Automatic or Manual). The box-plots are split to show the distribution of placement positions for the close and far distances, with dots for individual participant means. Facets by participant can be seen in section C of the Online Supplementary Material

**Fig. 11:**
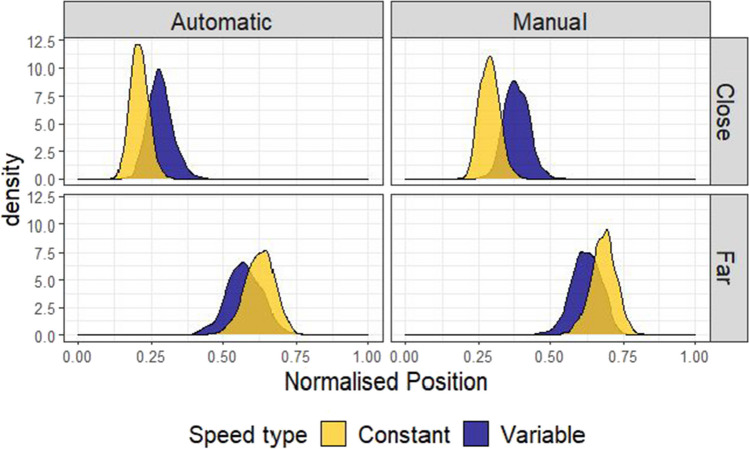
Model predictions for Experiment 3. These plots show the predicted means of the Bayesian beta regression model fit using the decision data of Experiment 3. The priors for this model were the same as the previous two experiments, and can be seen in section A of the Online Supplementary Material. The random effects structure is presented in section C of the OSM

A Bayesian beta regression (Fig. 11) was fit to the decision phase data (Fig. 10). Pre-processing resulted in a loss of 1.01% of the data. The model was specified as in Experiment 2, but only distance was entered into the random effects structure for each participant: Normalised position ~ 0 + Distance * Speedtype * Agency + (0 + Distance | Participant). The results of this experiment are consistent with the previous two, in that when the houses were far apart, truck placement was closer to one of the houses than when they were close together, resulting in a mean difference of 0.40 (95% HDI |0.18, 0.48|) in terms of normalised position.

**Fig. 12:**
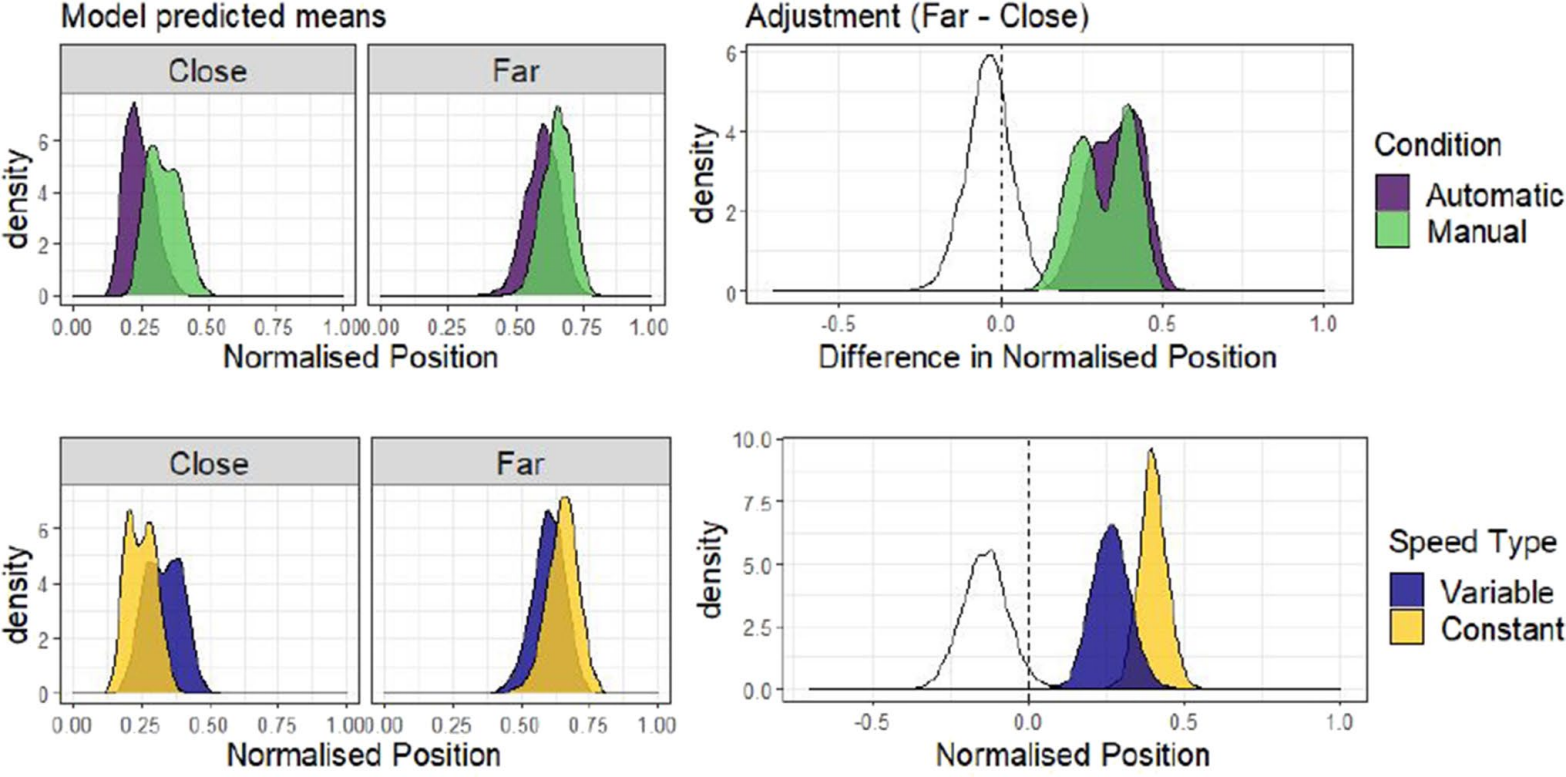
Differences in adjustment of position by Speed type and Agency condition. The plots in the left column show the posterior predicted means of the model by distance, separately for each level of agency condition (Manual or Automatic) and uncertainty (Variable or Constant). The plots in the right column show the adjustment effect (the difference Far-Close), and the difference in adjustment in grey between the levels of each independent variable

### Differences in adjustment

If agency or uncertainty influence strategy, there should be a difference in adjustment of truck position with separation between the levels of each variable (agency condition and speed). The top row of plots in Fig. 12 shows the difference in adjustment depending on agency condition, and it is clear from these, green and purple (darker) plots that the distributions are largely overlapping. Consistent with this, the difference in adjustment between the Manual and Automatic conditions, shown in the white distribution, was centred on 0 (mean difference = -0.043, 95% HDI |-0.19, 0.10|). Although there was no average effect of agency condition, it can be seen in Fig. 12 that the shape of the adjustment distribution in the Manual condition is bimodal, suggesting that there is an effect of agency that might depend on the level of uncertainty, but additional exploratory comparison indicated there was no interaction effect (see OSM section C). This is consistent with Experiment 2, in that having control over the motion of the truck does not appear to move participants away from the optimal strategy, even with a more complex control scheme. In the bottom row of Fig. 12, adjustment is compared across Speed type, and here the Variable truck motion group adjusted position less with distance than the Constant group (mean difference = -0.15, 95% HDI |-0.28 -0.007|). In other words, when the performance of the truck was more predictable, participants were closer to the optimal strategy in that they adjusted position more with distance between target houses.

### Discussion

Experiment 3 tested whether making the task execution element of the fire truck task more demanding would lead to decisions similar to those observed in the *throwing* task (Clarke & Hunt, 2016). Like in Experiment 2, participants were still, on average, more optimal in their decisions than had previously been observed, and more uncertain truck motion resulted in decisions that were further from optimal. The convergence of this latter effect across three separate experiments points to the idea that uncertainty is a key factor that influences decisions in the focus-divide paradigm.

## General discussion

We developed the fire trucks task to explore the cause of previously-observed failures to effectively choose between focusing or dividing resources to achieve goals. The results of Experiment 1 showed that participants effectively adjusted their strategy with the difficulty of the task, in contrast to the persistent failure to do so observed previously (Clarke & Hunt, 2016; Hunt et al., 2019; James et al., 2019; James et al., 2023). We explored three potential explanations for why the fire trucks task produced more effective decisions than other versions of the task: framing, agency, and uncertainty. While manipulations of both framing (Experiment 1) and agency (Experiments 2 and 3) had no clear effect on decisions, across all three experiments, uncertainty about choice outcomes had the small but consistent effect of moving participants away from the optimal strategy. Across all three experiments, participants were more optimal than in all previous versions of the focus-divide dilemma. Even in the most uncertain and distracting conditions, success rate analysis demonstrates that decisions in the task were decidedly better than a simple ‘always middle’ strategy, and approach the results of the optimal focus-divide decision rule (see section C of the OSM).

### Framing

We created an engaging scenario that would be easy for participants to understand. This was important because failures to make optimal choices could reflect not poor strategies, but a misunderstanding of the instructions or a failure of engagement. The generally good placement choices participants made in the fire trucks task suggested the scenario was easily understood by participants, even when deployed online (in Experiment 3). However, houses burning down is arguably a more serious consequence of poor decisions than failing to detect a target or get a beanbag in a hoop. In the first experiment, participants were therefore assigned to either the fire trucks version of the decision problem, or to a version that matched this in all ways but replaced the houses and trucks with coloured squares. The two groups of participants were indistinguishable in their choices of how to place the avatars, suggesting the “story” about preventing houses from burning down was not the main reason participants made better choices. This is an important point because it means the choices participants made in the fire trucks task generalise to other situations, reinforcing its usefulness as a task for studying decision-making. Moreover, the task is well-suited for implementing over the internet because of its intuitive and engaging qualities. For future research, particularly that conducted over the internet, we think the fire truck task could provide a fruitful source of data for exploring choice under uncertainty.

### Agency

We investigated agency because control over task execution was identified as a key difference between the fire trucks task, in which decisions approached optimal, and previous focus-divide dilemmas, in which decisions were poor (Clarke & Hunt, 2016; James et al., 2017; Hunt et al., 2019, James et al., 2019). Based on James et al.'s (2019) results, we reasoned that when participants control both task execution and strategy, they become overly focused on task execution. However, we did not find evidence that adding agency over task execution moved participants away from the optimal strategy on average.

Although agency had a minimal effect on decisions in this experiment, even the ‘complex’ control scheme used in Experiment 3 was an indirect and simpler execution than those of previous focus-divide experiments (e.g., *throwing*; Clarke & Hunt, 2016). Increasing difficulty further may shift participants' attention away from improving their strategy to improving their ability to execute the task, producing results more like those of previous experiments. Task difficulty aside, an important difference remains between controlling the motion of a cartoon truck and actually driving a truck. We attempted to manipulate agency over the task outcome by making the driving task more difficult, which meant that participants’ task execution had more bearing on whether the goal on each trial was achieved. But even when participant performance more strongly influences the outcome, an important difference remains between controlling the motion of a cartoon truck and actually driving a truck: The driver of a ‘real’ truck would experience the consequences of their parking choices from a first-person perspective. Manipulating this personal sense of agency could be achieved more easily using the beanbag-throwing task, in which agency over the outcome is already high. A manipulation that could reduce agency in this context would be to make the choices about standing position on behalf of someone else, to separate solving the focus-divide dilemma from executing the throw. If reduced agency over the task is the reason performance was better in the truck task than throwing, when a participant decides where to stand on behalf of someone else, we should see a similar boost in the quality of decisions for standing position as we did for decisions about where to park the truck. There is some existing evidence that decisions differ when deciding for oneself versus when others bear the consequences (Polman & Wu, 2020). For decisions involving risk, Polman and Wu’s (2020) meta-analysis of 71 studies found evidence in support of a ‘risky shift’; an increased risk preference on behalf of others. For preferences and insight problems, participants search among a wider range of alternatives and are better able to come up with solutions when deciding for others rather than for themselves (Liu et al., 2018; Polman & Emich, 2011). With this in mind, we may yet find an agency effect in the focus-divide dilemma based on a manipulation of making decisions for oneself versus for an avatar or another individual.

### Uncertainty

That uncertainty shifted decisions away from optimal is consistent with the one other focus-divide task in which participants have previously exhibited optimal behaviour: *reaching* (Clarke & Hunt, 2016). In that task, it was certain and obvious whether the targets were inside or outside of their reach, in contrast to other versions, such as *throwing* (Clarke & Hunt, 2016), in which choice outcomes were defined by a probability curve which dropped with distance. Participants make uniformly optimal choices about where to sit to reach for a beanbag on a table, and highly variable and suboptimal decisions about where to stand to throw a beanbag at a hoop. The fire truck task allowed us to more systematically manipulate uncertainty by adding variability to the truck’s driving speed. More variable driving speeds had a small but consistent effect of reducing the size of the adjustment of parking position choices with the distance between the houses. This occurred despite the fact that the optimal strategy is the same (park in the centre when the houses are close, park next to one house when they are far apart), with the only difference between speed conditions being whether the outcome on each trial is highly probable versus completely predictable. An important limitation to mention here is that we were not able to model choices in the two middle distances because the optimal strategy differed between individuals across some conditions for these two distances. It is likely that for the less extreme distances the difference between uncertain choice outcomes and certain ones would be more pronounced, which would make the effect of uncertainty here an underestimate. We expect this effect because uncertain estimates of the truck’s success rate in the variable condition are more likely to interfere with strategic decisions for the middle distances, as the truck’s success rate from the centre is closer to the critical 50% threshold relevant to the optimal strategy. At the closest and furthest distances, uncertain estimates of the truck’s success rate make less of a difference, because the outcome is well above and below the threshold.

Across the three experiments we also found correlations between different confidence metrics and the adjustment effect. Apart from one of the metrics of Experiment 1, moderate to strong positive correlations (see OSM sections A–C) were found between how confident participants were to judge if the truck could reach the house or not, and how close they got to optimal choices in the decision phase of the experiment. Taken with the direct effect of Constant vs. Variable speed, the results imply that uncertainty undermines strategic choices in the focus-divide dilemma.

A similar effect occurs in the explore-exploit dilemma, a decision problem in which an agent seeking to maximise their rewards must decide whether to exploit known options or explore the unknown. In Wilson et al. (2014), participants made repeated decisions between two virtual slot-machines with the goal of maximising rewards. Rewards from each machine were drawn from gaussian distributions with different means, such that one option was better than the other. Participants were made aware of these characteristics but were not told which machine produced the greater average reward. Requiring participants to sample the machines in a particular sequence for the first few trials enabled control of experience with each option. Less experience was associated with increased variability, eliciting random exploration which decreased over the course of the trials. When experience was unequal (i.e., one machine was sampled more than the other), participants favoured the machine they had less information about, exhibiting directed exploration of the uncertain option. Similarly, Gershman (2019) independently manipulated total and relative uncertainty in a multi-armed bandit task. More total uncertainty elicited more random variation, and consequently a flatter slope of the choice probability function. When there was a difference in relative uncertainty this shifted the intercept of the choice probability function towards the uncertain option. Gershman (2019) draws a parallel between machine learning algorithms and human choice, suggesting an underlying uncertainty computation (total or relative uncertainty) guides decision making.

Returning to the present findings, we may see worse decisions with increased spread under uncertain conditions in the fire trucks task because of an increase in random exploration triggered by uncertainty, analogous to the response selection effects observed in the explore-exploit dilemma (Gershman, 2019; Wilson et al., 2014). Aside from adding variability to response selection, others have argued that uncertainty makes inferences imprecise, and that this imprecision is a key source of behavioural variability (Beck et al., 2012; Drugowitsch et al., 2016; Findling et al., 2019; Wyart & Koechlin, 2016). This may explain why uncertainty makes decisions worse in our experiments, as imprecise estimates of the fire truck’s chances of success from a given distance make the point at which one should switch from a *divide* strategy to a *focus* strategy less clear. It is not clear yet how these different explanations for the role of uncertainty in the focus-divide dilemma may be resolved, but first, it seems important to return to previous versions, such as *throwing* (Clarke & Hunt, 2016), to assess whether outcome uncertainty can be leveraged to shift participants towards the optimal strategy. In future research it may be possible to design a version of the dilemma which can reveal the contribution of different components of the decision process to suboptimal variability.

A final important note is that the shift away from optimal choices associated with variable speeds was not sufficiently large to provide a complete explanation for why participants are more optimal with the current task than with other versions of the focus-divide dilemma. Even the condition where participants performed most poorly in the current series of virtual tasks (i.e the complex manual task in Experiment 3, under variable speed conditions) is still a vast improvement on performance in previous experiments (Clarke & Hunt, 2016; James et al., 2019; James et al., 2023), indicating there is a large gap left to explain. Future research could explore both the size and the source of uncertainty as potential explanations. The computerised version of the task permits other manipulations of uncertainty, such as trucks that accelerate or decelerate, or houses that burn down more or less quickly. In previous versions of the focus-divide dilemma, participants have high performance demands and the uncertainty comes from internal perceptual, cognitive and motor noise associated with task execution. The diversity or source of this noise may have an impact on how it affects decisions. There is also a potentially important difference in agency between controlling an avatar and carrying out the task for one’s self, even when the control of the avatar is difficult. Direct physical responsibility for task execution could contribute to the failure in these previous tasks to prioritise effectively. By creating conditions in which participants engage in effective prioritisation, the current study opens key avenues for understanding how we make choices under uncertainty, with practical applications for how to provide contexts that promote more effective decisions.

### Supplementary information

ESM 1 (PDF 838 kb)

## Data Availability

The data and materials for all experiments are available on the Open Science Framework (https://osf.io/4yv7m/). None of the experiments were pre-registered.

## Code Availability

The code for each of the experiments are available on the Open Science Framework (https://osf.io/4yv7m/).
