# Supplementary material for: The role of framing, agency and uncertainty in a focus-divide dilemma
Source: Mem Cognit. 2023 Nov 3;52(3):574–94. doi: 10.3758/s13421-023-01484-6 (PMC11021327; doi:10.3758/s13421-023-01484-6)
Supplement: Supplementary file 1 — (PDF 838 kb) [file 13421_2023_1484_MOESM1_ESM.pdf]

The role of framing, agency and uncertainty in a focus-divide dilemma: Supplementary material

Justin Claydon*, Warren R.G. James**, Alasdair D.F. Clarke*** and Amelia R. Hunt*

Corresponding author details:
Justin Claydon
School of Psychology, University of Aberdeen
Aberdeen, UK AB24 3UB
+44 7515094439
j.claydon.21@abdn.ac.uk

*School of Psychology, University of Aberdeen
**School of Medical Sciences, University of Aberdeen
***School of Psychology, University of Essex

Table of contents

Section A: Supplementary material for Experiment 1 ...………………………………………… 1

Estimation phase ...……………………………………………………………………. 1

Learning phase check ………………………………………………………………. 9

Decision phase …………………………………………………………………… 11

Section B: Supplementary material for Experiment 2 …………………………………………. 17

Power analysis ……………………………………………………………………. 17

Estimation phase ………………………………………………………………….. 19

Learning phase check ……………………………………………………………... 21

Decision phase …………………………………………………………………… 23

Section C: Supplementary material for Experiment 3 …………………………………………. 31

Estimation phase ………………………………………………………………….. 31

Learning phase check ……………………………………………………………... 34

Decision phase …………………………………………………………………… 36

Section A: Supplementary material for Experiment 1

Estimation phase

Figure S1

Yes-no answers in the estimation phase of Experiment 1

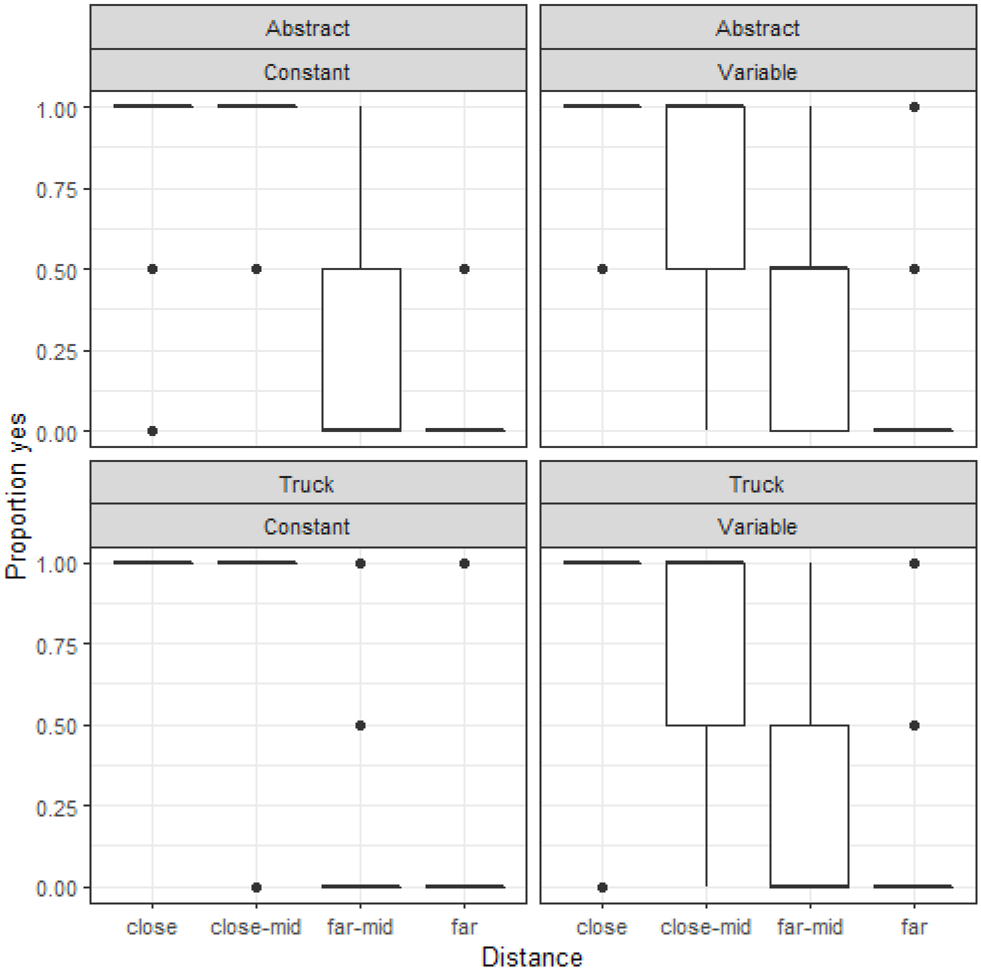

Note. Mean proportion of “yes” answers to the question “Will the (avatar) reach the target?” in the estimation phase of Experiment, shown separately by speed type and framing condition. Distances are standardised across the two different screen resolution groups.

Figure S2

Confidence ratings from the estimation phase

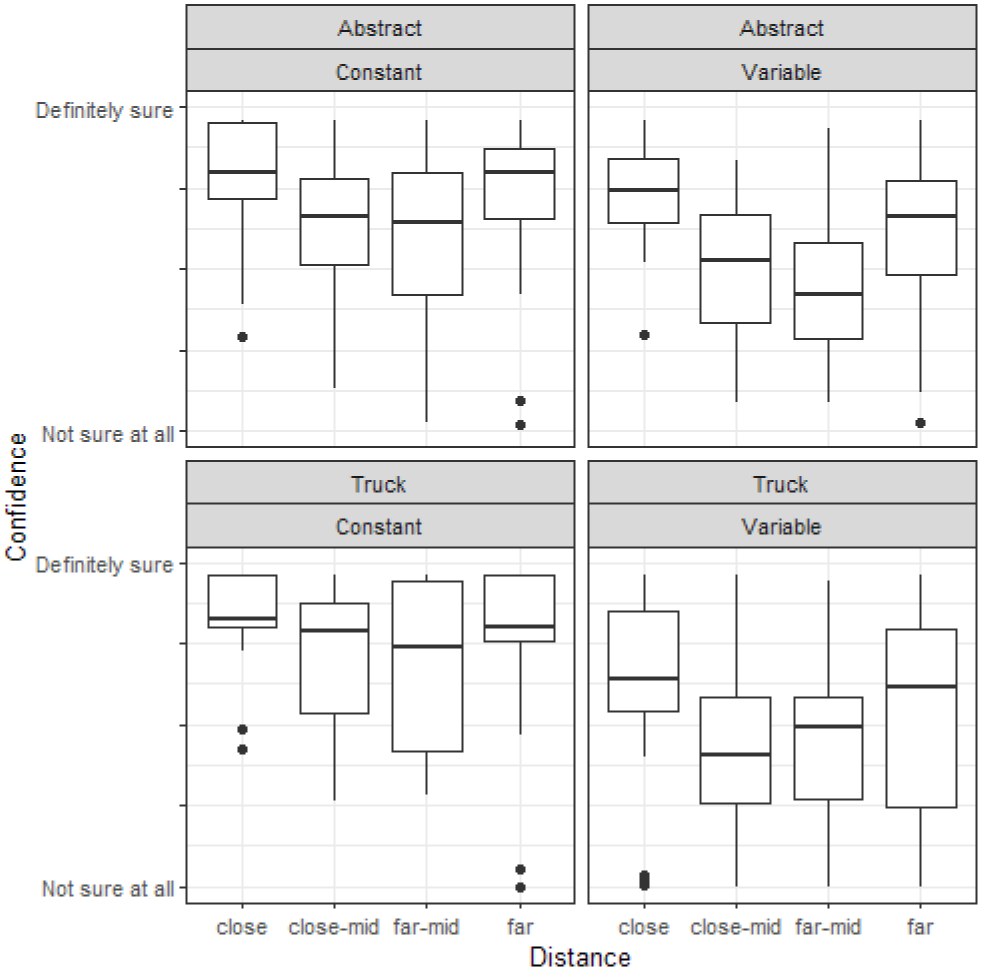

Note. Mean confidence ratings from the estimation phase of Experiment 1, shown separately by speed type and framing condition. Participants were asked “Will the avatar reach its target?”, and positioned a slider on a continuous scale between “Not sure at all” and “Definitely sure”. Distances are standardised across the two different screen resolution groups.

Figure S3

Scatter plot showing the adjustment effect against confidence ratings by Speed type

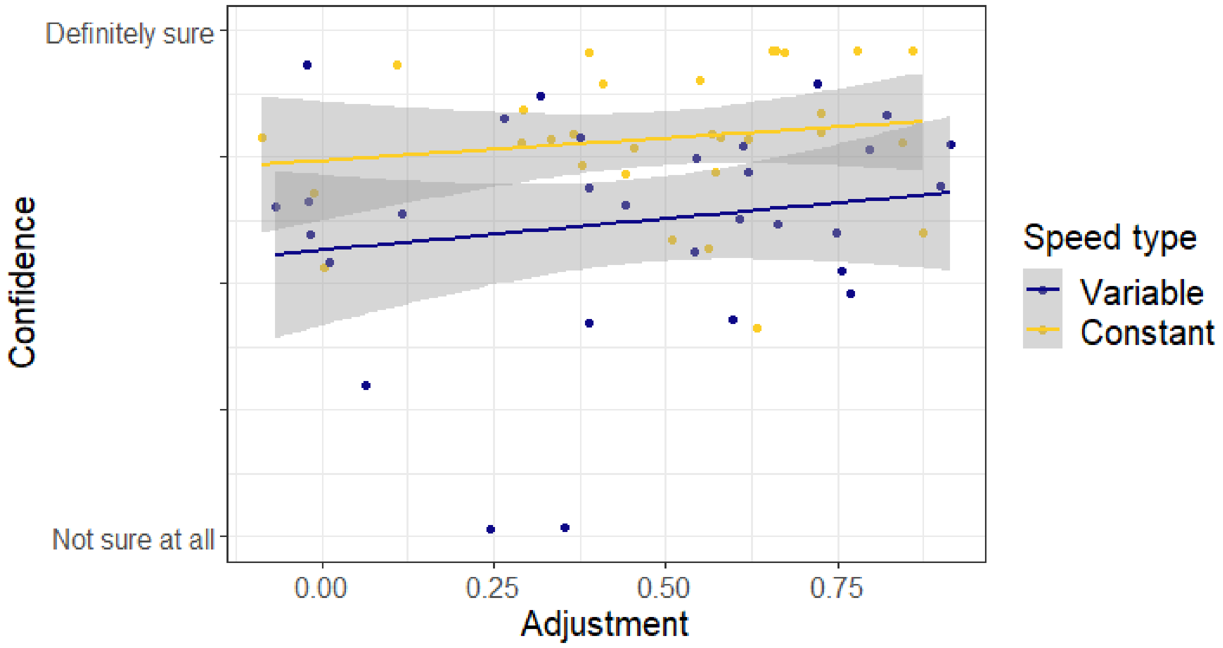

Note. This scatter plot shows the relationship between confidence ratings and the adjustment effect (Far-Close) in Experiment 1. Participants were asked “Will the avatar reach its target?”, and positioned a slider on a continuous scale between “Not sure at all” and “Definitely sure”. Each individual contributed two points to the plot as Speed type was manipulated within-subjects.

Correlation between difference in confidence and difference in adjustment

Figure S4 shows the relationship between the difference in certainty (Variable - Constant) and difference in adjustment (Variable - Constant). The correlation (r(28) = 0.13) was not significant at alpha = .05.

Figure S4

Relationship between difference in adjustment and confidence by Speed type

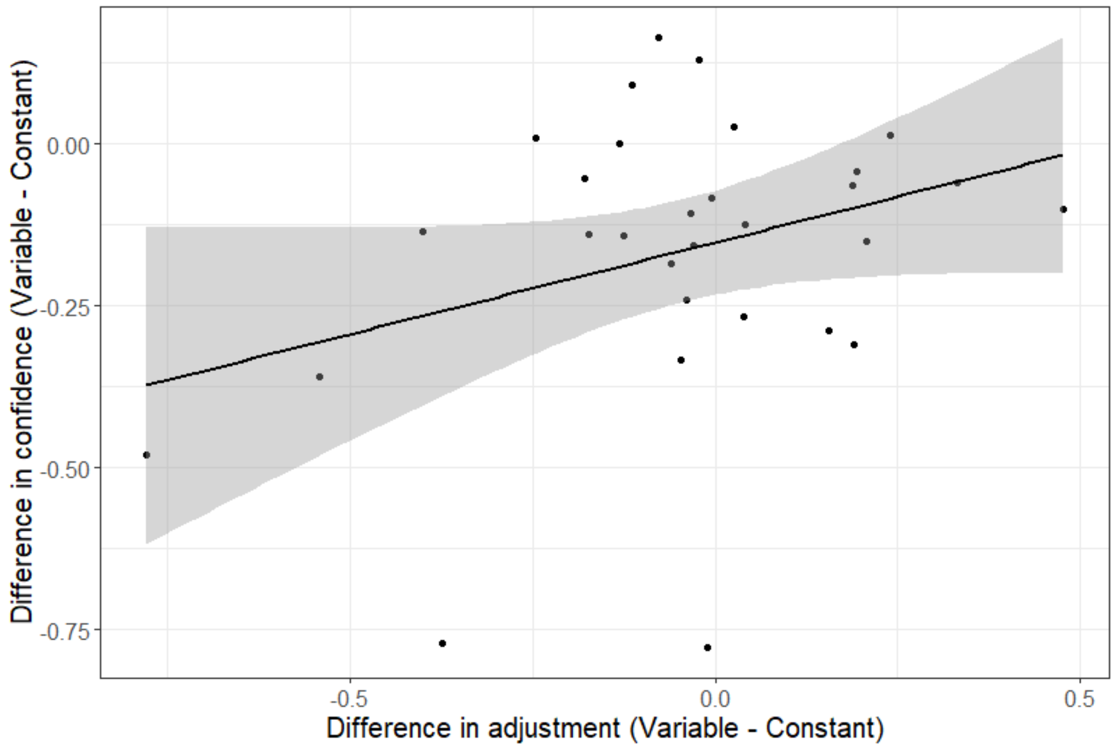

Certainty-Adjustment correlations by speed type

Figure S5 shows the relationship between the certainty metric and the adjustment effect. The correlation was significant for the Constant condition, $r(28) = 0.37$, $p = 0.045$, but not the Variable condition $r(28) = 0.37$, $p = 0.07$.

Figure S5

Relationship between certainty and adjustment by Speed type

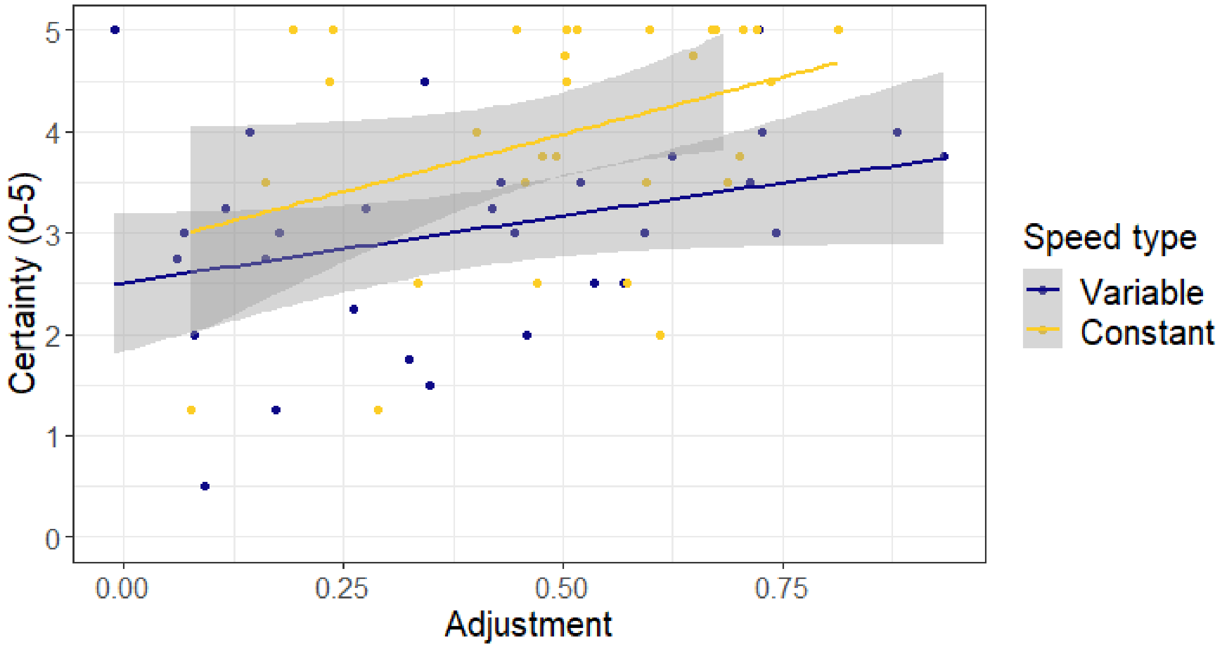

Note. This scatterplot shows the relationship between adjustment (Far-Close) and the certainty metric for Experiment 1, separated by Speed type. Participants indicated how many times they thought the truck would be successful out of 10 trials at each distance. The absolute difference from the midpoint (5) was calculated, indicating whether participants thought the avatar would reliably reach or fail to reach the target.

Correlation between difference in certainty and difference in adjustment

Figure S6 shows the relationship between the difference in certainty (Variable - Constant) and difference in adjustment (Variable - Constant). The correlation (r(28) = 0.13) was not significant at alpha = .05.

Figure S6

Relationship between difference in adjustment and certainty by Speed type

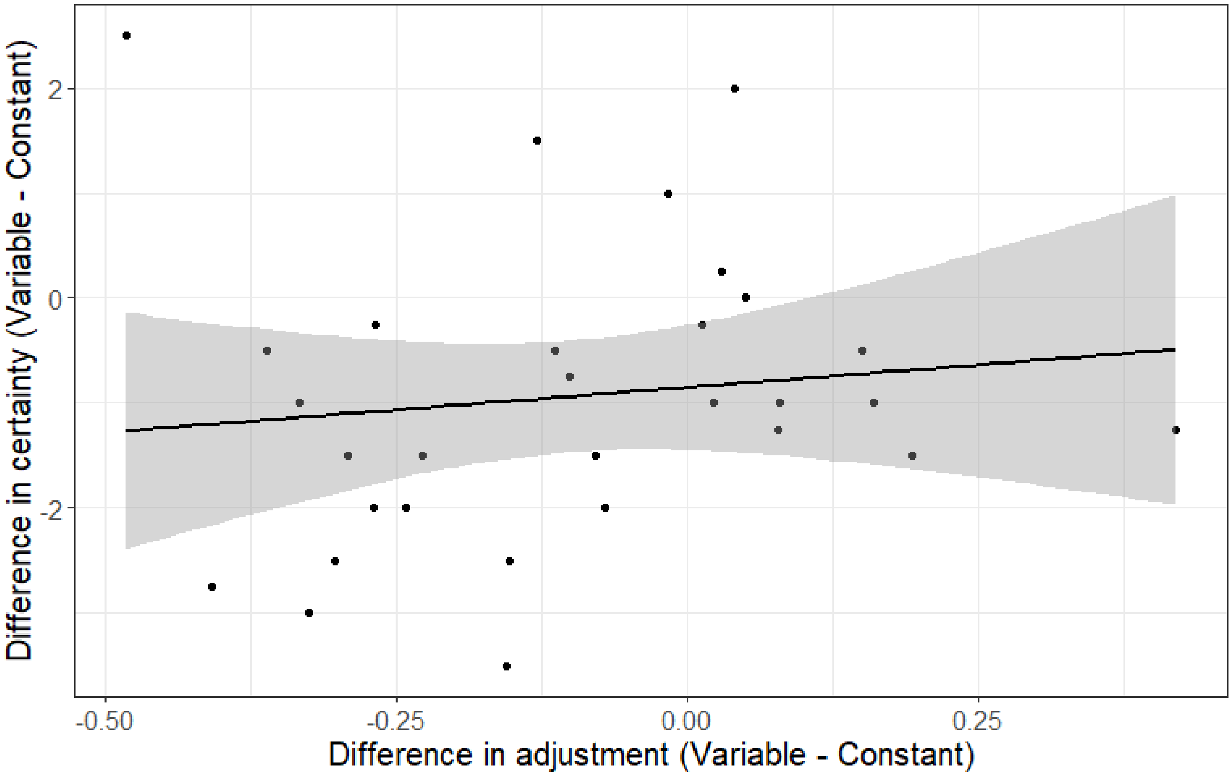

Note. This scatterplot shows the relationship between the difference in adjustment (Variable - Constant) and certainty. Participants indicated how many times they thought the truck would be successful out of 10 trials at each distance. The absolute difference from the midpoint (5) was calculated, indicating whether participants thought the avatar would reliably reach or fail to reach the target.

Figure S7

Estimated successes out of 10 trials

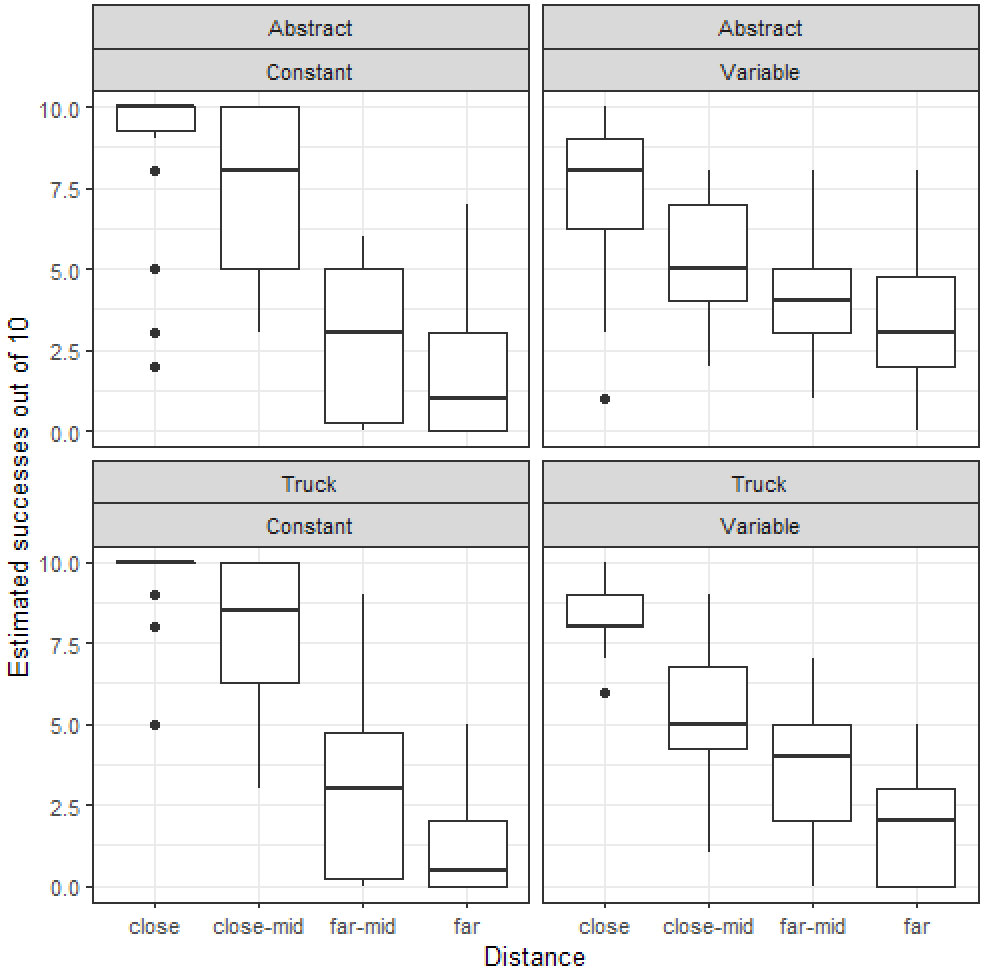

Note. Estimated successes out of 10 trials at each distance reported by participants in the estimation phase of Experiment 1, shown separately by speed type and framing condition. Distances are standardised across the two different screen resolution groups.

Learning phase check

As the performance of the avatar differed by condition (Variable or Constant), Bayesian binomial regression was performed on the Learning phase accuracy data to check the different conditions were still comparable in terms of the optimal strategy at each of the four separations used in the Decision phase. That is, that the accuracy at the two closest distances was above 50%, and below 50% at the two furthest distances. Because participants saw two different screen resolutions, separate models were fit to check that this did not cause a difference in the optimal strategy. Twenty-two participants had a screen resolution of 1920x1080, and 38 had a screen resolution of 1600x900. This means that the normalised distances relevant to the accuracy check were closest (0.23 or 0.29, for 1920x1080 and 1600x900 respectively), close-middle (0.49 or 0.52), far-middle (0.74 or 0.76) or furthest (1.0). The results of the models (Figure S8) demonstrated that there was a difference across the conditions in terms of accuracy by distance. The optimal strategy remained the same across the conditions and different resolutions apart from the Variable condition of the 1600x900 group. For this group, accuracy at the close-middle distance was not clearly above 50%. As such, comparing truck placement collapsed across the two closest and furthest distances for the decision phase would not be valid. However, the optimal strategy at the closest and furthest distances was the same, meaning comparisons at these distances remain valid. Participants should have placed the avatar in the middle when the targets were at the closest distance, and adjacent to one of them at the furthest separation. As such, the rest of the analysis was carried out on just the closest and furthest distances.

Figure S8

Binomial regression fit to learning phase data

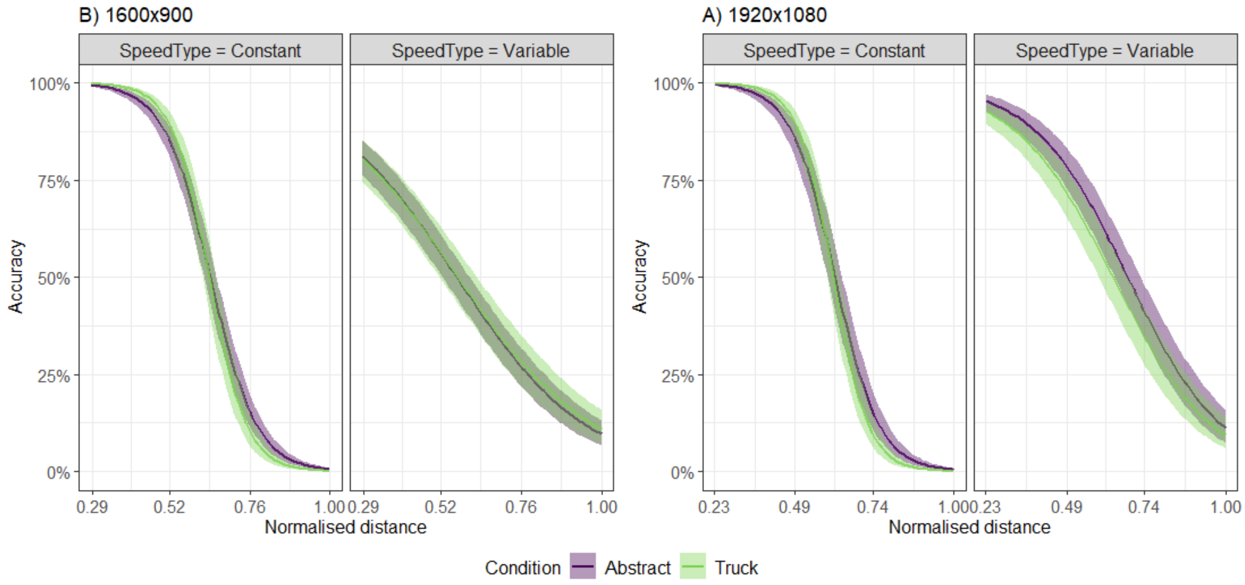

Note. A) These plots show the conditional effects of a binomial regression fit to the Learning phase data for each Speed type (Constant or Variable) framing (Concrete vs Abstract) for the participants who completed the experiment with a 1920x1080 screen resolution and the same (B) for the participants who completed the experiment with 1600x900 screen resolution.

Decision phase

Figure S9

Normalised truck positions chosen by each participant

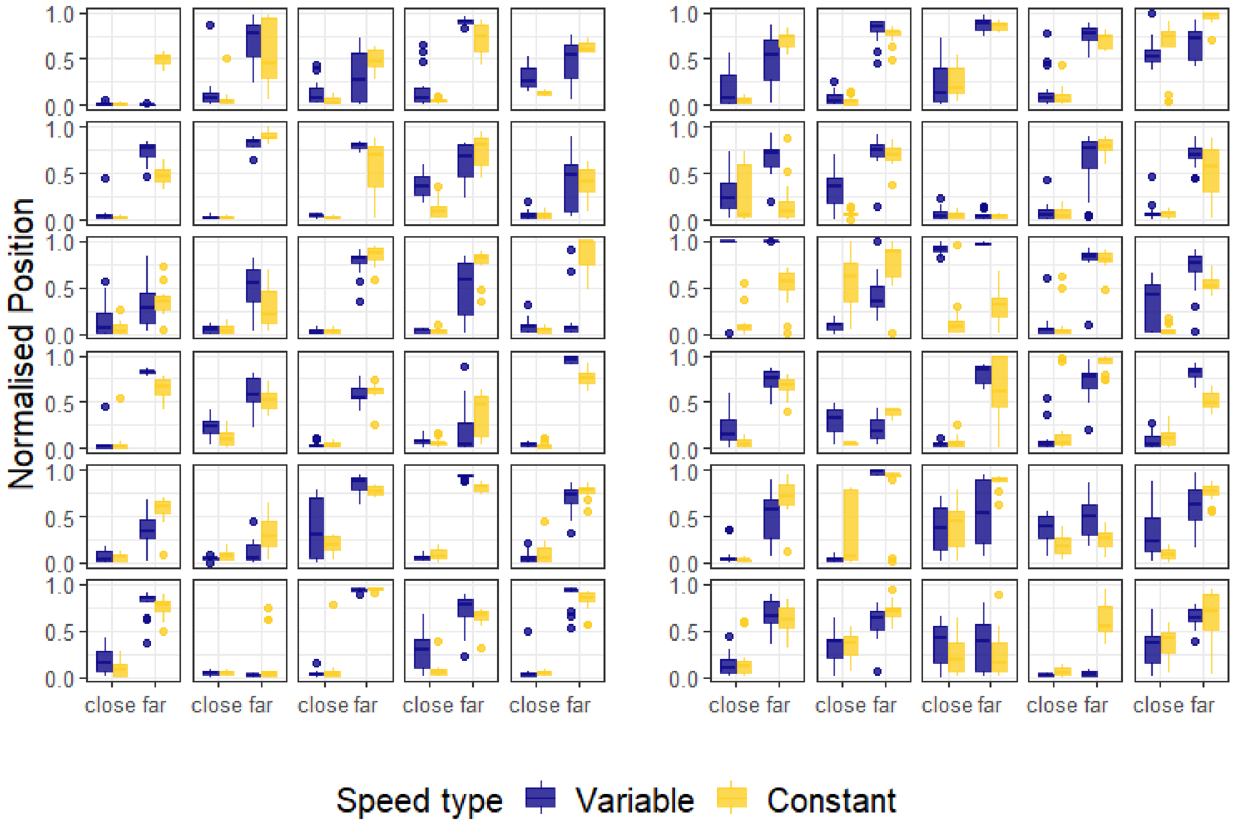

Note. Facets show the normalised truck positions chosen by each participant in the Truck (left) and Abstract (right) conditions, coloured separately by Speed type (Constant or Variable).

Figure S10

Summary of normalised truck positions chosen participants including the middle distances

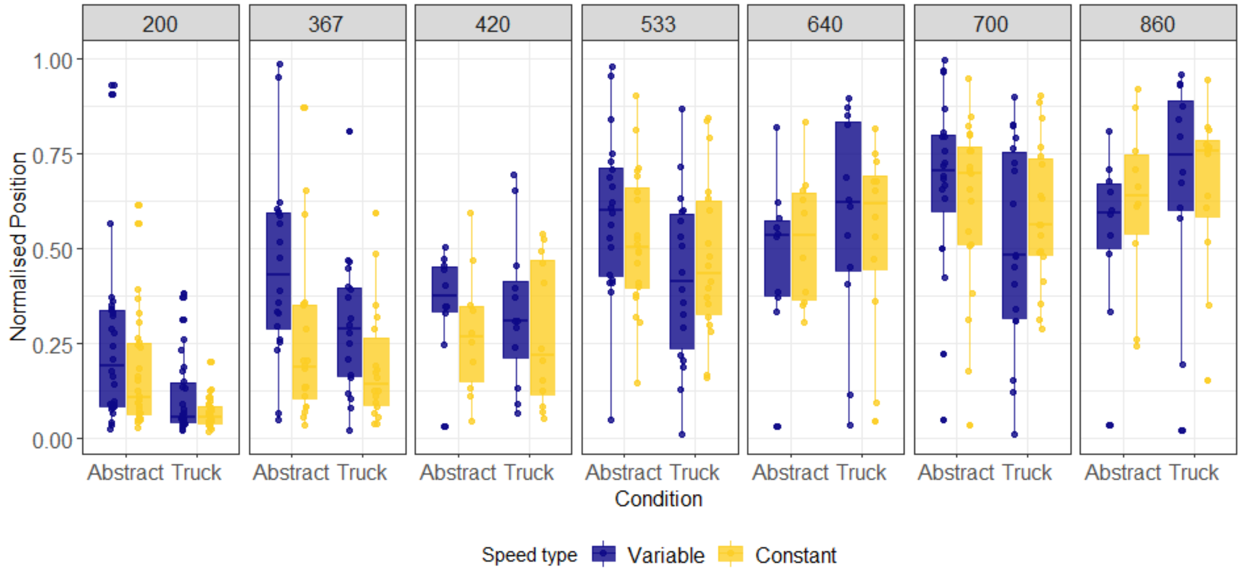

Note. Summary plots for the decision phase data in Experiment 1, plotted separately for each inter-target distance (from 200 to 860 pixels). The dots show the mean normalised truck position chosen by each participant in the Truck and Abstract framing conditions, coloured separately by uncertainty (Constant or Variable).

Figure S11

Prior prediction

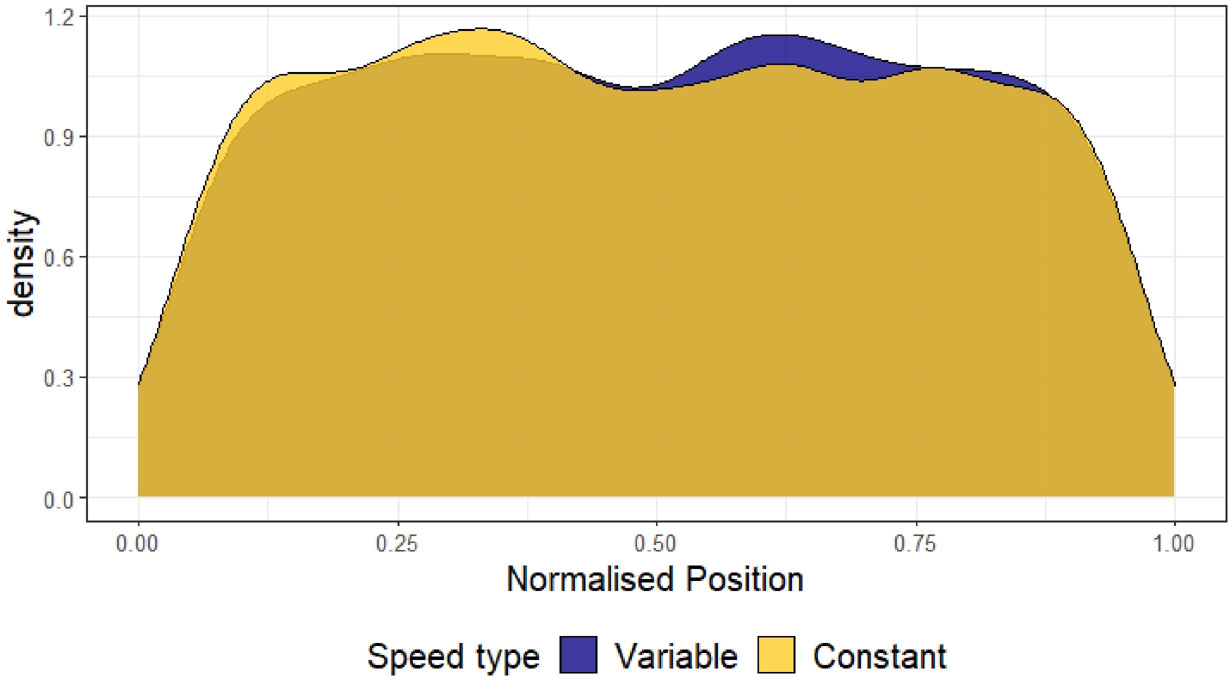

Note. This plot shows the prior predictions of the Bayesian beta regression model fit using the decision data of Experiment 1. The same prior was used for all subsequent analyses, but is presented here only, to avoid repetition.

Figure S12

Model predictions including random effects

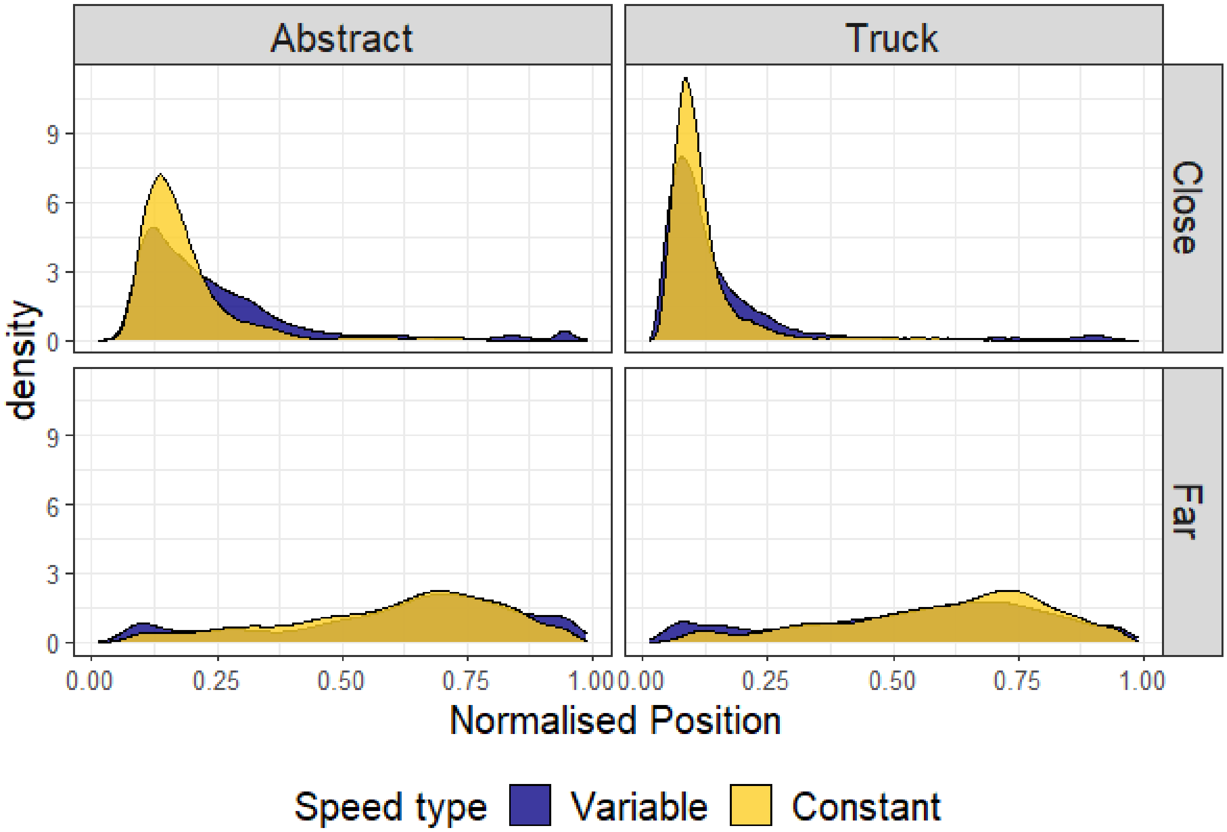

Note. Facets show the posterior predicted means from Bayesian beta regression model fit to the data in Experiment 1, including the random effects structure.

Adjustment-confidence correlations

Positive correlations here would indicate that participants expressing greater confidence about the outcome of each trial adhere to the optimal strategy more than those who were less confident. Average ratings of confidence (Far and Close) from the estimation phase and adjustment had a weakly positive relationship[1], $r(28) = 0.11$, $p = 0.573$ (see supplementary material section A for a scatterplot). For the analysis of estimated successes out of 10, the absolute difference from the midpoint (5) was calculated, such that scores (0-5) indicate how reliably participants thought the avatar would reach or fail to reach the target. This certainty metric had a more strongly positive correlation with adjustment, $r(28) = 0.44$, $p = 0.014$. Figure S13 shows the relationship between adjustment and certainty. Note that although we report p-values, we did not have specific hypotheses for any of the correlations reported in the paper and supplementary materials.

Figure S13

Scatterplot of certainty against adjustment

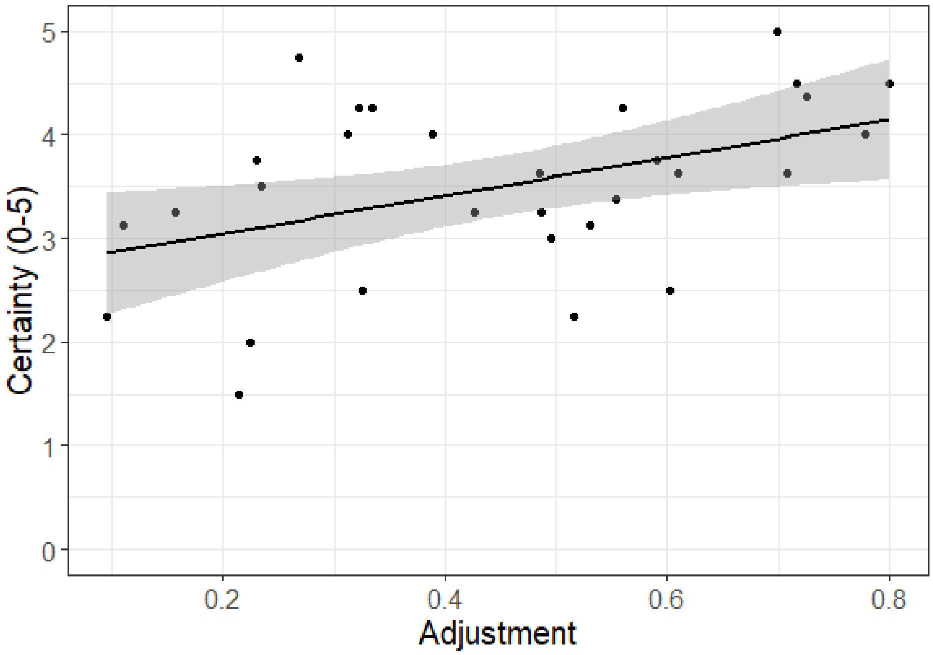

Note. This scatterplot shows the relationship between adjustment (Far-Close) and the certainty metric for Experiment 1. Participants indicated how many times they thought the truck would be successful out of 10 trials at each distance. The absolute difference from the midpoint (5) was calculated, indicating whether participants thought the avatar would reliably reach or fail to reach the target. The correlation was carried out on the data collapsed across conditions. For scatter plots separated by Speed type, see the supplementary materials section A.

Section B: Supplementary material for Experiment 2

Power analysis

Data from the *throwing* task (Clarke & Hunt, 2016) and Experiment 1 allowed us to perform a power analysis to justify our sample size. We simulated the difference in adjustment we can expect to see by giving participants control over the motion of the truck. In Experiment 1, control was restricted to deciding where to place the firetruck between two potential target houses which varied in separation, after which they would watch the truck attempt to reach the target house in time. The *throwing* task dataset (Clarke & Hunt, 2016) was used to simulate the effect of giving participants control, because in this experiment they controlled both the strategic decision and the subsequent performance element of the task. One thousand samples were taken from each data set, with replacement, for increasing sample sizes from 2 to 30. For each sample, the adjustment effect (Far-Close), and the difference between adjustment effects was calculated. To determine our sample size, 95% confidence intervals for the difference were calculated for each sample. As Figure S14 shows, the uncertainty around the difference in the adjustment effect plateaued around N = 18. Based on this analysis, we can say that our conclusions in Experiments 2 and 3 were unlikely to change with a larger sample size, as our sample sizes per condition were greater than N = 18 in all cases.

Figure S14

How the uncertainty around the difference in adjustment changes with simulated sample size

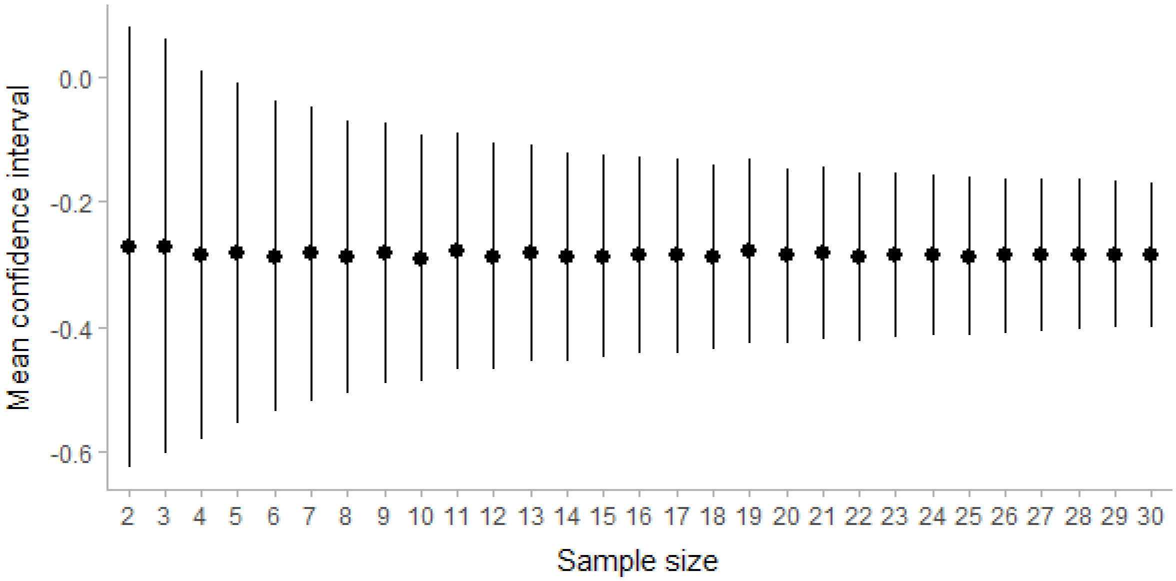

Note. Mean 95% confidence intervals for the simulated adjustment effect in Experiments 2 and 3, based on one thousand samples from the *throwing* task (Clarke & Hunt, 2016) and the truck condition from Experiment 1. Uncertainty around the simulated adjustment effect plateaus at sound N = 18.

Estimation phase results

Figure S15

Proportion of 'yes' responses in the estimation phase

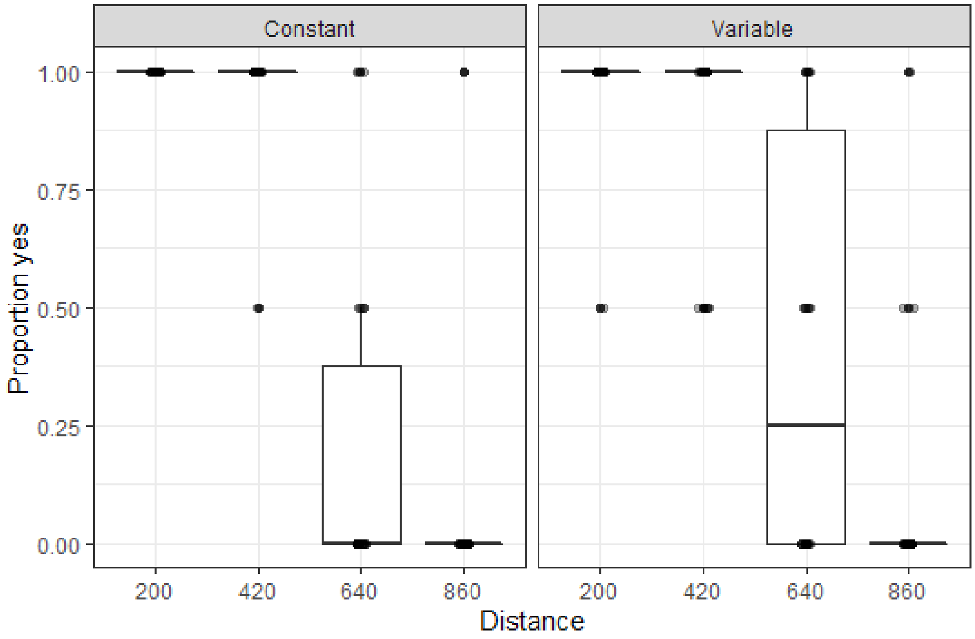

Note. Proportion of "yes" responses to the question "Will the firetruck reach its target?". Note that these data are for the Manual condition only, as the automatic condition was drawn from Experiment 1, which can be seen in Section A.

Figure S16

Confidence ratings from the estimation phase

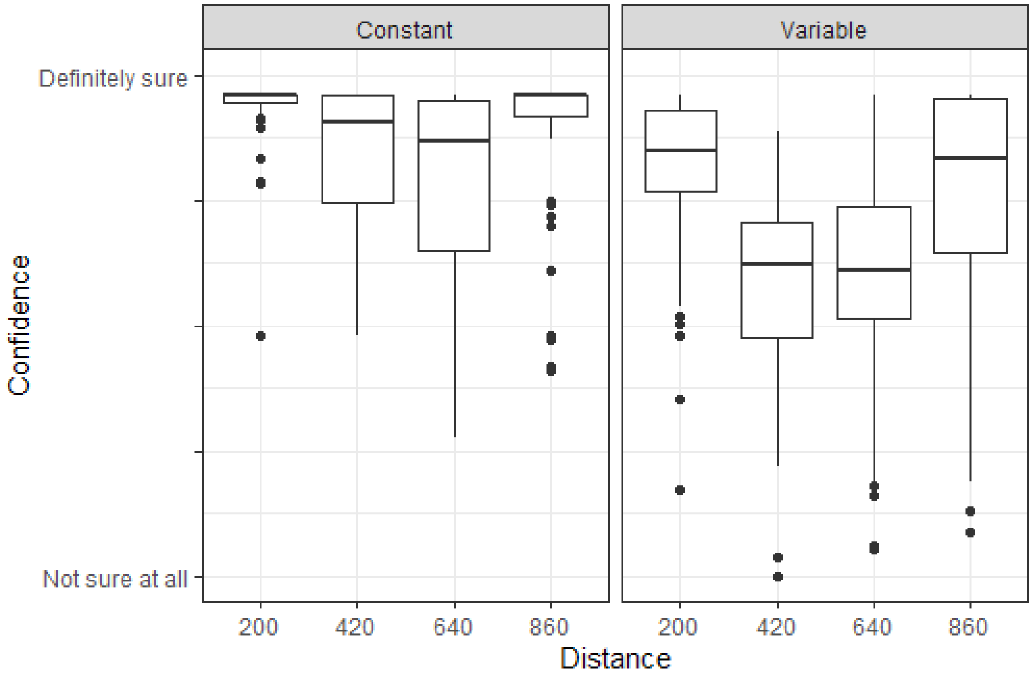

Note. Mean confidence ratings from the estimation phase of Experiment 2. Participants were asked “Will the avatar reach its target?”, and positioned a slider on a continuous scale between “Not sure at all” and “Definitely sure”. Note that these data are for the Manual condition only, as the automatic condition was drawn from Experiment 1, which can be seen in Section A.

Learning phase check

As for Experiment 1 (Section A of supplementary materials), Bayesian binomial regression was performed on the Learning phase accuracy data to check if the different conditions were comparable in terms of the optimal strategy at each of the four separations used in the Decision phase. That is, that the accuracy at the two closest distances was above 50%, and below 50% at the two furthest distances. Because participants saw two different screen resolutions, separate models were fit to check that this did not cause a difference in the optimal strategy. Forty-two participants had a screen resolution of 1920x1080, and 18 had a screen resolution of 1600x900. This means that the normalised distances relevant to the accuracy check were closest (0.23 or 0.29, for 1920x1080 and 1600x900 respectively), close-middle (0.49 or 0.52), far-middle (0.74 or 0.76) or furthest (1.0). The results from these models can be seen in Figure S17. There was a difference across the conditions in terms of accuracy. All the conditions across each resolution group were comparable in terms of the optimal strategy, apart from the variable condition of the 1600x900 group. Here the accuracy at the close-middle distance (0.52) was not clearly above 50%. The optimal strategy remained the same for the closest and furthest distances, so it is still valid to compare conditions and groups in terms of truck placement for the decision phase. Participants should have placed the firetruck in the middle for the closest distance, and adjacent to one of the houses for the furthest distance. The rest of the analysis was conducted on these distances.

Figure S17

Binomial regressions fit to the Learning phase data

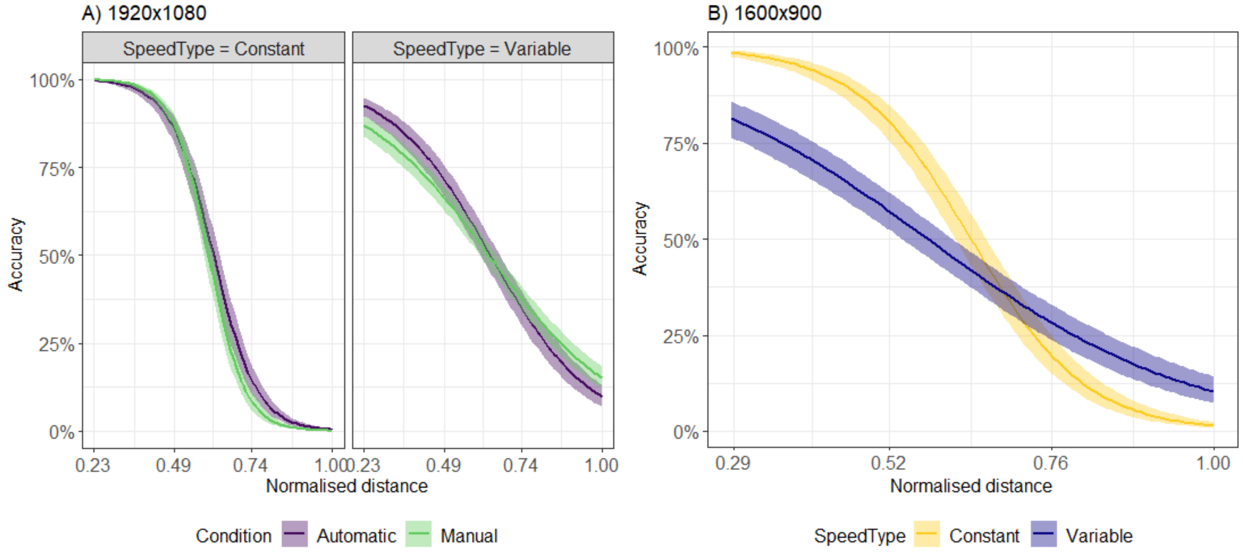

Note. A) These plots show the conditional effects of a binomial regression fit to the Learning phase data for each level of uncertainty (Constant or Variable) agency (Manual vs Automatic) for the participants who completed the experiment with a 1920x1080 screen resolution. B) These lines show the conditional effects for level of uncertainty (all these participants were in the automatic group) for the participants who completed the experiment with 1600x900 screen resolution.

Decision phase

Figure S18

Normalised truck positions chosen by each participant

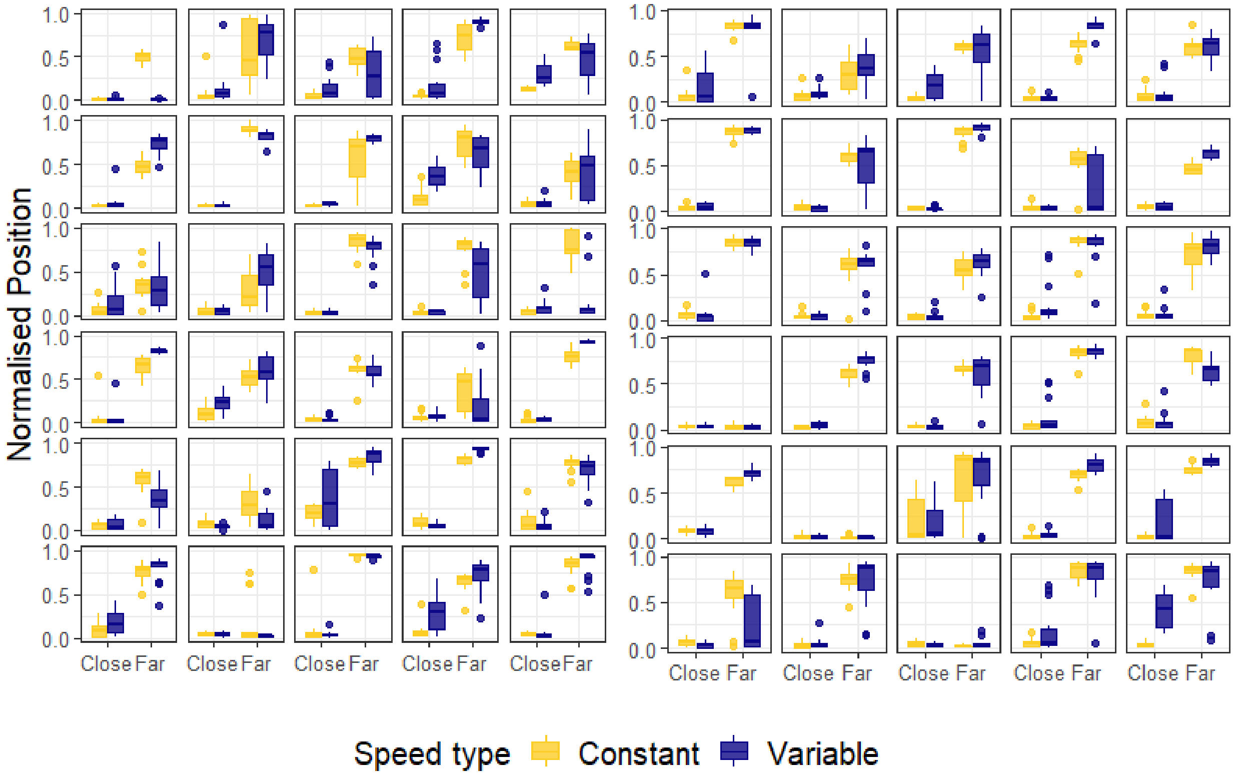

Note. Facets show the normalised truck positions chosen by each participant in the Automatic (left) and Manual (right) conditions of Experiment 2, coloured separately by Speed type (Constant or Variable).

Figure S19

Summary of normalised truck positions chosen by participants including the middle distances

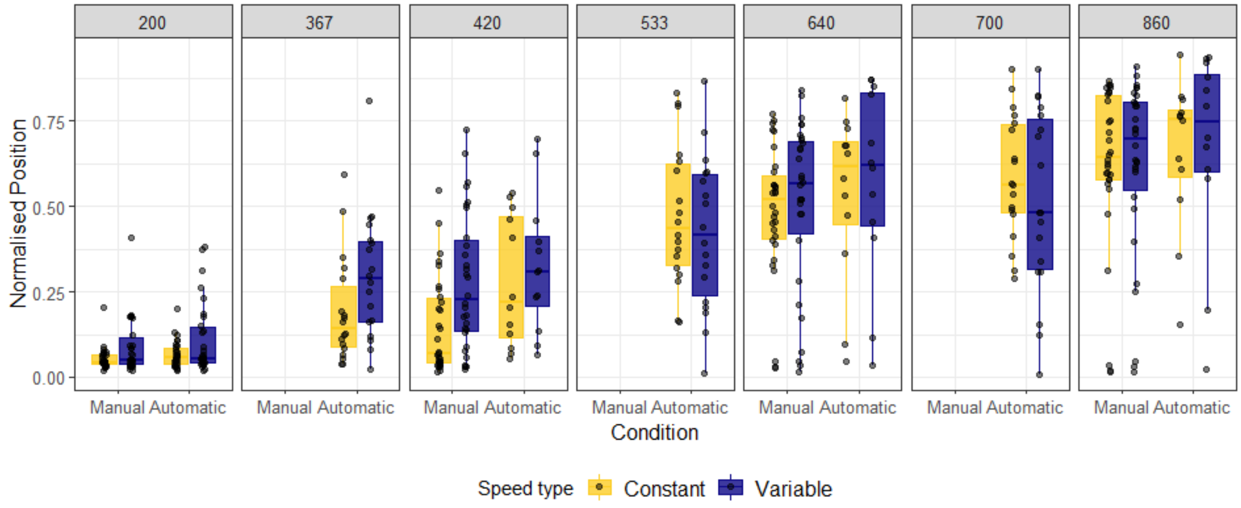

Note. Summary plots for the decision phase data in Experiment 2 shown for all the inter-house distances included in the experiment. The dots are the mean normalised truck positions chosen by each participant in the Truck and Abstract framing conditions, coloured separately by uncertainty (Constant or Variable).

Figure S20

Model predicted means including the random effects structure

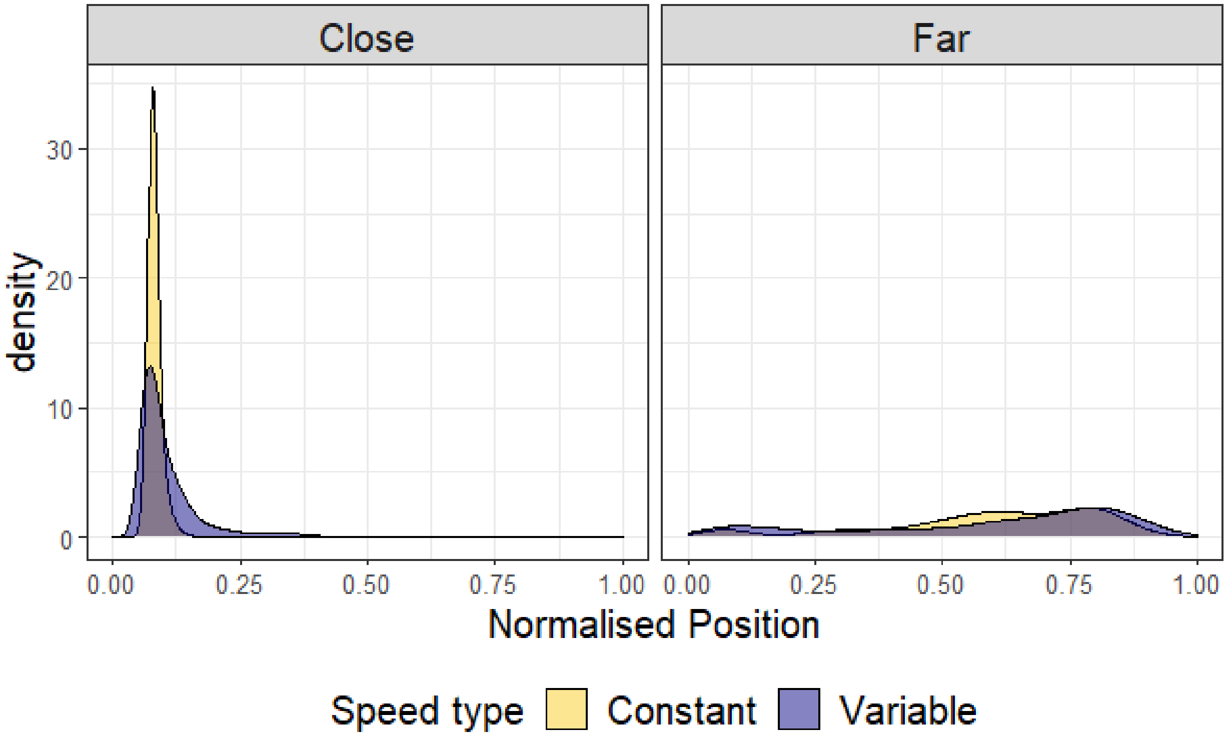

Note. These plots show the predicted means for the manual condition of the Bayesian beta regression model fit to the decision data of Experiment 2 and the truck condition of Experiment 1, including the random effects structure.

Figure S21

Experiment 1 order effects model, framing adjustment effect

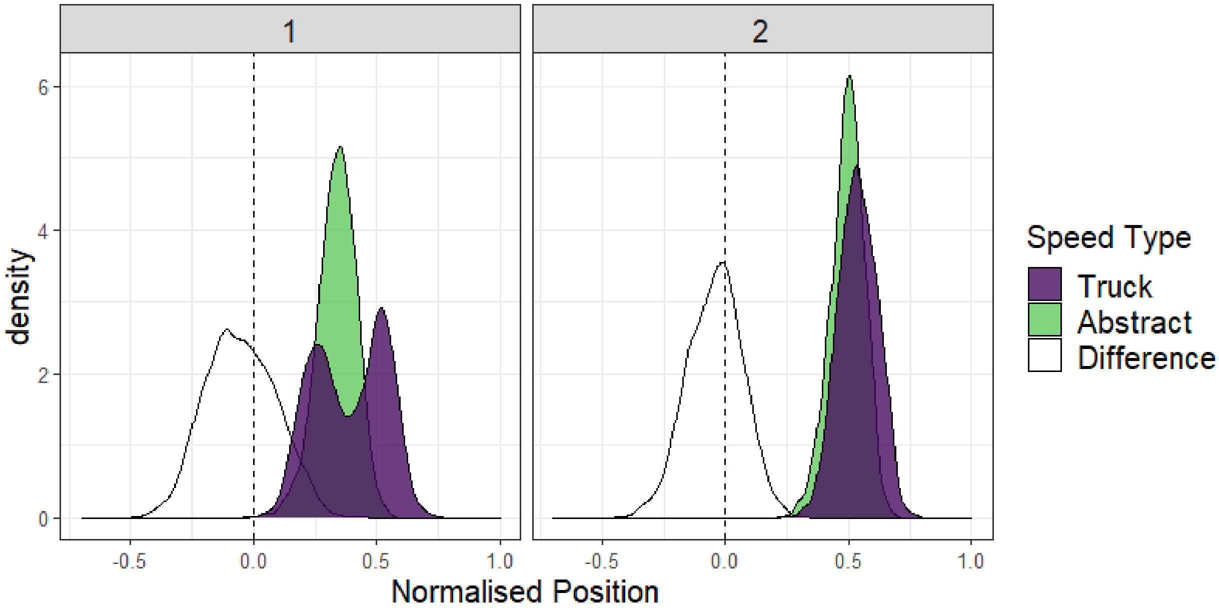

Note. The posterior predicted adjustment effect (the difference between the Far and Close conditions) for blocks 1 and 2. The white distributions show the difference in adjustment of position with distance between abstract and truck conditions.

Figure S22

Experiment 2 order effects model, agency adjustment effect

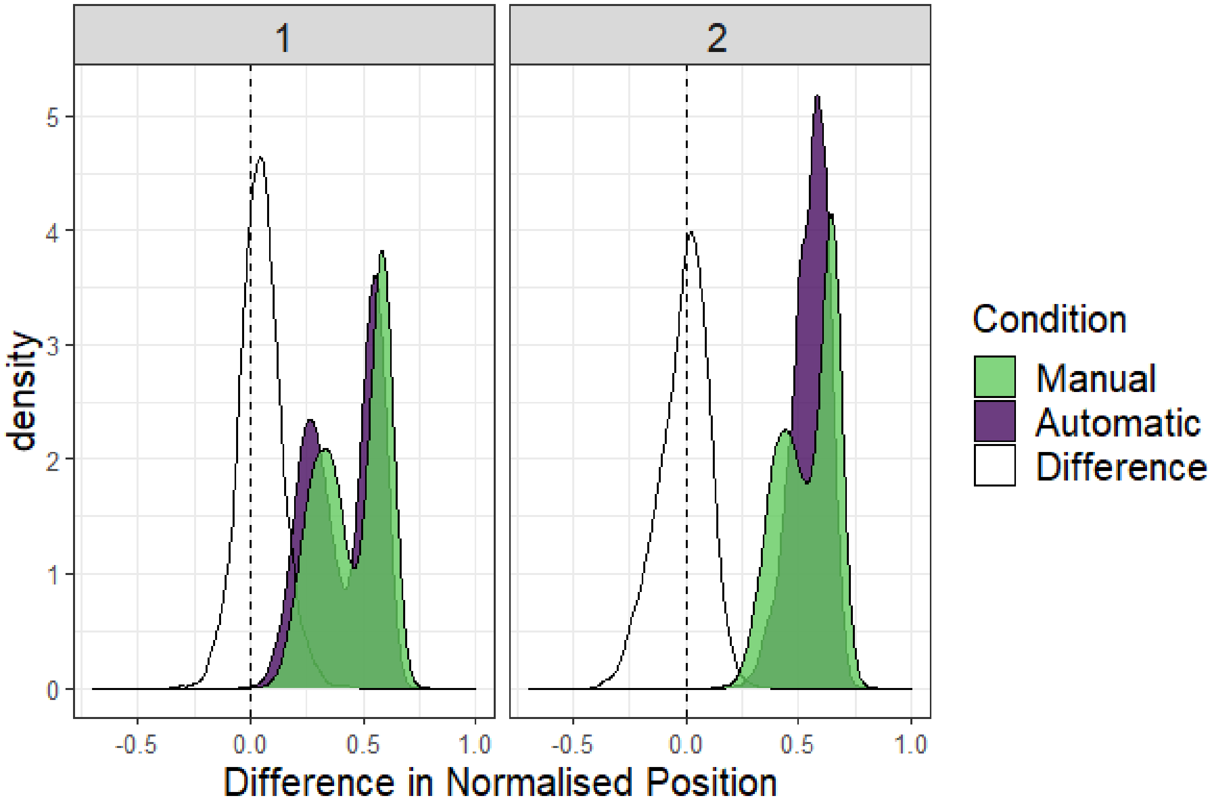

Note. The posterior predicted adjustment effect (the difference between the Far and Close conditions) for blocks 1 and 2. The white distributions show the difference in adjustment of position with distance between Manual and Automatic conditions.

Confidence-Adjustment correlations by Speed type

Figure S23 shows the relationship between confidence and adjustment in Experiment 2 separated by Speed type. The correlation was significant in the variable condition, $r(28) = 0.37$, $p = .04$. The correlation was also significant in the constant condition ($r(28) = 0.75$, $p < .001$).

Figure S23

Relationship between adjustment and confidence by speed type

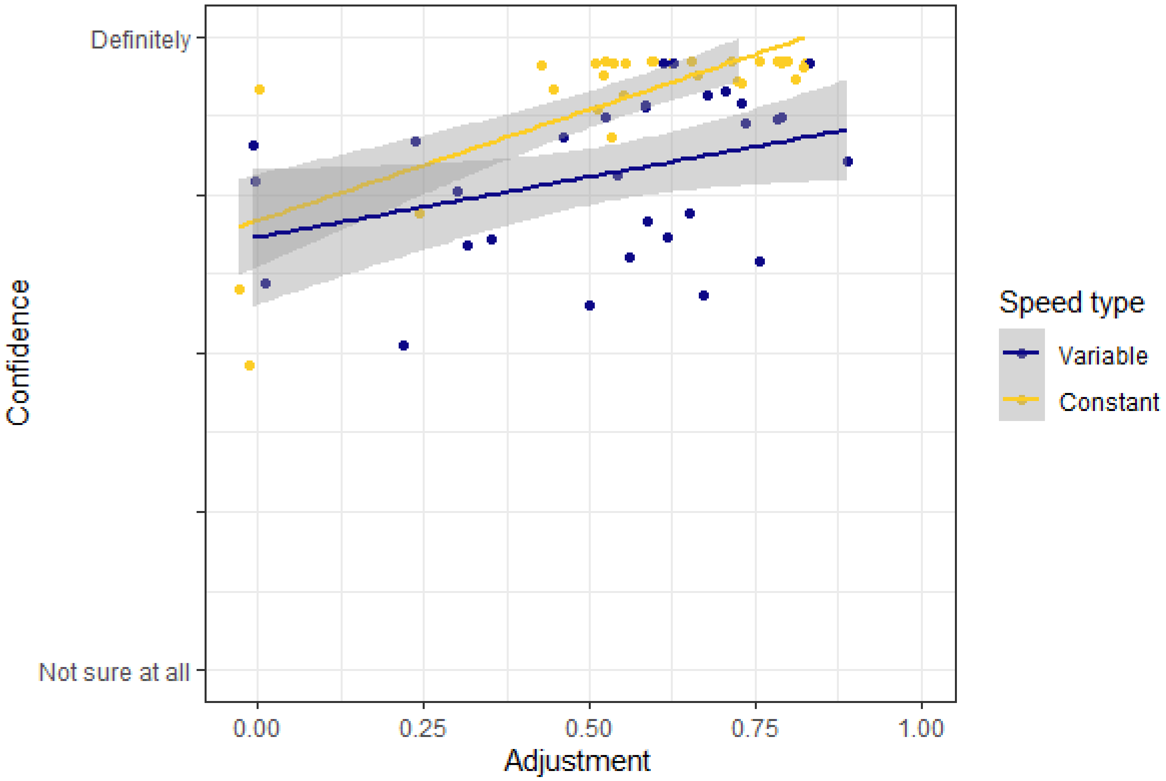

Note. This scatter plot shows the relationship between absolute confidence ratings and the adjustment effect (Far-Close) in Experiment 2, separately by Speed type. Participants were asked “Will the avatar reach its target?”, and positioned a slider on a continuous scale between “Not sure at all” and “Definitely sure”. Each individual contributed two points to the plot as Speed type was manipulated within-subjects.

Correlation between difference in adjustment and difference in confidence

Figure S24 shows the relationship between the difference in confidence (Variable - Constant) and difference in adjustment (Variable - Constant). The correlation *r*(28) = 0.31, was not significant at alpha = .05.

Figure S24

Relationship between difference in adjustment and Confidence by Speed type

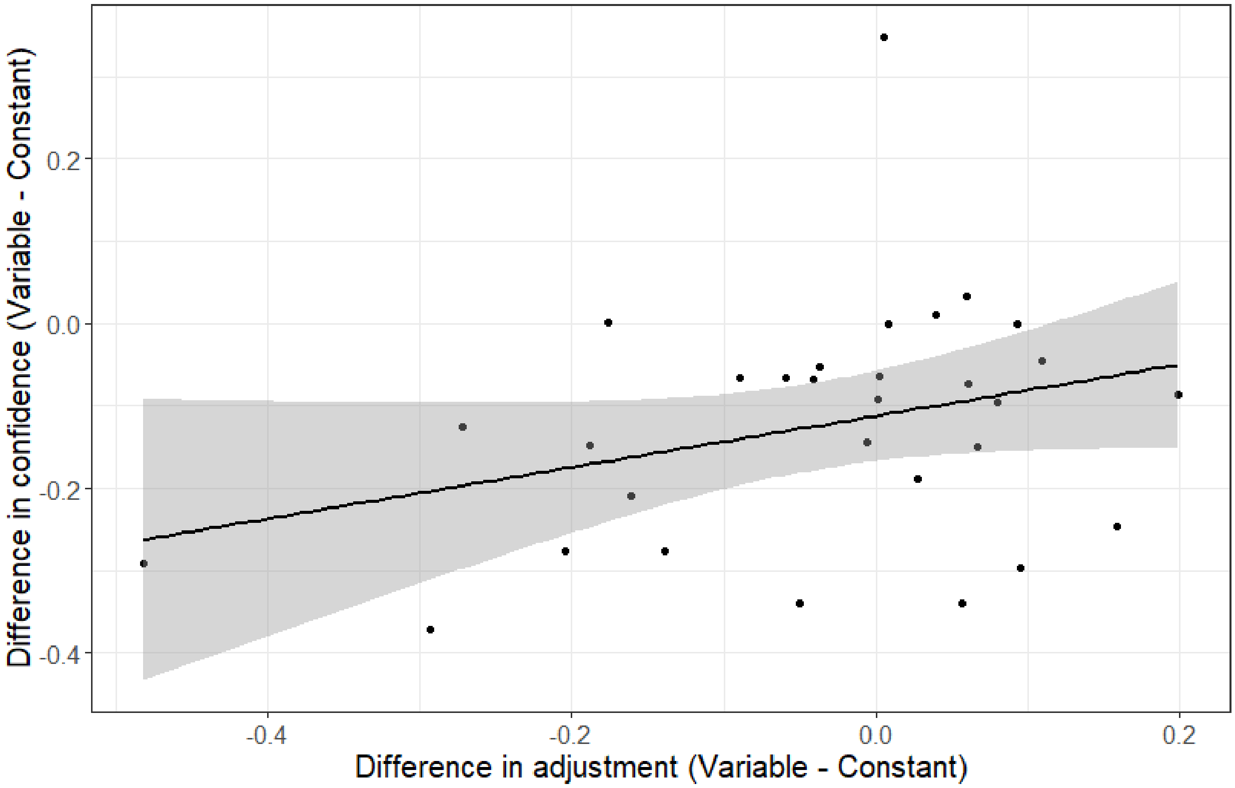

Note. This scatterplot shows the relationship between the difference in adjustment (Variable - Constant) and Confidence. For the confidence measure, participants were asked “Will the avatar reach its target?”, and positioned a slider on a continuous scale between “Not sure at all” and “Definitely sure”.

Adjustment-Confidence correlation

Figure S25 shows the relationship between mean confidence ratings in the estimation phase and size of adjustment in the decision phase. The strongly positive correlation $r(28) = .65$, $p < .001$, indicates that the greater confidence participants had in the outcome, the more they tended to adjust truck position with the change in distance from Close to Far. The relationship separated by Speed type can be seen in supplementary material section B.

Figure S25

Scatter plot of confidence ratings against adjustment of truck position

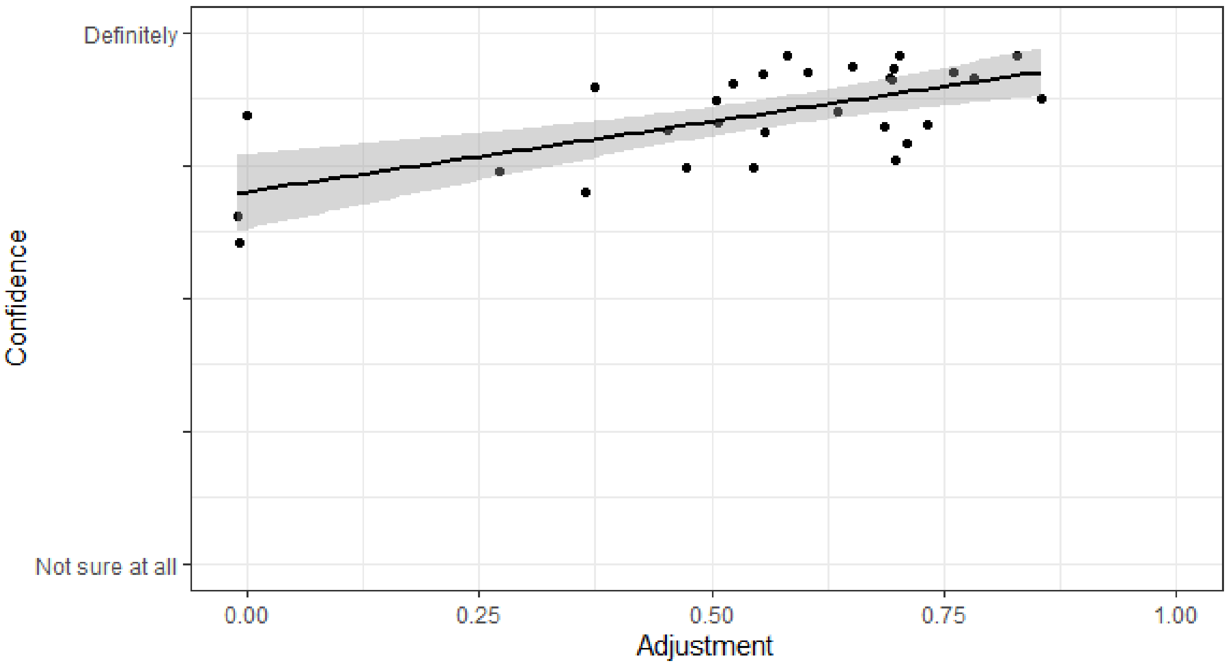

Note. This scatter plot shows the relationship between absolute confidence ratings and the adjustment effect (Far-Close) in Experiment 2. Participants were asked “Will the avatar reach its target?”, and positioned a slider on a continuous scale between “Not sure at all” and “Definitely sure”.

Section C: Supplementary material for Experiment 3

Estimation phase

Figure S26

The learning phase of Experiment 3

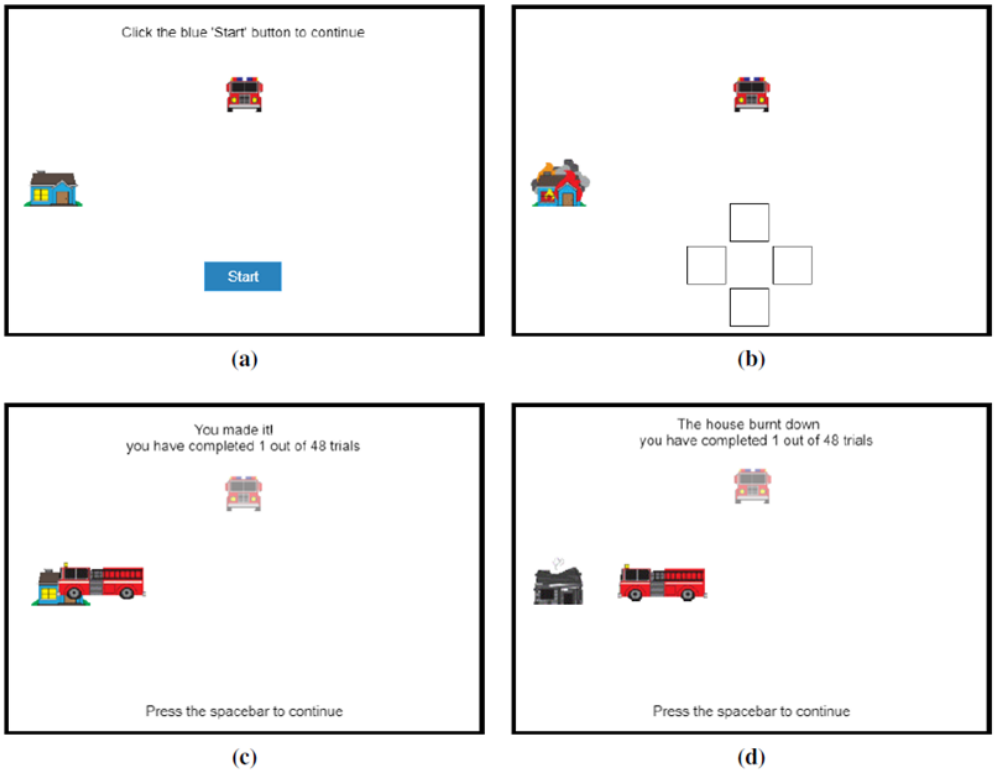

Note. This figure shows each phase of a trial in the Learning phase of Experiment 3. a) shows the initial screen. b) shows the screen once the house had begun to burn. c) and d) show the outcome if participants had succeeded or failed in reaching the target in the time allowed.

Figure S27

The estimation phase of Experiment 3

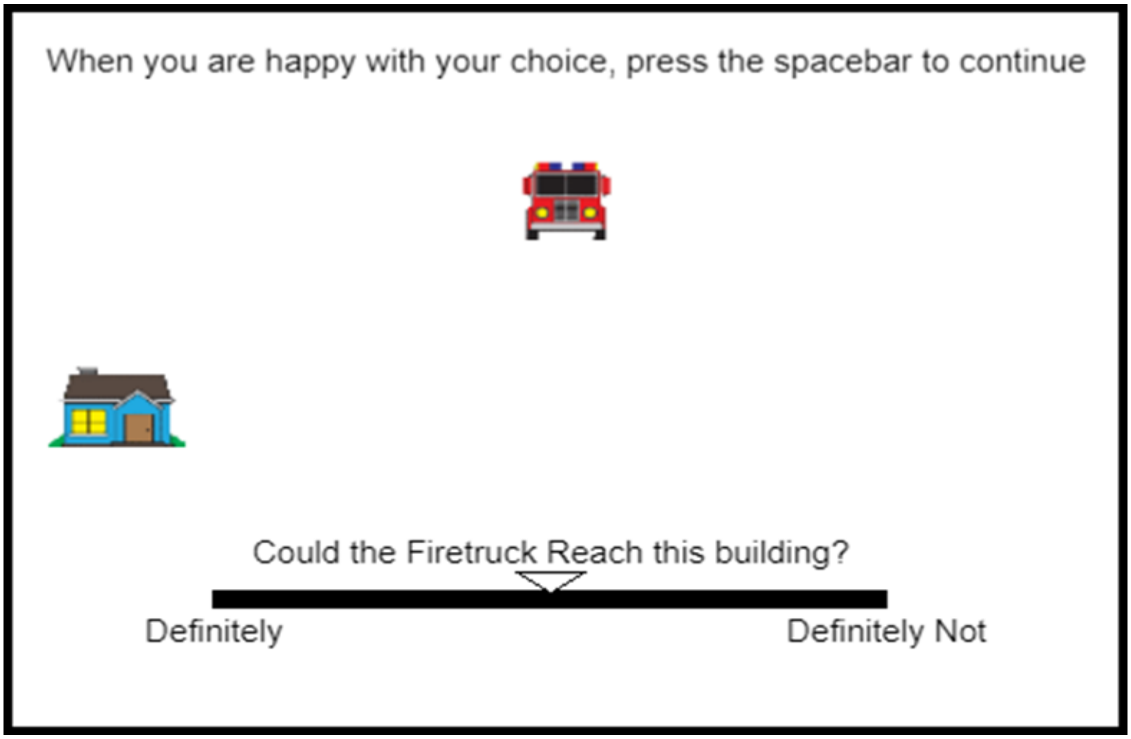

Note. This figure shows the display in the Estimation phase of Experiment 3.

Figure S28

Results from the estimation phase of Experiment 3

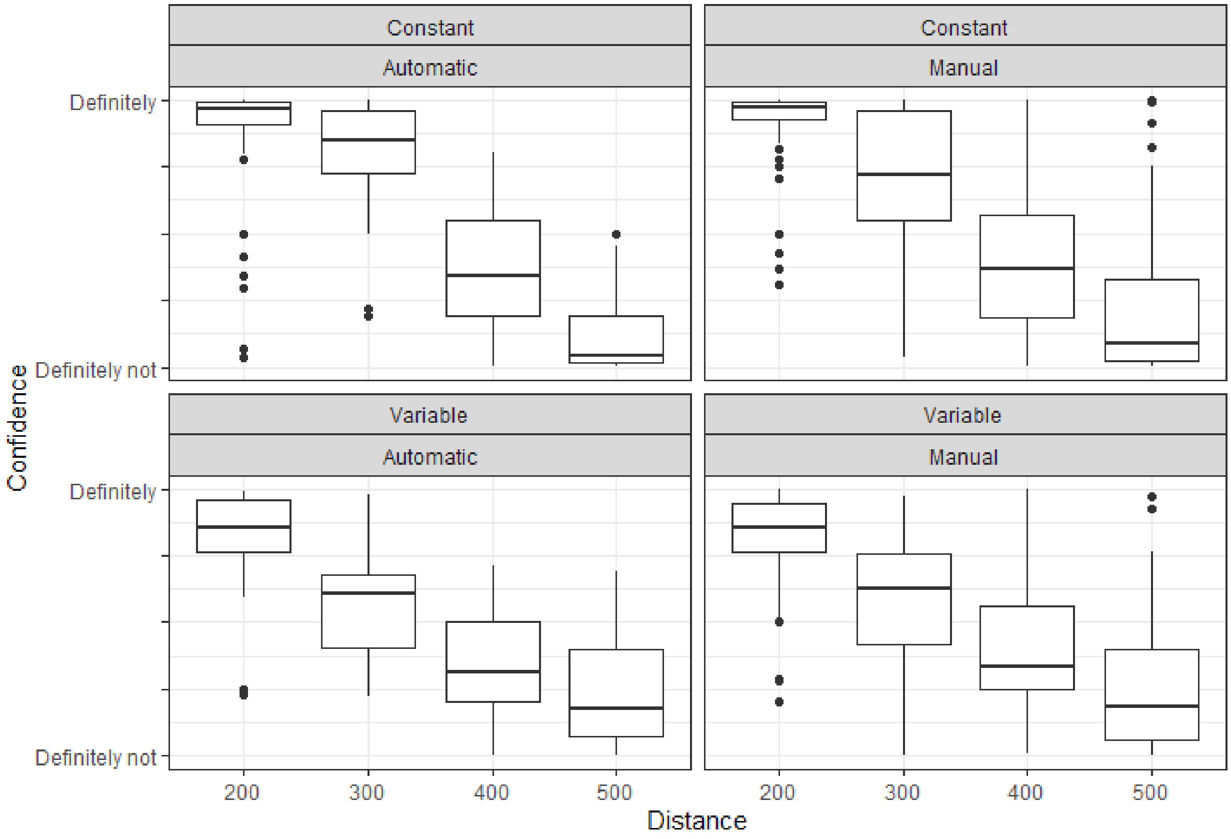

Note. Results from the estimation phase of Experiment 3, showing mean confidence ratings that the truck would reach the target from a given delta on a continuous scale from “Definitely” to “Definitely not”.

Learning phase check

As for Experiments 1 and 2 (in supplementary sections A and B), a Bayesian binomial regression was performed on the Learning phase accuracy data to check the conditions were comparable in terms of expected accuracy over distance to make sure each of the distances used in the decision phase were comparable in terms of the optimal strategy. That is, that the accuracy at the two closest distances was above 50%, and below 50% at the two furthest distances. The normalised distances for this check were 0.4 (closest), 0.6 (close-middle), 0.8 (far-middle), and 1 (furthest). The results of this model can be seen in Figure S29 which demonstrates that there was a difference across the conditions in terms of accuracy by distance. This difference was most pronounced in the variable condition, in which the optimal strategy would differ at the close-middle distance, meaning comparisons at this distance in the decision phase would not be valid. However, the optimal strategy for the closest and furthest distances remained the same across conditions. Participants should have placed the firetruck in the middle when the houses were closest together, and adjacent to one of the houses when they were furthest apart. As such, it is still appropriate to make comparisons in terms of truck placement for these distances.

Figure S29

Results of the learning phase model

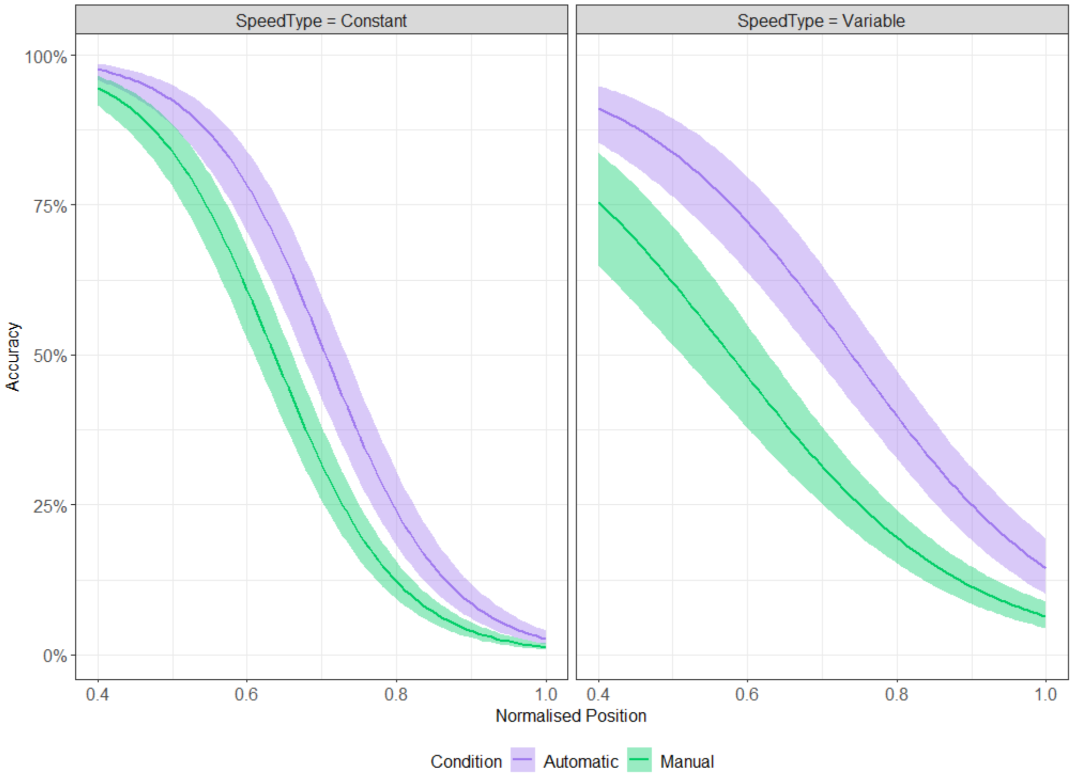

Note. These plots show the conditional effects of a Bayesian binomial regression fit to the Learning Phase data in each uncertainty (Constant or Variable) and agency (Manual vs Automatic) condition.

Decision phase

Figure S30

Normalised truck positions chosen in Experiment 2

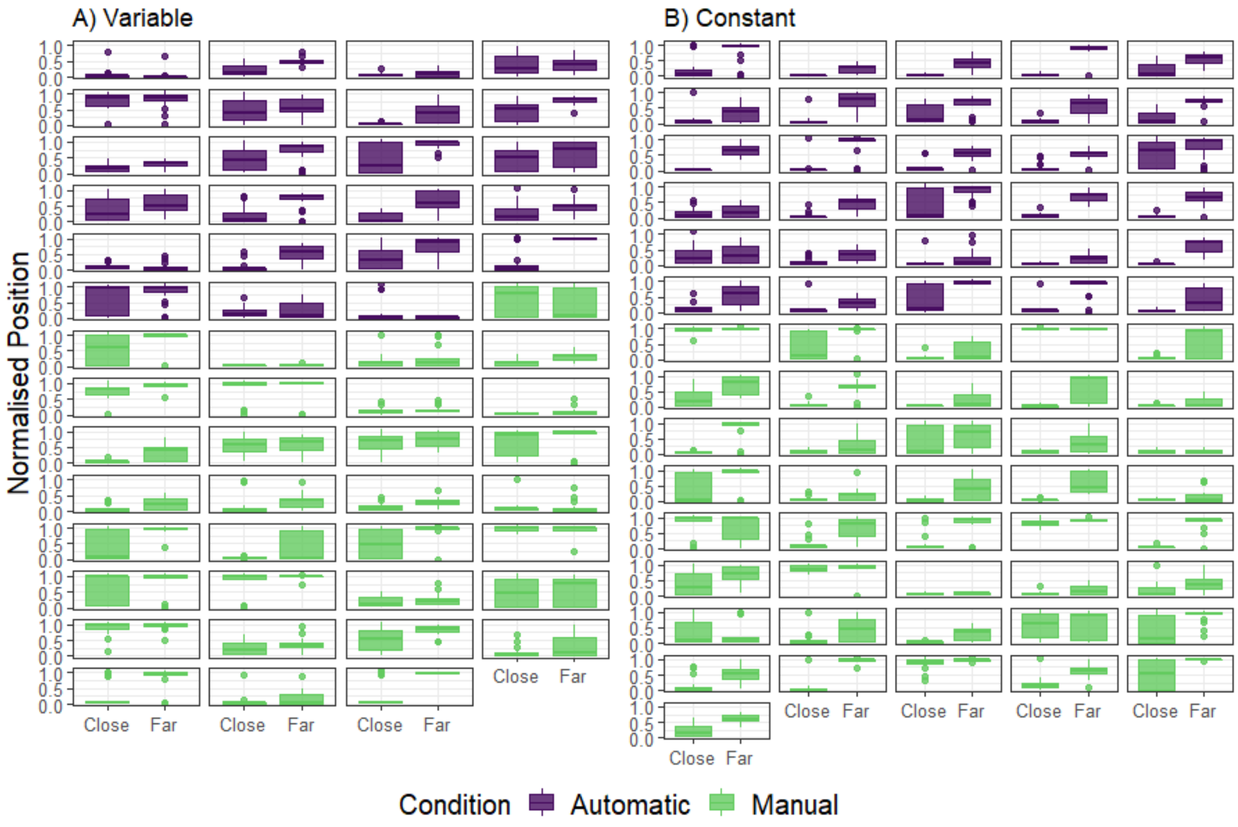

Note. Facets show the normalised truck positions chosen by each participant in the Variable (A) and Constant (right) conditions of Experiment 2, coloured separately by agency condition (Automatic or Manual).

Figure S31

Normalised position including the two middle distances in Experiment 3

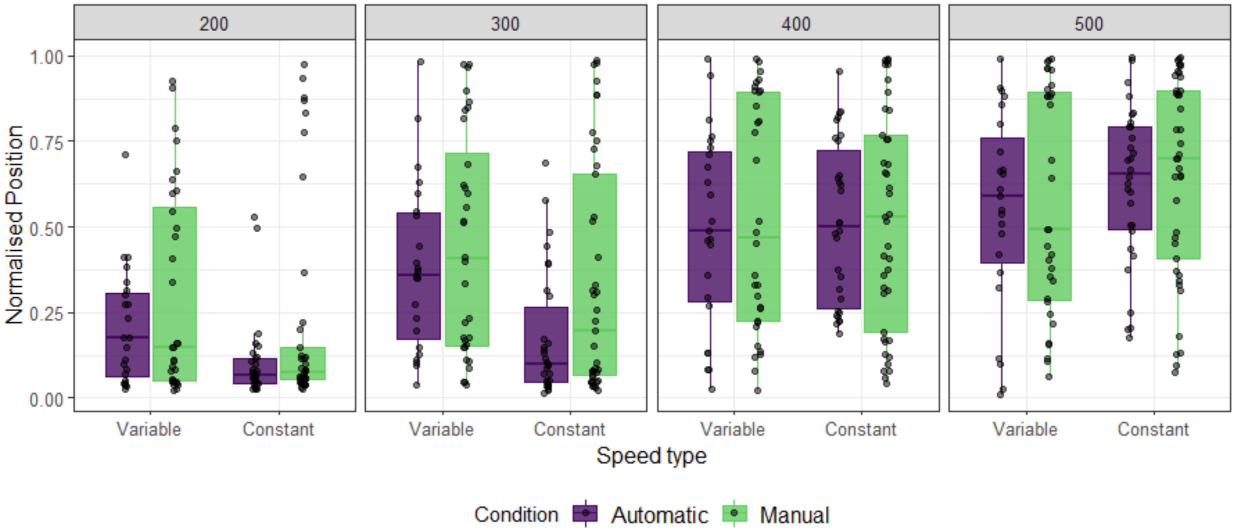

Note. Summary plots for the decision phase data in Experiment 3, including the two middle distances between houses. The dots show the mean normalised truck positions chosen by each participant in the Constant and Variable uncertainty conditions, coloured separately by agency condition (Automatic or Manual).

Figure S32

Model predictions for Experiment 3 including random effects

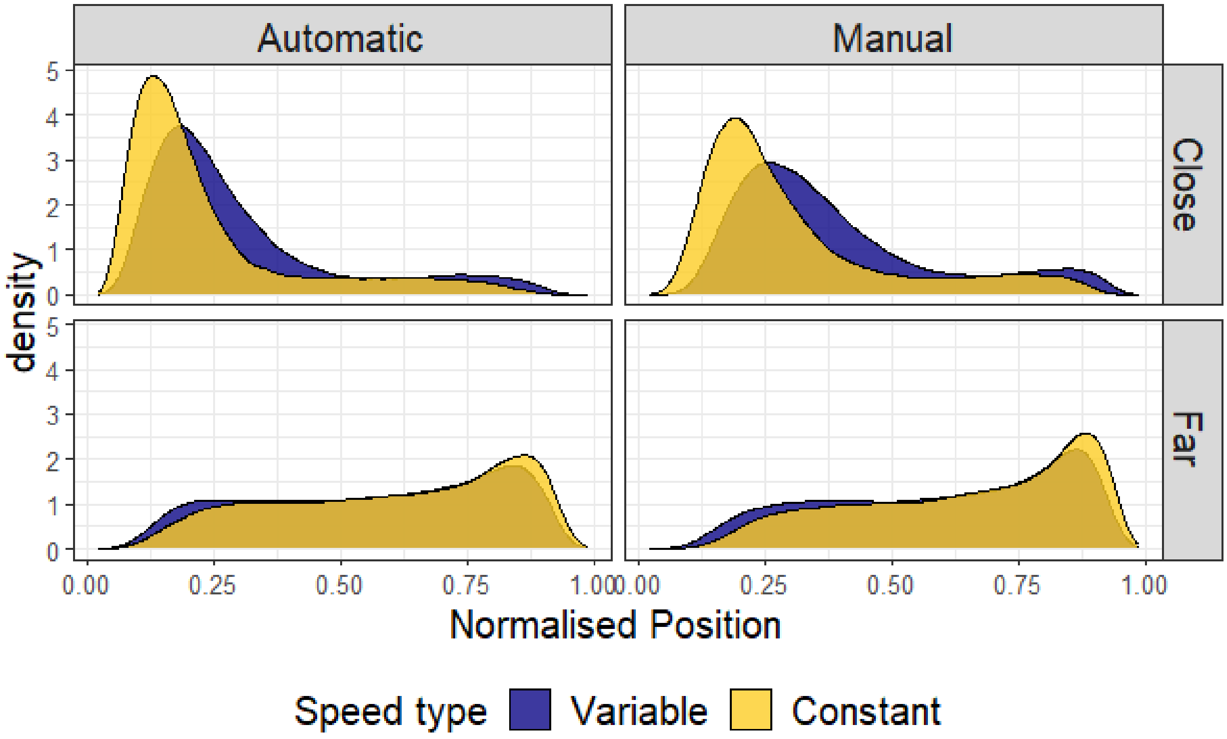

Note. These plots show the posterior predicted means of the Bayesian beta regression model fit to the decision data in Experiment 3, including the random effects structure.

Additional exploratory comparison

Additional exploratory comparisons indicated that there was no interaction between uncertainty and agency (Figure S33). There was no difference in adjustment between the Manual and Automatic conditions when the truck's speed was Variable (mean difference = -0.06, 95% HDI |-0.20, 0.11|) or Constant (mean difference = -0.02, 95% HDI |-0.15, 0.09|).

Figure S33

The adjustment effect interaction in Experiment 3

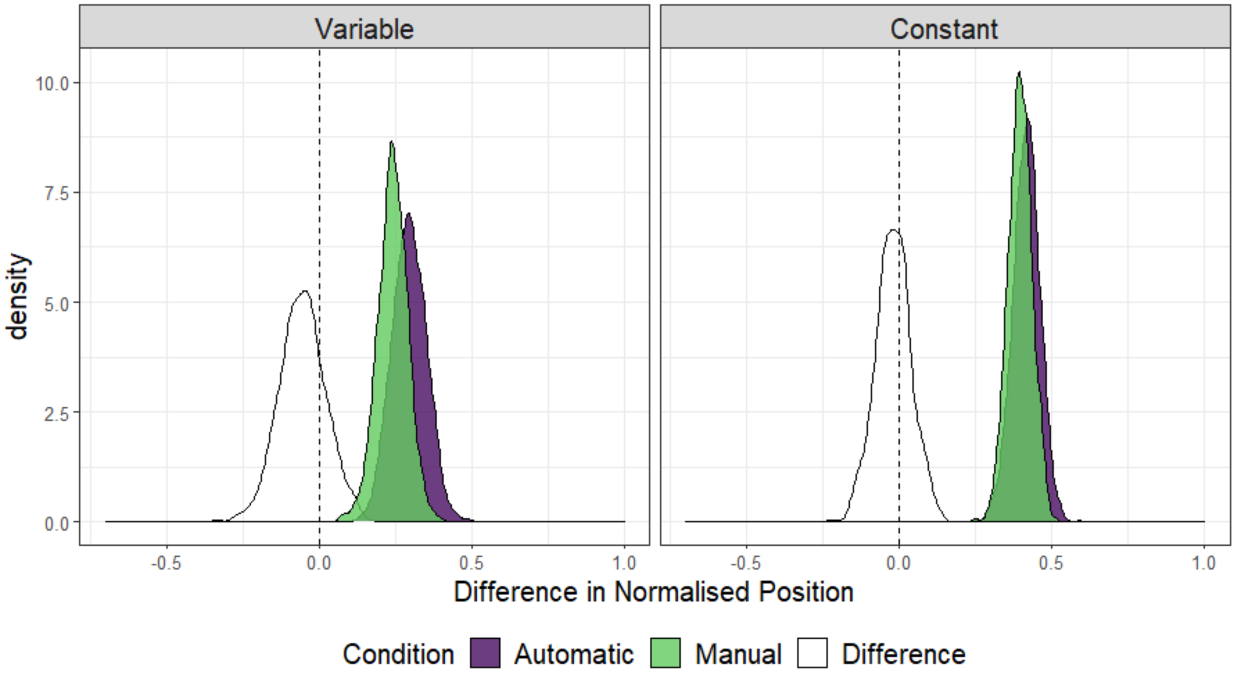

Note. The plots show the adjustment (Far-Close) of position by agency condition (Automatic or Manual), at each level of uncertainty (Variable or Constant), with the difference in adjustment shown in grey.

Adjustment-confidence correlation

Figure S34 shows the moderate positive correlation between absolute confidence ratings for the closest and furthest distances in the estimation phase with adjustment of truck position in the decision phase, $r(124) = .37$, $p < .001$. Similarly to Experiments 1 and 2, the greater confidence participants had in the outcome of the trial, the more they adjusted position with the change in distance from Close to Far. The relationship separated by speed type can be seen in supplementary section C.

Figure S34

Scatter plot of confidence ratings against adjustment of truck position

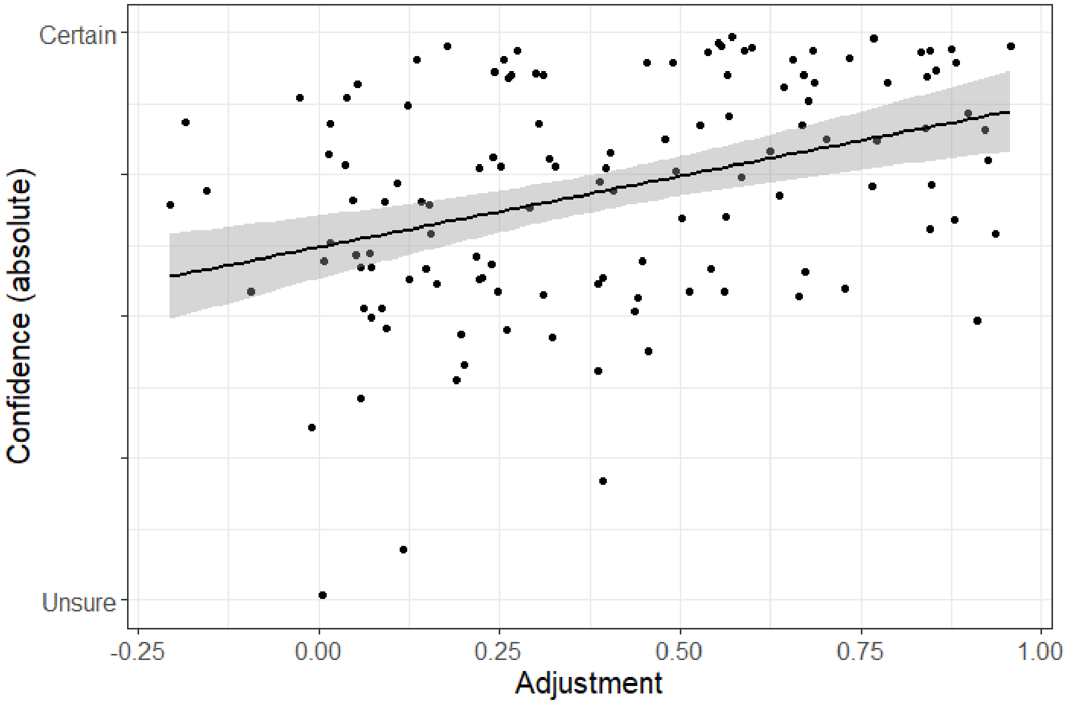

Note. This scatter plot shows the relationship between absolute confidence ratings and the adjustment effect (Far-Close) in Experiment 3. Confidence reflects the mean rating that the truck would reach the target from a given distance on a continuous scale between “Definitely” and

"Definitely not". Absolute values were taken such that "Definitely" and "Definitely not" reflected the maximum level of confidence, labelled "Certain" for the purposes of plotting. The midpoint was labelled "Unsure" to aid interpretation of the plot.

Confidence-Adjustment correlations by Speed type

Figure S35 shows the relationship between confidence and adjustment in Experiment 3 separated by Speed type. The correlation was significant in the Variable group, $r(69) = 0.35$, $p = .002$. The correlation in the constant group ($r(53) = 0.21$) was not significant at alpha = .05.

Figure S35

Relationship between adjustment and confidence by speed type

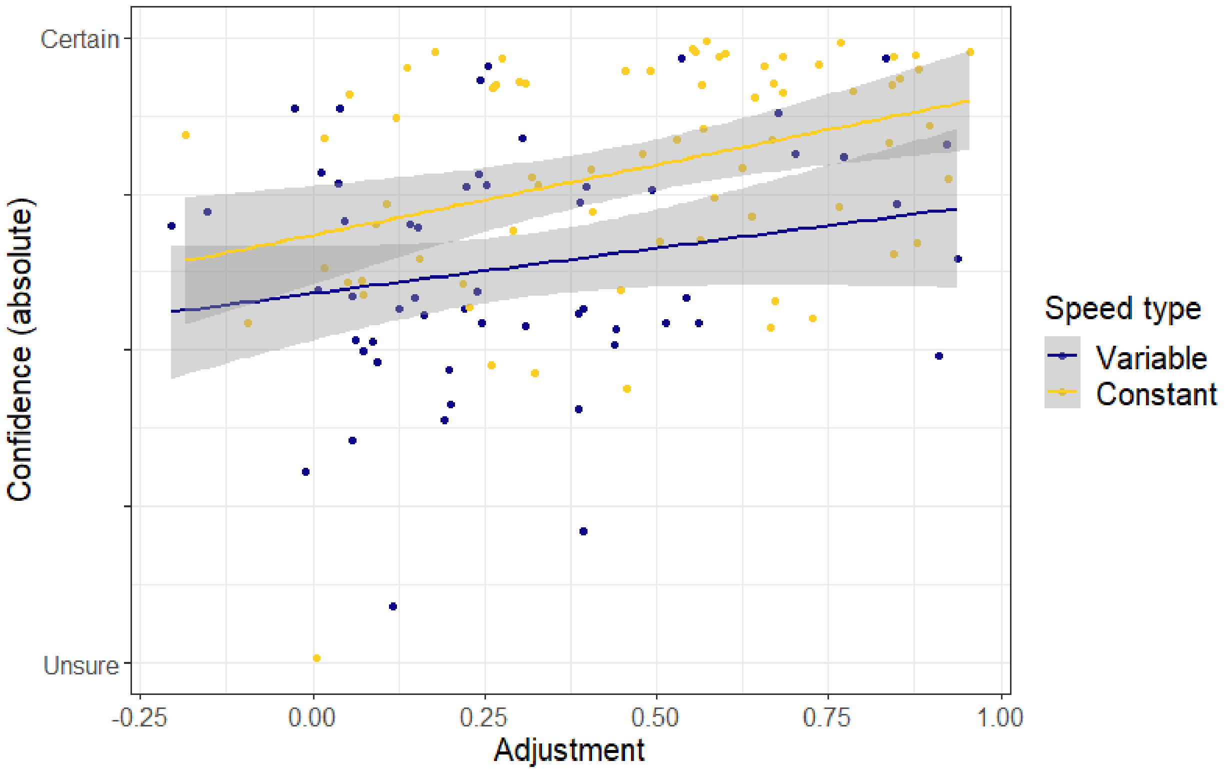

Note. This scatter plot shows the relationship between absolute confidence ratings and the adjustment effect (Far-Close) in Experiment 3, separately by Speed type. Confidence reflects the mean rating that the truck would reach the target from a given distance on a continuous scale between "Definitely" and "Definitely not". Absolute values were taken such that "Definitely" and "Definitely not" reflected the maximum level of confidence, labelled "Certain" for the purposes of plotting. The midpoint was labelled "Unsure" to aid interpretation of the plot.

Success rate analysis

The learning phase model described in section C was used to generate predicted success rates depending on truck positions chosen by participants in the decision phase of Experiment 3. Figure S36 shows these predictions, as well as predictions if participants had followed either the optimal strategy (focus when far, divide when close) or had always positioned the truck in the middle of the screen. Although the optimal strategy would have yielded a higher success rate, actual decisions of participants were reasonably close, and improved over a hypothetical, 'always middle' strategy.

Figure S36

Success rate predictions from the learning phase model

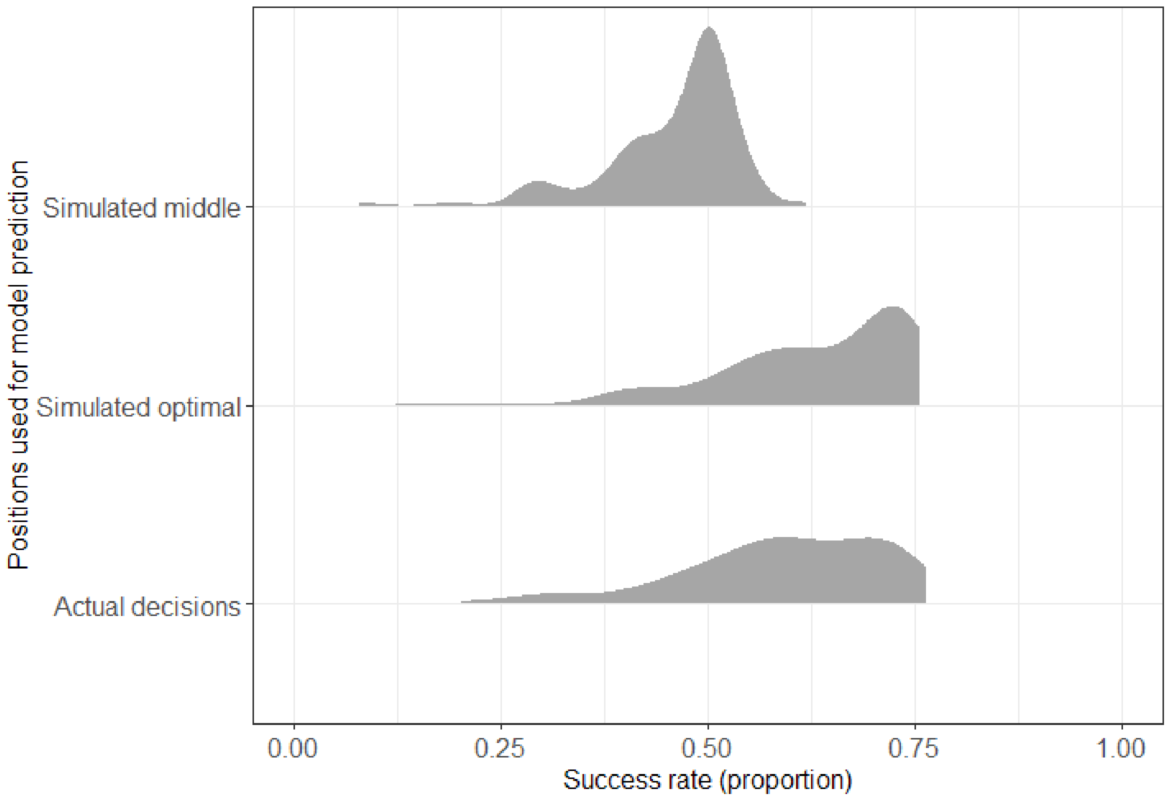

Note. Success rate predictions generated using the learning phase model described in section C. The top two rows show predictions for two hypothetical strategies: always placing the truck in the middle (top) or following the optimal strategy (middle). The bottom row shows the predicted success rate for actual positions chosen by participants in the decision phase of Experiment 3.
